# Selective Inhibition of Integrin β3 Topology Provides a Safer Antithrombotic Strategy

**DOI:** 10.1002/advs.202522086

**Published:** 2026-04-13

**Authors:** Joonha Lee, Chul‐Gyun Lim, Pothiappan Vairaprakash, Jong‐Min Kim, Jiyoon Kim, Ji‐Young Park, Klaus M. Hahn, Hyunbo Shim, Hae Woong Choi, Tobias S. Ulmer, Soon Jun Hong, Chungho Kim

**Affiliations:** ^1^ Department of Life Sciences Korea University Seoul Republic of Korea; ^2^ Department of Pharmacology University of North Carolina at Chapel Hill Chapel Hill North Carolina USA; ^3^ Department of Clinical Pharmacology and Toxicology Anam Hospital Korea University College of Medicine Seoul Republic of Korea; ^4^ Department of Biochemistry University of Toronto Toronto Canada; ^5^ Department of Life Science Ewha Womans University Seoul Republic of Korea; ^6^ Department of Physiology and Neuroscience Zilkha Neurogenetic Institute Keck School of Medicine University of Southern California Los Angeles California USA; ^7^ Department of Cardiology Cardiovascular Center Korea University Anam Hospital Korea University College of Medicine Seoul Republic of Korea

**Keywords:** integrin αIIbβ3, mechanical force, platelet aggregation, shear stress, thrombosis, transmembrane domain

## Abstract

Cardiovascular and cerebrovascular diseases, the leading causes of death worldwide, are primarily driven by pathological thrombus formation resulting from uncontrolled platelet aggregation at sites of atherosclerotic plaque rupture. Current antiplatelet drugs are designed either to indirectly inhibit intracellular signaling pathways that activate integrin αIIbβ3 or to directly block the binding of the activated integrin to fibrinogen. However, because this interaction is also essential for normal hemostasis, these drugs inevitably increase the risk of serious spontaneous bleeding. In this study, we aimed to develop a safer strategy to prevent thrombosis without impairing hemostasis. Building on our previous findings that the transmembrane domain (TMD) of integrin β3 undergoes distinct topological changes during force‐ vs. agonist‐dependent activation, we targeted the β‐tail domain of integrin β3 as an epitope for an antibody that selectively blocks force‐induced changes. This antibody specifically inhibits force‐dependent αIIbβ3 activation, a key driver of platelet aggregation at atherosclerotic lesions, and suppresses platelet aggregation in ex vivo and in vivo models without inducing bleeding. From these results, we conclude that this approach offers a promising therapeutic strategy to prevent thrombosis while minimizing bleeding risk, addressing a major limitation of current antiplatelet therapy.

## Graphical abstract

Current integrin αIIbβ3 inhibitors effectively reduce thrombosis but also increase bleeding risk. During thrombosis, high shear blood flow can directly activate the integrin αIIbβ3 via a distinct topological change in the β3 transmembrane domain, independent of hemostatic platelet signaling. Here, we show that blocking this topological change with integrin β3 β‐tail domain‐specific antibody selectively inhibits thrombosis while preserving normal hemostasis.

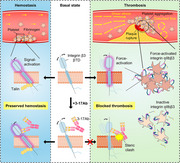

## 1 Introduction

Platelets play a central role in hemostasis by orchestrating a response that leads to the cessation of bleeding. Upon stimulation by physiological agonists such as thrombin, collagen, or adenosine diphosphate (ADP), intracellular signaling cascades are activated within platelets, triggering the activation of the platelet adhesion receptor integrin αIIbβ3, also known as glycoprotein (GP) IIb/IIIa [1, 2]. Activation induces a conformational change involving disruption of the transmembrane domain (TMD) interaction between the α and β subunits, converting the integrin into a high‐affinity state capable of binding to its ligand, fibrinogen [3, 4]. Fibrinogen, a dimer composed of αβγ heterotrimers [5], contains multiple integrin‐binding sites [6], allowing a single molecule to engage integrins on adjacent platelets simultaneously. This multivalency facilitates platelet cross‐linking and aggregation [7, 8]. Although platelet aggregation is essential for maintaining hemostasis, its dysregulated activation at sites of vascular injury, such as atherosclerotic plaques, can lead to pathological thrombosis and vascular occlusion [9]. In coronary and cerebrovascular arteries, this can cause ischemic injury to the heart or brain—major causes of death worldwide [10].

Given its critical role in platelet aggregation, direct inhibition of integrin αIIbβ3 binding to fibrinogen—using drugs such as eptifibatide, abciximab, or tirofiban that target its fibrinogen‐binding sites—has been established as a potent antithrombotic strategy, particularly in acute coronary syndrome (ACS) and during percutaneous coronary intervention (PCI) [11, 12, 13, 14]. However, due to their mechanism of action, these agents cannot distinguish between pathological and physiological platelet aggregation, thereby impairing normal hemostasis [14, 15, 16, 17]. As a result, stronger inhibition of platelet aggregation correlates with a higher risk of bleeding. To reduce this risk, mild inhibition of platelet aggregation through antiplatelet agents that target upstream signaling pathways leading to integrin αIIbβ3 activation—such as aspirin, which inhibits thromboxane A_2_ generation [18, 19], and clopidogrel, which blocks ADP receptor binding [20]—is widely used in clinical practice. However, bleeding remains an unavoidable side effect, limiting the clinical utility of these drugs, particularly in patients at high risk of hemorrhage or those requiring surgery. These challenges underscore the need for next‐generation therapies that achieve effective yet safer antithrombotic outcomes.

Recent studies have highlighted the critical role of abnormal hemodynamic forces at sites of atherosclerotic plaque rupture as a primary driver of exaggerated platelet aggregation and pathological thrombosis [21, 22]. A pioneering study demonstrated that platelet aggregation can be primarily driven by shear stress under high shear conditions, with soluble agonists playing a secondary role in stabilizing the formed aggregates [23]. Consistently, platelet aggregation at stenotic regions was not inhibited by blocking agonist‐induced intracellular signaling pathways that activate integrin αIIbβ3. In contrast, direct inhibition of integrin–fibrinogen interactions using an anti‐αIIbβ3 antibody effectively impaired platelet aggregation, indicating that integrin αIIbβ3 is essential for aggregation, but its activation under shear stress occurs independently of canonical signaling pathways. A recent clinically relevant microfluidic study provided additional evidence supporting the critical role of shear stress in platelet aggregation and thrombosis [24].

We previously demonstrated that integrin αIIbβ3 can be directly activated by mechanical forces such as shear stress or membrane stretch [25]. This force‐dependent activation does not require conventional platelet signaling and was observed even in a reconstitution system using liposomes containing purified αIIbβ3 subjected to osmotic stretch. This finding provides a mechanistic explanation for shear stress–dependent, signaling‐independent thrombosis at sites of arterial stenosis. Notably, intracellular signaling–dependent activation increases the tilt angle of the αIIbβ3 TMD relative to the membrane normal [26], whereas mechanical force reduces the tilt angle while still weakening the TMD interaction between αIIb and β3 [25], ultimately leading to integrin activation [27]. Based on this mechanistic distinction between mechanical force–mediated and agonist‐mediated integrin activation, we hypothesized that selectively targeting the mechanically induced TMD movement—specifically, the force‐induced reduction in β3 TMD tilt angle—could offer a therapeutic strategy to inhibit pathological thrombosis without impairing normal platelet function.

**FIGURE 1:**
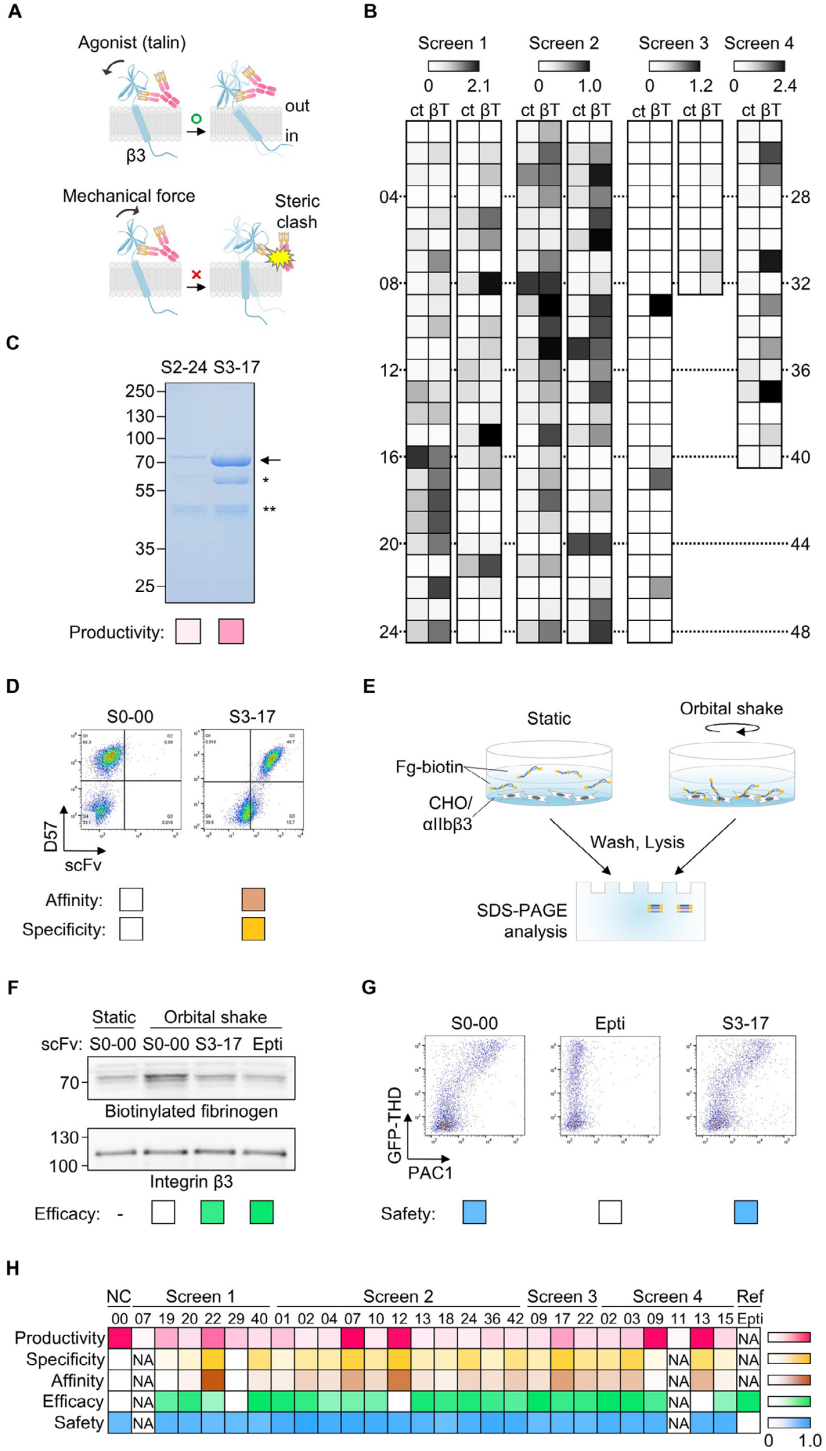
Phage display screening for scFv clones against the β‐tail domain of integrin β3. (A) A hypothetical diagram illustrating a TMD topology‐specific blocker. (B) Positive scFv clones from four independent phage display screens were tested for binding to purified GST (control, ct) or GST‐βTD (βT). Binding levels were measured by ELISA and displayed as a white‐to‐black gradient, as indicated for each screen. Clone numbers are indicated to the left (04‐24) and right (28‐48) of the columns. (C) SDS‐PAGE analysis of representative clones purified as MBP fusions (arrow). Purification yields are indicated by a pink gradient. The bands indicated with the single (*) and double asterisk (**) are contaminants, presumably MBP‐V_H_ and MBP, respectively. (D) Flow cytometry plots showing scFv (2 µm) binding to CHO and CHO/αIIbβ3 cells. Anti‐HA antibody detected scFv binding, and D57, an integrin αIIbβ3‐specific antibody, distinguished CHO/αIIbβ3 cells. Affinity (mean fluorescence of CHO/αIIbβ3 binding minus that of control clone S0‐00) and specificity (CHO/αIIbβ3‐to‐CHO binding ratio) are shown as brown and orange gradients, respectively. (E) Schematic representation of the orbital shaking‐induced fibrinogen (Fg) binding assay. (F) Representative results showing cell‐bound fibrinogen (upper) in the presence of 2 µm scFv or 20 µm eptifibatide (Epti). The integrin β3 blot is shown as a loading control (bottom). The degree of inhibition of fibrinogen binding under orbital shaking by each scFv, relative to S0‐00, is defined as efficacy and is shown as a green gradient. (G) CHO/αIIbβ3 cells transfected with GFP‐talin head domain (THD) were stained with PAC1, an active αIIbβ3‐specific antibody, in the presence of 2 µm S3‐17 or 20 µm eptifibatide and analyzed by flow cytometry. Specific PAC1 binding to GFP‐positive cells for each scFv was normalized to S0‐00 and defined as “safety,” represented by a green gradient. (H) All unique clones from the screenings in (B) were analyzed for productivity, specificity, affinity, efficacy, and safety, and are summarized in the table.

To test this hypothesis, we aimed to develop an antibody that binds to the membrane‐proximal β‐tail domain (βTD) of the integrin β3 subunit and prevents the force‐induced decrease in TMD tilt angle by inducing steric hindrance with the plasma membrane, while still permitting talin‐dependent tilt angle increase (Figure 1A). We reasoned that such an antibody would selectively inhibit pathological thrombosis driven by mechanical forces at sites of vascular occlusion or atherosclerotic plaque rupture while minimizing bleeding complications. To this end, we employed a previously established single‐chain variable fragment (scFv) phage display library [28] and performed functional screening under orbital shaking conditions to identify candidate binders. Subsequent biochemical and physiological analyses led to the development of a monoclonal antibody, 3–17Ab, which exhibited potent antithrombotic activity without impairing agonist‐dependent platelet activation or increasing bleeding risk. These findings support the concept that selective restriction of force‐induced β3 TMD movement represents a promising strategy for antithrombotic therapy.

## 2 Results

### 2.1 Screening of scFv Clones that Specifically Inhibit Force‐Induced Integrin β3 Activation

To test our hypothesis that an antibody targeting the membrane‐proximal βTD domain may inhibit the force‐induced tilt angle change of the TMD (Figure 1A), we first prepared a βTD peptide as the antigen. To restrict antibody binding to the membrane‐facing region and maximize steric hindrance, the C‐terminal 57 amino acids (Arg636–Asp692) of βTD (Figure S1A,B) were fused to glutathione‐S‐transferase (GST), purified (Figure S1C), and used to screen for binders from a scFv phage display library [28]. Following four independent library screens, individual scFv‐displaying phage clones, as periplasmic extracts, were tested for antigen binding (Figure 1B), resulting in the identification of 26 unique scFv clones. Those were then fused with an N‐terminal maltose‐binding protein (MBP) tag and C‐terminal 6xHis and HA tags, expressed in *E. coli*, and purified using nickel‐nitrilotriacetic acid (Ni‐NTA) affinity chromatography (Figure 1C). The relative yield of each clone was used as a measure of productivity. Next, to assess binding specificity and affinity, Chinese Hamster Ovary (CHO) and CHO/αIIbβ3 cells were mixed at a 1:1 ratio, and the binding of the purified scFv clones to these cells were measured. Specificity was determined by calculating the ratio of scFv binding to CHO/αIIbβ3 cells vs. CHO cells, while affinity was assessed by subtracting the signal of a control scFv (S0‐00) from that of each test scFv on CHO/αIIbβ3 cells (Figure 1D; Figure S2A,B). To evaluate the inhibitory effects of each clone on mechanical stress‐induced integrin activation, CHO/αIIbβ3 cells were subjected to orbital shear stress (9.6 dyne/cm^2^) for 20 min in the presence of biotinylated fibrinogen (Figure 1E). When assessed by Western blotting, fibrinogen binding under shear stress was significantly higher than that under static conditions, as expected (Figure 1F). Purified scFv clones were added at 2 µm during shear stress application, and their inhibitory effects on fibrinogen binding were evaluated (Figure 1F; Figure S3A). In each experiment, the fibrinogen band intensity in the presence of S0‐00 was set to 1 (no inhibition), and the background signal was set to 0. The relative intensities observed with other scFv clones were normalized accordingly, and their inhibitory efficacy was calculated by subtracting these values from 1. Notably, most clones exhibited considerable efficacy (Figure S3B). Among those with relatively higher efficacy, the half‐maximal inhibitory concentration (IC_50_) was determined, identifying S2‐18, S3‐17, S2‐24, and S2‐07 as the top‐ranked candidates (Figure S4). To assess their effect on talin‐induced integrin αIIbβ3 activation, PAC1 binding was measured in CHO/αIIbβ3 cells transfected with talin head domain (THD), a constitutively active version of talin [29] (Figure S5). PAC1 binding in the presence of the antiplatelet drug eptifibatide [15, 30] was set to 0, and that in the presence of S0‐00 was set to 1. Values obtained with other scFv clones were normalized accordingly and used as a measure of safety (Figure 1G). Finally, each clone's productivity, specificity, affinity, efficacy, and safety values were normalized and integrated for comparative analysis (Figure 1H).

### 2.2 Selection of scFv Clones With Desirable Properties and Antibody Generation

**FIGURE 2:**
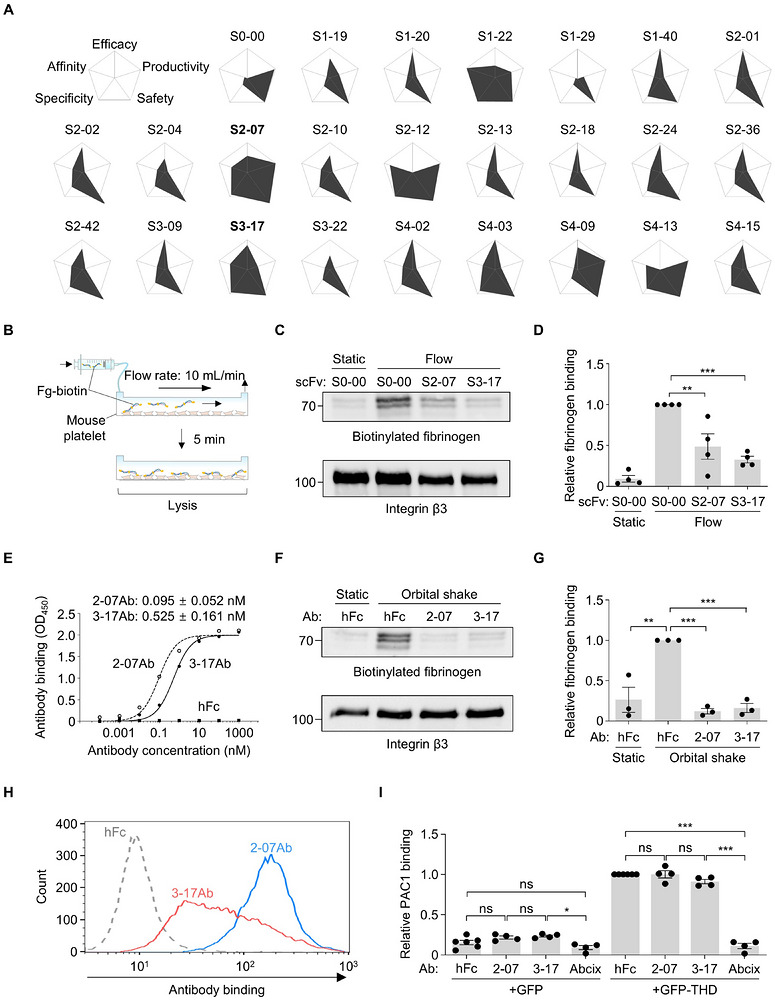
scFv clone selection and antibody production. (A) Petal plots showing five properties of clones identified during screening. Clones with superior properties, S2‐07 and S3‐17, are highlighted in bold. (B) Schematic of the laminar flow‐induced fibrinogen (Fg) binding assay. (C) Mouse platelets attached to a collagen‐coated flow chamber were perfused with HBSS (10 mL/min, 40 dyne/cm^2^) containing biotinylated fibrinogen and 0.5 µm scFv for 5 min. Bound fibrinogen was analyzed as described in Figure 1F. (D) Fibrinogen binding was normalized to the S0‐00‐treated condition and shown as a bar graph (*n* = 4). Error bars indicate the mean ± SEM. ∗∗, *p* < 0.01; ∗∗∗, *p* < 0.001 (one‐way ANOVA using Tukey's multiple comparison test). (E) ELISA analysis of 2–07Ab and 3–17Ab binding to purified β3 βTD. Binding curves were fitted using a one‐site binding model. Control human Fc (hFc) binding is shown as squares. (F,G) Fibrinogen binding to mouse platelets under orbital shaking in the presence of 1 µm of the indicated antibody was analyzed by Western blotting. Quantified fibrinogen binding normalized to control hFc is shown (*n* = 3). Error bars indicate the mean ± SEM. ∗∗, *p* < 0.01; ∗∗∗, *p* < 0.001 (one‐way ANOVA using Tukey's multiple comparison test). (H) Flow cytometry histogram showing antibody binding to CHO/αIIbβ3 cells. (I) CHO/αIIbβ3 cells transfected with GFP or GFP‐THD were stained with PAC1 in the presence of 1 µm of the indicated antibody. Mean PAC1 binding in GFP‐positive cells was measured using flow cytometry and presented as a bar graph (*n* = 6 for hFc; *n* = 4 for others). Error bars indicate the mean ± SEM. ∗, *p* < 0.05; ∗∗∗, *p* < 0.001; ns, not significant (one‐way ANOVA using Tukey's multiple comparison test).

The selected scFv clones exhibited diverse profiles across the five evaluated properties (Figure 2A). Based on the integrated analysis of all five metrics, S2‐07 and S3‐17 were selected for further evaluation. Both clones bound human and mouse βTD (Figure S6A,B) and mouse platelets (Figure S6C) with comparable affinity, although S2‐07 had slightly higher binding affinity. Their inhibitory efficacy was validated again using mouse platelets exposed to laminar flow (10 mL/min) (Figure 2B). Consistent with the orbital shear assay, both S2‐07 and S3‐17 significantly reduced force‐induced fibrinogen binding, with S3‐17 demonstrating superior potency (Figure 2C,D). Specific binding to integrin αIIbβ3 was also confirmed by using CHO cells as a negative control lacking αIIbβ3 expression (Figure S7A). To generate full‐length antibodies, the variable regions of the selected scFvs were grafted onto the human IgG scaffold of IGN‐002 antibody [31], yielding 2–07Ab and 3–17Ab, respectively, and produced via HEK293F cell suspension culture (Figure S8A,B). Based on Enzyme‐Linked Immunosorbent Assay (ELISA)‐derived binding curves, the approximate binding affinities of 2–07Ab and 3–17Ab to human β3 βTD were estimated as 0.095 ± 0.052 nm and 0.525 ± 0.161 nm, respectively (Figure 2E). Consistent with their scFv forms, both antibodies significantly inhibited fibrinogen binding under orbital shear stress (Figure 2F,G) to a degree comparable to that of the positive control abciximab, a monoclonal antibody that directly blocks the αIIbβ3‐fibrinogen interaction [32] (Figure S7B,C). Their binding to human αIIbβ3 expressed in CHO cells (Figure 2H) and to mouse αIIbβ3 in mouse platelets (Figure S8C) was also confirmed. Based on the concentration‐dependent competition of biotinylated 3–17Ab binding to the immobilized β‐tail domain by pre‐incubation with 2–07Ab (Figure S8D), these two antibodies appear to share a common epitope. Because neither antibody recognized β3 under denaturing SDS‐PAGE conditions (Figure S8E), they are likely to bind conformational (non‐linear) epitopes. Notably, no adverse effects were observed, as indicated by their inhibitory effects on THD‐induced integrin αIIbβ3 activation. In contrast, the positive control abciximab significantly suppressed this activation (Figure 2I).

### 2.3 Effect of 3–17Ab on Mechanical Force‐Induced β3 TMD Movement

**FIGURE 3:**
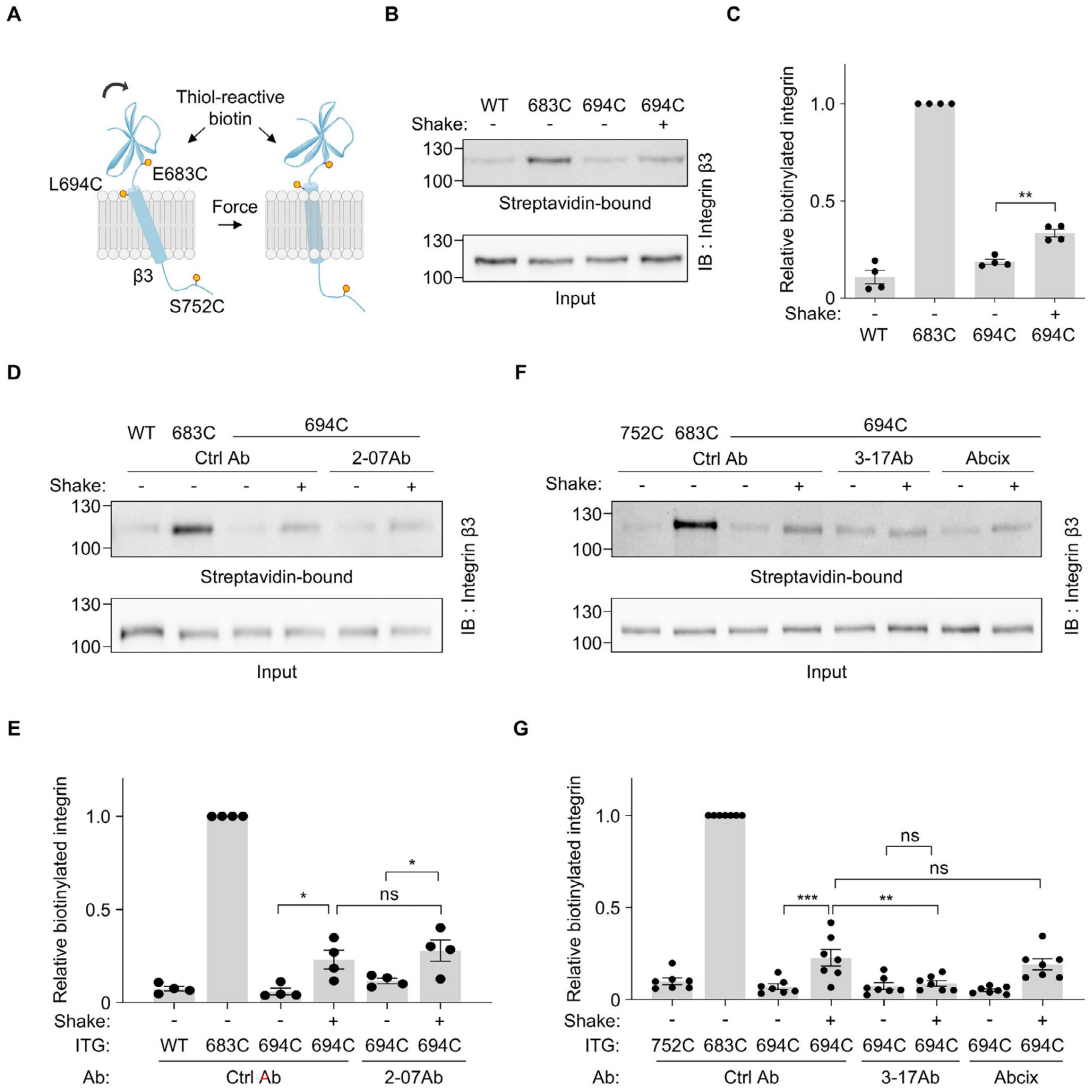
Effects of 3–17Ab on force‐induced exposure of the Leu694 residue in integrin β3. (A) A hypothetic illustration of force‐induced movement of integrin β3 βTD‐TMD. Three residues mutated into cysteines are shown. (B) CHO cells transfected with integrin αIIb and each β3 cysteine mutant were treated with a cell‐impermeable thiol‐reactive biotinylation reagent under static or orbital shaking conditions. After cell lysis, biotinylated integrin β3 was captured using streptavidin‐conjugated beads and analyzed by Western blotting. (C) Quantification of biotinylated integrin β3, normalized to the Glu683Cys mutant β3 (*n* = 4). Error bars indicate the mean ± SEM. ∗∗, *p* < 0.01 (one‐way ANOVA using Tukey's multiple comparison test). (D,E) Effects of 1 µm 2–07Ab on the force‐induced exposure of the Leu694Cys (*n* = 4). Error bars indicate the mean ± SEM. ∗, p < 0.05; ns, not significant (one‐way ANOVA using Tukey's multiple comparison test). (F,G) Effects of 1 µm 3–17Ab or abciximab on the force‐induced exposure of the Leu694Cys were analyzed as described in (D,E) (*n* = 7). Error bars indicate the mean ± SEM. ∗∗, *p* < 0.01; ∗∗∗, *p* < 0.001; ns, not significant (one‐way ANOVA using Tukey's multiple comparison test).

To determine whether the inhibitory effects of the candidate antibodies on force‐dependent integrin activation are mediated through suppression of TMD movement, we established a cell‐based TMD topology monitoring system (Figure 3A). Integrin β3 was engineered to carry a cysteine substitution at Leu694—an amino acid known to shift in and out of the lipid bilayer during topological changes [25, 26]. Additional constructs included Glu683Cys (positive control, extracellularly exposed) and Ser752Cys (negative control, always cytoplasmic). These β3 mutants or wild‐type β3 were co‐transfected with αIIb into CHO cells. A membrane‐impermeable, thiol‐reactive biotin was applied with or without orbital shear. Biotinylated proteins were captured using streptavidin beads and analyzed for β3 content (Figure 3B). Biotinylation of the Leu694Cys mutant modestly but significantly increased under orbital shear, though less than Glu683Cys (Figure 3C), confirming Leu694's positioning near the membrane‐water interface. We then assessed the effects of 2–07Ab and 3–17Ab on shear‐induced TMD embedding. Contrary to our expectation, 2–07Ab did not significantly reduce the level of force‐induced biotinylation of the Leu694Cys mutant compared to that observed with the non‐specific antibody control (Figure 3D,E). In contrast, treatment with 3–17Ab significantly inhibited force‐dependent biotinylation (Figure 3F,G), suggesting that its efficacy may result from direct suppression of TMD movement. Notably, abciximab, which targets the fibrinogen‐binding site, did not inhibit biotinylation (Figure 3G). These findings indicate that 3–17Ab prevents the exposure of Leu694 to the aqueous environment, a topological change in the β3 TMD previously observed under mechanical force [25], which may underlie its ability to inhibit force‐induced integrin αIIbβ3 activation. The absence of a similar effect with 2–07Ab may be attributed to differences in binding mode, such as recognition of an epitope that does not directly affect Leu694 exposure but instead constrains force‐induced structural rearrangements of the β3 transmembrane domain that are not captured by the Leu694 reporter. Based on these results, 3–17Ab was selected for further investigation because its effect on force‐induced β3 topology could be more directly evaluated in the context of our hypothesis.

### 2.4 Mode of Action of 3–17Ab

**FIGURE 4:**
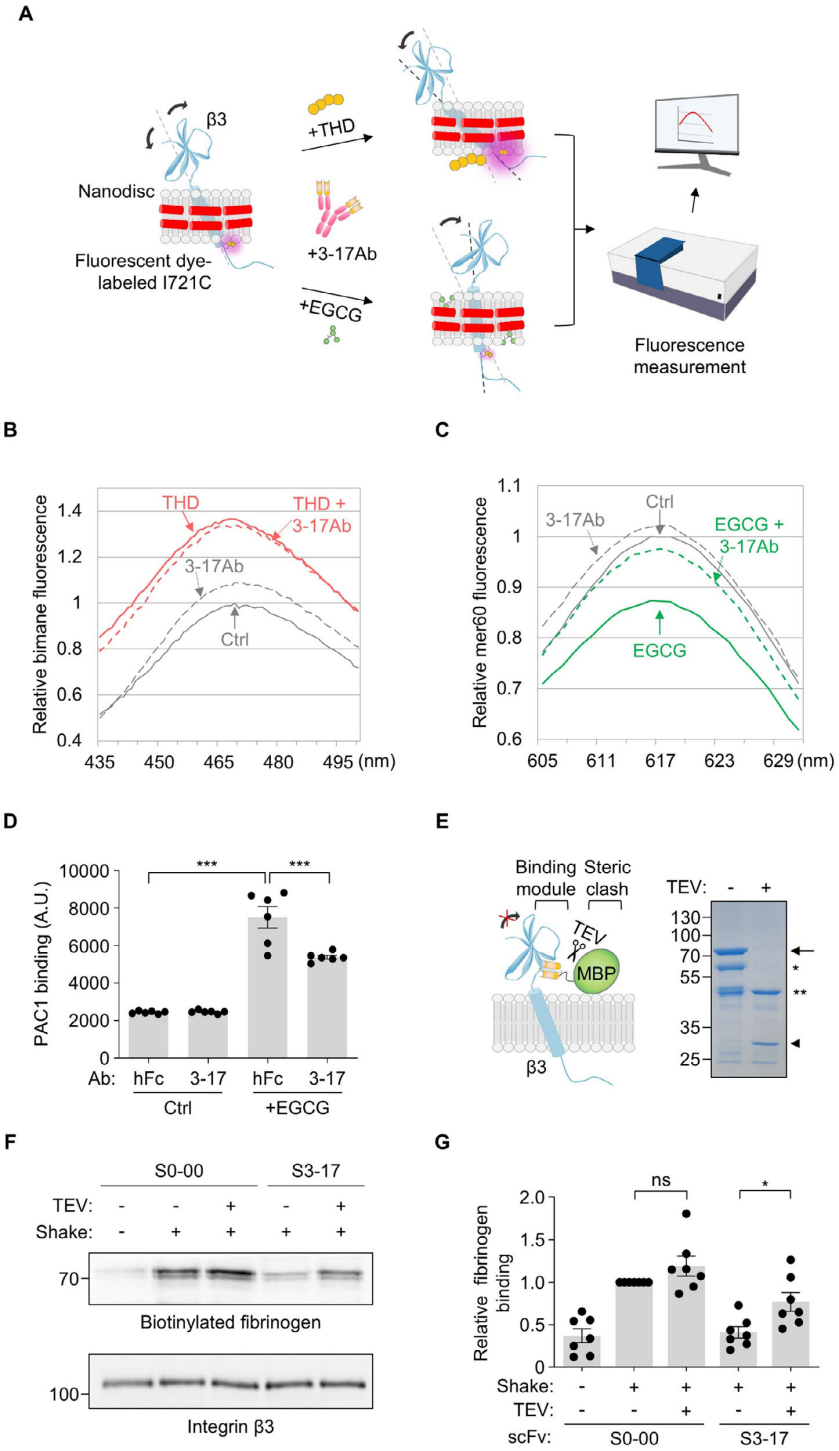
Effects of 3–17Ab on integrin β3 TMD embedding in the lipid bilayer. (A) Schematic diagram of fluorescence‐based assay using βTD‐TMD‐tail (Ile721Cys) embedded in nanodiscs. (B) Fluorescence emission spectra of bimane‐labeled βTD‐TMD‐tail (Ile721Cys) nanodiscs in the absence or presence of 10 µm THD with 1 µm control Fc (solid line) or 3–17Ab (dashed line). Mean fluorescence from three measurements was normalized to the peak intensity of the hFc control group. (C) Fluorescence emission spectra of mero60‐labeled βTD‐TMD‐tail (Ile721Cys) nanodiscs in the absence or presence of 100 µm EGCG with 1 µm control Fc (solid line) or 3–17Ab (dashed line). (D) CHO/αIIbβ3 cells were treated with 100 µm EGCG and 1 µm of each antibody for 30 min. PAC1 binding to cells was measured using flow cytometry, and the mean PAC1 fluorescence is shown as a bar graph (*n* = 6). Error bars indicate the mean ± SEM. ∗∗∗, *p* < 0.001 (one‐way ANOVA using Tukey's multiple comparison test). (E) Illustration of the interaction between integrin β3 βTD and scFv fused with MBP (left). Coomassie‐stained SDS‐PAGE of scFv before and after TEV cleavage is shown (right). The arrow and arrowhead indicate full‐length and MBP‐cleaved forms of scFv, respectively (right). The bands indicated with the single (*) and double asterisk (**) are contaminants, presumably MBP‐V_H_ and MBP, respectively. (F, G) CHO/αIIbβ3 cells were treated with 1 µm intact or cleaved forms of scFv under orbital shaking, and fibrinogen binding was analyzed as described in Figure 2F (*n* = 7). Error bars indicate the mean ± SEM. ∗, *p* < 0.05; ns, not significant (one‐way ANOVA using Tukey's multiple comparison test).

We previously demonstrated that environment‐sensitive fluorescent dyes conjugated to either the N‐ or C‐terminal regions of the TMD, specifically at Leu694 or Ile721 located at the membrane–water interface, can detect local hydrophobicity changes and thus serve as indicators of TMD embedding within artificial lipid bilayer nanodiscs [25, 26]. To further elucidate the mode of action of 3–17Ab, we applied this fluorescence‐based approach using a βTD–TMD–tail peptide construct (Figure 4A). Given that 3–17Ab binds near Leu694, we selected Ile721 for cysteine substitution and dye conjugation labeling to avoid direct interference with the epitope. As previously reported, the addition of the purified THD increased fluorescence from bimane‐conjugated βTD–TMD–tail (I721C) peptides, indicating a shift to a more hydrophobic environment consistent with increased TMD tilting (Figure 4B). Co‐treatment with 3–17Ab had minimal effect on this talin‐induced fluorescence change (Figure 4B), suggesting that 3–17Ab does not interfere with talin‐mediated TMD embedding. We next examined its effect on force‐induced TMD movement. As mechanical force cannot be directly applied to lipid bilayers in nanodiscs, we instead used epigallocatechin gallate (EGCG), which mimics force‐induced TMD tilting by decreasing the tilt angle and activating integrin αIIbβ3 [33]. Due to spectral overlap between EGCG and bimane, we used the alternative dye mero60 [34] to label the cysteine‐substituted Ile721 residue. Treatment with EGCG led to a decrease in fluorescence, indicating reduced TMD tilting; this effect was largely prevented by 3–17Ab (Figure 4C), suggesting that 3–17Ab restricts TMD movement toward a less tilted state. Consistent with the in vitro observation, 3–17Ab significantly suppressed EGCG‐induced activation of integrin αIIbβ3 in CHO/αIIbβ3 cells, as measured by PAC1 binding via flow cytometry (Figure 4D), further supporting its inhibitory effect on TMD tilting. To test our original hypothesis that steric hindrance contributes to the inhibitory mechanism of the βTD‐binding antibody, we prepared the scFv form of 3–17Ab (S3‐17), which includes a bulky MBP tag, and then cleaved it using tobacco etch virus (TEV) protease (Figure 4E). Cleavage of the MBP domain resulted in a partial but significant reduction in the scFv's inhibitory effect on orbital shaking–induced integrin activation (Figures 4F,G), consistent with a possible contribution of steric hindrance to the mode of action.

### 2.5 Ex Vivo Efficacy and Safety of 3–17Ab

**FIGURE 5:**
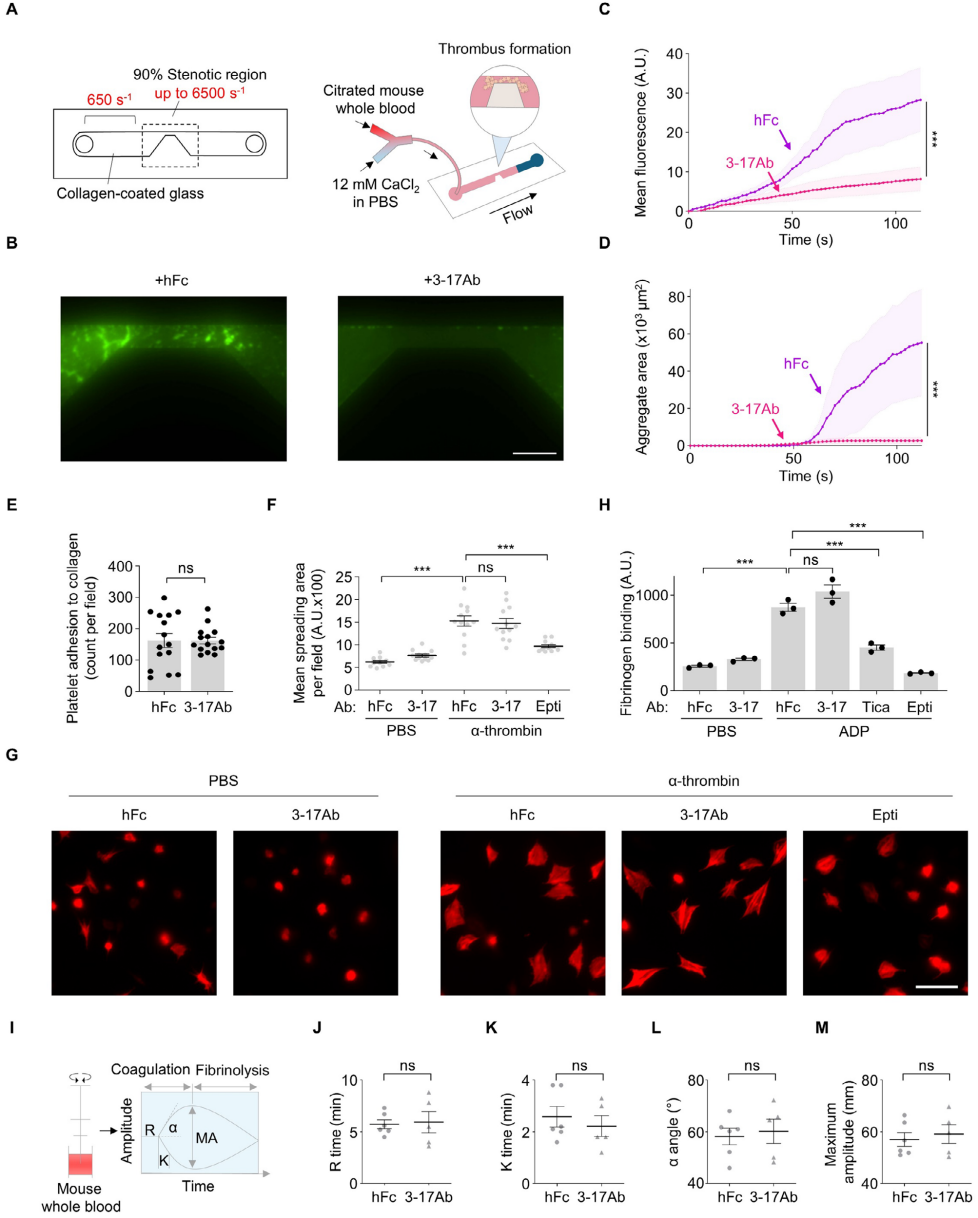
Effects of 3–17Ab on ex vivo platelet functions. (A) Schematic diagram of stenotic flow chamber setup (left) and mouse whole blood perfusion assay (right). (B) Representative fluorescence images of the stenotic region (112 s) following perfusion in the presence of either hFc or 3–17Ab. Platelets were incubated with 1 µm antibody together with 1 µm DiOC_6_. Scale bar, 200 µm. (C,D) Quantification of the mean fluorescence intensity (C) and total area of platelet aggregates (D) in the stenotic region over time during perfusion (*n* = 5). Shaded areas represent mean ± SEM. ∗∗∗, *p* < 0.001 (repeated measures two‐way ANOVA). (E) Mouse platelets were incubated on a collagen‐coated surface in the presence of 1 µm antibody. Adherent platelets were fixed, stained with rhodamine‐phalloidin, and counted (*n* = 15 microscope fields from three independent experiments). Error bars indicate the mean ± SEM. ns, not significant (unpaired t‐test). (F) Mouse platelets were pretreated with 1 U/mL of α‐thrombin (or PBS), together with either 1 µm antibody or 100 µm eptifibatide, and incubated on a fibrinogen‐coated surface for 30 min. Adherent platelets were fixed and stained with rhodamine‐phalloidin, and the average platelet spreading area in each microscopic field (50–100 platelets/field) was calculated and displayed as dots (*n* = 12 fields from three independent replicates). Error bars indicate the mean ± SEM. ∗∗∗, *p* < 0.001; ns, not significant (one‐way ANOVA using Tukey's multiple comparison test). (G) Representative images from (F) are shown. Scale bar, 10 µm. (H) Mouse platelets were pretreated with 10 µm ADP together with 1 µm hFc, 1 µm 3–17Ab, 1 µm ticagrelor, or 10 µm eptifibatide. Platelets were then incubated with fluorescein isothiocyanate (FITC)‐labeled fibrinogen for 30 min at room temperature. Fibrinogen binding was measured using flow cytometry and shown as bar graphs (*n* = 3). Error bars indicate the mean ± SEM. ∗∗∗, *p* < 0.001; ns, not significant (one‐way ANOVA using Tukey's multiple comparison test).

To evaluate the physiological relevance of 3–17Ab, a microfluidic chamber mimicking atherosclerotic stenosis was adopted [35]. Platelets in citrated whole blood extracted from mice were pre‐stained with 3,3‐dihexyloxacarbocyanine iodide (DiOC_6_) [36], mixed 1:1 with 12 mm CaCl_2_ solution for recalcification [37], and injected into the chamber, which contained a stenotic region in the middle (Figure 5A). Accumulation of platelets at the stenotic region was monitored under a fluorescence microscope and recorded for 2 min (Figure 5B; Video S1). During the procedure, fluorescence intensity at the stenotic region gradually increased, which appeared to result from discrete particles—possibly indicating platelet aggregation—as well as background fluorescence suggestive of platelet adhesion to the collagen‐coated surface (Figure 5B). When the mean fluorescence intensity in the region over time (reflecting both platelet adhesion and aggregation), as well as the total area of particles larger than 30 µm^2^ over time (reflecting platelet aggregation), were analyzed (Figure 5C,D), we found that treatment with 1 µm 3–17Ab significantly reduced both values, indicating that 3–17Ab can suppress pathological platelet aggregation. Based on the increased background fluorescence in the presence of 3–17Ab (Figure 5C, pink dots), we also note that platelet adhesion does not appear to be affected by 3–17Ab. When the assay was performed using heparinized whole blood under anticoagulated conditions, platelet aggregation, quantified as the total area of particles larger than 30 µm^2^, was similarly inhibited, whereas platelet adhesion, reflected by the mean fluorescence intensity in the region, remained preserved (Figure S9). Consistently, platelet adhesion to the collagen‐coated surface under static conditions for 30 min was not inhibited by 3–17Ab (Figure 5E). These results suggest that 3–17Ab inhibits thrombus formation mediated by integrin αIIbβ3 at the stenosis site, without interfering with platelet adhesion itself, which is likely mediated by collagen receptors—consistent with previous reports [23].

Next, we tested the potential adverse effects of 3–17Ab using mouse platelets ex vivo. Platelet spreading on fibrinogen‐coated surfaces, induced by α‐thrombin under static conditions, was not affected by 3–17Ab, whereas eptifibatide significantly inhibited spreading (Figure 5F,G). Similarly, 3–17Ab did not impair soluble fibrinogen binding induced by another agonist, ADP, as measured by flow cytometry (Figure 5H). In contrast, ticagrelor, an ADP receptor antagonist, and eptifibatide, an integrin αIIbβ3 antagonist, significantly inhibited ADP‐induced fibrinogen binding (Figure 5H). Consistently, ADP‐induced platelet spreading on fibrinogen‐coated surfaces showed similar spreading kinetics and stage distribution in the presence or absence of 3–17Ab (Figure S10). Furthermore, we examined the effect of 3–17Ab on agonist‐induced platelet activation using the mouse integrin αIIbβ3 activation‐specific antibody JON/A [38]. The increased JON/A binding observed in mouse platelets stimulated with α‐thrombin, ADP, or collagen was not altered by 3–17Ab (Figure S11A–C). Likewise, light transmission aggregometry of platelet‐rich plasma showed no significant differences in agonist‐induced aggregation in the presence of 3–17Ab (Figure S11D–F). These findings suggest that 3–17Ab does not impair normal hemostasis and is likely safe in terms of bleeding risk.

### 2.6 In Vivo Efficacy and Safety of 3–17Ab

**FIGURE 6:**
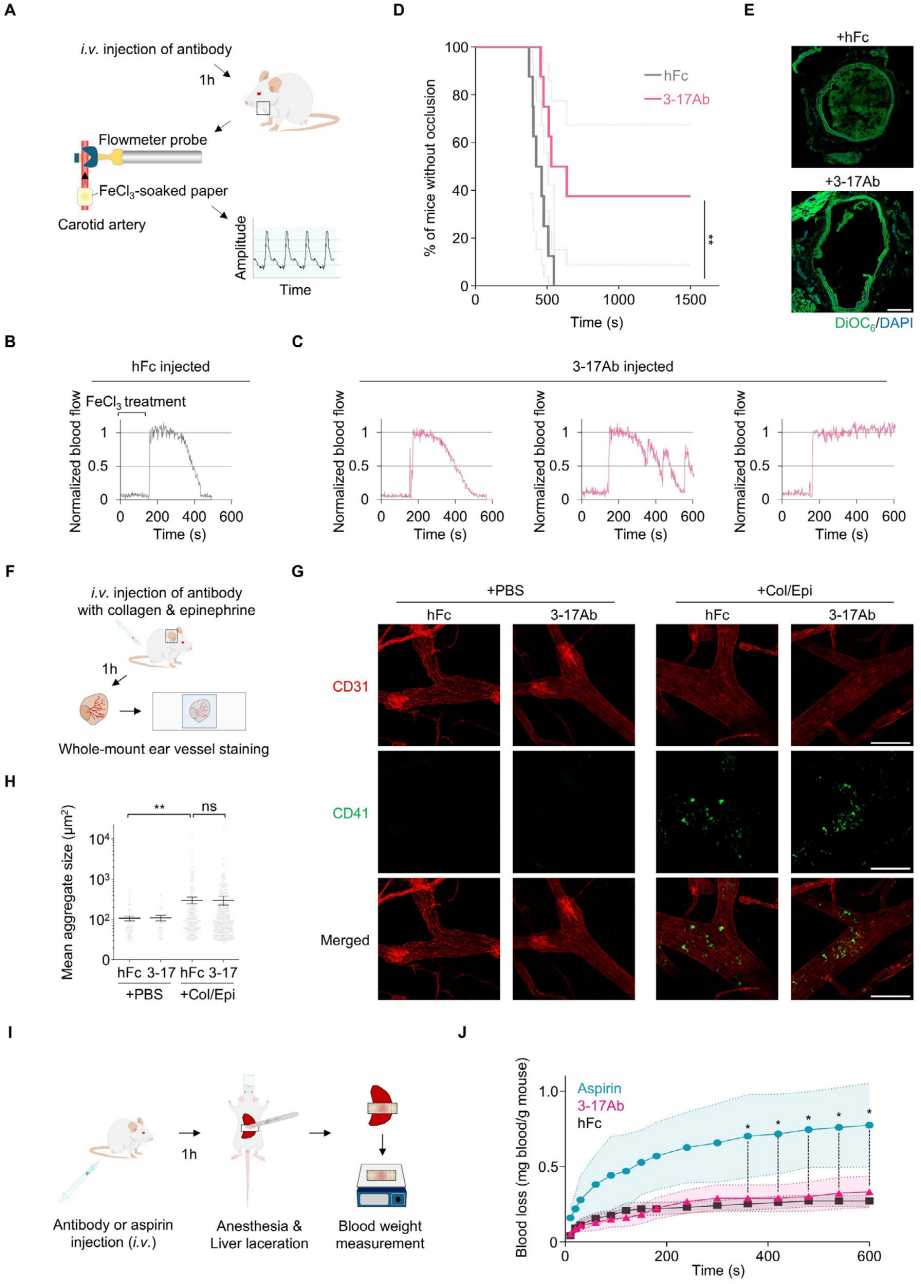
Effects of 3–17Ab on in vivo platelet functions. (A) Schematic illustration of FeCl_3_‐induced carotid artery thrombosis model. (B,C) Representative blood flow traces showing the average flow rate (1.7 s intervals) in the carotid artery after injection of 10 mg/kg hFc (B) or 3–17Ab (C). Signal amplitude was normalized to the average blood flow from the initial 10 sec after FeCl_3_ application. (D) Kaplan–Meier plot showing the proportion of mice maintaining normal blood flow over time. Occlusion was defined as a sustained drop in normalized blood flow < 0.1 for >30 s (*n* = 8). Dotted lines indicate 95% confidence intervals. ∗∗*p* < 0.01 using the log‐rank (Mantel–Cox) test. (E) Representative histological images of carotid artery sections from each group, with platelets visualized by intravenous injection of 0.05 µmol/kg DiOC_6_ prior to surgery. Scale bar, 100 µm. (F) Mice were intravenously injected with 0.275 mg/kg collagen and 1.2 µg epinephrine, together with 10 mg/kg control hFc or 3–17Ab, as indicated. One hour later, ears were harvested for whole‐mount immunostaining. (G) Z‐stack (1 µm) images stained for CD31 (endothelium, red) and CD41 (platelets, green) were merged using ImageJ average intensity projection. Representative images are shown. Scale bar, 100 µm. (H) Areas of platelet aggregates, defined as CD41‐positive particles larger than 30 µm^2^, were quantified from 18 fields centered on major vessels in four mice and plotted as individual data points. Error bars indicate the mean ± SEM. ∗∗, *p* < 0.01; ns, not significant (one‐way ANOVA using Tukey's multiple comparison test). (I) Schematic overview of the liver laceration mouse model. Mice injected with 10 mg/kg antibody or 13.5 mg/kg aspirin were anesthetized and subjected to liver laceration using a scalpel. Blood loss was quantified by weighing blood‐soaked gauze at defined time points. (J) Cumulative blood loss over time, normalized with the mouse body weight, is shown (*n* = 4 for the 3–17Ab and hFc groups, and *n* = 3 for the aspirin group). Shaded areas indicate the mean ± SEM. ∗*p* < 0.05 (one‐way ANOVA using Tukey's multiple comparison test).

We next investigated the effect of the 3–17Ab in a FeCl_3_‐induced carotid artery thrombosis mouse model, a well‐established approach for inducing thrombus formation [39]. FeCl_3_‐soaked filter papers were applied to the carotid artery of mice injected with either control human IgG Fc fragment (hFc) or 3–17 Ab, and blood flow was monitored using a perivascular probe connected to a flowmeter (Figure 6A). In control hFc‐injected mice, blood flow ceased within approximately 600 s after FeCl_3_ application (Figure 6B; Figure S12A). In contrast, mice injected with 3–17Ab exhibited delayed occlusion (Figure 6C, left; Figure S12B–D), repeated cycles of occlusion and reperfusion prior to final occlusion (Figure 6C, middle), or complete prevention of occlusion (Figure 6C, right). When the data were pooled and presented as a Kaplan–Meier plot showing the proportion of mice maintaining blood flow over time, 3–17Ab significantly delayed occlusion more than that observed in mice injected with control hFc (Figure 6D). Histological analysis of carotid artery cross‐sections confirmed thrombus formation within the arterial lumen of hFc‐injected mice, whereas no thrombus was observed in sections from non‐occlusive 3–17Ab‐injected mice (Figure 6E). As an alternative approach to confirm in vivo efficacy, we also employed a mouse model of mechanical carotid artery injury in which repeated forceps clamping followed by stenosis was applied (Figure S13A). In the control group, stenosis of the forceps‐clamped vessel resulted in gradual occlusion in most cases, as indicated by a reduction in blood flow within approximately 100 s. In contrast, following 3–17Ab injection, occlusion was significantly delayed, and in most animals did not occur within the maximum observation period of 8 min (Figure S13B–E). These findings demonstrate that inhibition of mechanical force–dependent integrin activation is an effective strategy for suppressing thrombosis.

Next, we investigated the potential in vivo effects of 3–17Ab on agonist‐driven platelet aggregation. To induce systemic platelet aggregation independent of shear stress, we intravenously injected a sublethal dose of collagen/epinephrine mixture, as previously described [40, 41], and detected platelet aggregates by staining whole‐mount mouse ear tissue (Figure 6F). Injection of the mixture caused prominent platelet aggregation, as indicated by staining with a platelet‐specific anti‐CD41 (integrin αIIb) antibody within blood vessels, regardless of 3–17Ab administration (Figure 6G). When CD41‐positive regions larger than 30 µm^2^—used as indicators of platelet aggregates— were selected (Figure S14) and analyzed, no significant reduction in the mean size of these regions was observed in the 3–17Ab‐treated group compared to those in the control group (Figure 6H), suggesting that 3–17Ab does not interfere with agonist‐dependent platelet aggregation. To directly assess potential adverse effects on physiological hemostasis, we employed the tail bleeding assay [42, 43]. Administration of 3–17Ab did not significantly prolong bleeding time compared with the Fc control group, despite occasional prolonged bleeding events. However, as reported in previous studies [44, 45], aspirin treatment markedly increased bleeding time, with most animals reaching the predefined observation limit of 1200 s (Figure S15A,B). To further assess potential bleeding risk, we examined the effect of 3–17Ab using a liver laceration mouse model [46]. Mice were administered control hFc, 3–17Ab, or aspirin, followed by anesthesia, liver laceration, and quantification of blood loss (Figure 6I). Consistently, aspirin caused greater blood loss than that in the hFc control group (Figure 6J), whereas 3–17Ab‐treated mice showed a similar amount of bleeding to that observed in the control group. In addition, administration of 3–17Ab did not induce any detectable gut bleeding in fecal occult blood test (Figure S15C), nor did it cause abnormal blood cell counts (Figure S15D–F). These findings indicate that 3–17Ab has a favorable safety profile with respect to bleeding risk, particularly when compared to aspirin. Taken together, these results demonstrate that 3–17Ab, designed to inhibit force‐dependent TMD movement, can suppress thrombosis without inducing bleeding complications, highlighting its potential as a safe therapeutic agent.

## 3 Discussion

Recent studies suggest that abnormally increased blood flow at the stenosis site, rather than chemical stimuli, is primarily responsible for pathological thrombus formation [21, 23, 47, 48]. When blood flow accelerates through atherosclerotic lesions, elastic or polymeric molecules are subjected to elongational forces [49], which induce the extension of oligomeric von Willebrand factor (vWF) from its native, coiled conformation [50]. This force‐driven structural change exposes multiple collagen‐ and platelet‐binding sites along the extended vWF oligomer, thereby increasing its avidity for both the vascular lesion and circulating platelets and promoting efficient platelet recruitment. This provides a mechanistic explanation for rheology‐dependent platelet adhesion.

We previously demonstrated that mechanical forces stretching the plasma membrane induce a topological change in the integrin β3 TMD, weakening the αIIb–β3 TMD interaction and activating the integrin independently of intracellular signaling [25]. We propose that this force‐dependent activation mechanism may explain how thrombosis forms in a rheology‐dependent manner at sites of arterial stenosis. Specifically, once platelets adhere to vWF at the site of stenosis, they are exposed to shear forces that directly activate integrin αIIbβ3 and promote fibrinogen binding on their surface. In parallel, elongational forces stretch circulating platelets, priming αIIbβ3 to engage fibrinogen presented by adherent platelets. We speculate that shear forces on adherent platelets and elongational forces on circulating platelets, both leading to integrin αIIbβ3 activation, may be responsible for the rheology‐dependent platelet aggregation at atherosclerotic lesions. Consistent with this model, blockade of the integrin αIIbβ3–fibrinogen interaction significantly reduces platelet aggregation at such sites [23], suggesting that mechanically induced αIIbβ3 activation could serve as a viable therapeutic target for preventing pathological thrombosis.

In this study, we demonstrate that targeting force‐induced changes in integrin β3 TMD embedding can selectively inhibit the force‐dependent pathological activation of integrin αIIbβ3. Based on our previous findings that mechanical and chemical stimuli induce distinct topological movements of the β3 TMD [25, 26], we hypothesized that βTD‐targeting antibodies could sterically block force‐specific activation. Indeed, several scFv clones binding to this region inhibited force‐dependent integrin activation (Figure 2A; Figure S3). One such clone, S3‐17, was selected, and its steric hindrance capacity (Figure 4F), ability to restrict force‐specific β3 TMD movement (Figures 3 and 4C), and efficacy without adverse effects (Figures 5 and 6) were demonstrated, validating the feasibility of this approach. Collectively, these findings support the concept that targeting β3 TMD topology offers a novel strategy for selectively modulating integrin function. Although we did not conduct detailed mechanistic studies for all clones, further investigation may yield additional valuable insights into the precise epitope specificity and spatial orientation requirements of βTD‐targeting antibodies, thereby advancing this approach toward the development of next‐generation antiplatelet drugs.

The current use of integrin αIIbβ3 antagonists is largely restricted to patients with ACS or those undergoing PCI [51]. However, peri‐procedural mortality in these settings is often driven by bleeding complications [52], and existing antagonists carry a substantial bleeding risk. In this context, 3–17Ab, which significantly inhibited thrombosis in an acute model with minimal bleeding risk (Figure 6), may represent a safer alternative therapeutic option for patients with ACS or undergoing PCI. Owing to its mechanism‐based safety profile, 3–17Ab could be broadly applicable in clinical situations where thrombotic risk is elevated, but bleeding concerns limit the use of conventional antiplatelet drugs. For example, patients undergoing surgery, where antiplatelet agents are often withheld, may benefit from this approach. Similarly, individuals requiring circulatory support devices such as extracorporeal membrane oxygenation (ECMO), left ventricular assist devices, or mechanical heart valves—conditions in which high shear stress activates platelets and promotes thrombosis [53, 54, 55, 56, 57]—could also benefit from such a strategy.

Potential cross‐reactivity with endothelial integrin αvβ3 should be considered as a limitation of the current study. Endothelial αvβ3 is abundantly expressed but is primarily involved in cell migration and angiogenesis [58]. Previous studies have also shown that mechanical activation of endothelial αvβ3 occurs predominantly at the basal (abluminal) surface rather than the apical (luminal) surface [59]. Based on these considerations, intravenously injected 3–17Ab is expected to have minimal impact on endothelial αvβ3 under physiological conditions. Nonetheless, future studies will be required to directly evaluate integrin selectivity and endothelial effects of 3–17Ab.

In summary, this study demonstrates that targeting the βTD to restrict force‐dependent β3 TMD movement selectively suppresses pathological integrin αIIbβ3 activation without impairing physiological hemostasis. While this study establishes a proof‐of‐concept for TMD topology‐selective inhibition of integrin αIIbβ3, further optimization of the antibody may enhance efficacy and specificity. This mechanistically targeted approach holds promise as a safer antithrombotic therapy for use in both interventional and high‐risk clinical settings.

## 4 Experimental Section/Methods

### 4.1 Plasmids

To generate GST–βTD fusion proteins, the human integrin β3 (residues 636–692) or integrin β1 (residues 646–703) coding regions were amplified by polymerase chain reaction (PCR) and cloned into the pGEX‐4T1 vector, with a C‐terminal 6xHis tag added for purification. For the 6xHis–ketosteroid isomerase (KSI)–βTD–β3‐tail construct, the βTD–β3‐tail region was PCR‐amplified and inserted into the pET31 vector containing 6xHis and KSI [26]. To generate MBP–scFv–His–HA, the scFv–His–HA regions were cloned into the pMAL‐c2x vector. The variable regions of the scFv were further inserted into the heavy and light chain constructs of IGN‐002 [31] using EcoRI/XhoI restriction sites. All mutagenesis was performed using PCR‐mediated site‐directed mutagenesis.

### 4.2 Antibody Purification

For antibody purification, FreeStyle 293‐F cells (Thermo Fisher Scientific) were transfected with plasmids encoding the heavy and light chains of each clone using linear polyethylenimine (Polysciences). The cells were cultured in FreeStyle 293 expression medium (Thermo Fisher Scientific) at 37°C in suspension, supplemented with 0.5% tryptone (Sigma) 24 h post‐transfection. Seven days after transfection, the antibodies secreted into the medium were purified using a HiTrap MabSelect SuRe column (Cytiva).

### 4.3 scFv Displaying‐Phage Library Screening

To screen for scFvs binding specifically to the human βTD, 1 µg of purified human βTD was coated onto a 96‐well strip immunoplate (SPL) and blocked with 3% bovine serum albumin (BSA) in Tris‐buffered saline (TBS; 50 mm Tris‐HCl, 150 mm NaCl, pH 7.4). The phage library was added and incubated for 1 h in the presence of 300 µg/mL of GST–β1 βTD as a competitor. Wells were washed with TBS containing 0.5% Tween‐20 to remove non‐specific binders. For the cell‐based panning process, phages were precleared with CHO cells (negative selection) and then incubated with CHO/αIIbβ3 cells. Bound phages were eluted and amplified via infection of *E. coli* ER2738 (Lucigen), followed by overnight incubation at 30°C with VCSM13 helper phage. Phages were then precipitated from the culture supernatants using PEG‐8000/NaCl, resuspended in TBS with 1% BSA, and used for a subsequent round of panning. Input and output phage titers were determined to monitor enrichment efficiency.

### 4.4 Orbital Shaking Experiments

CHO/αIIbβ3 cells cultured in 12‐well plates were washed with Hank's Balanced Salt Solution (HBSS; Welgene) and incubated in HBSS containing the indicated scFv or antibody, along with 20 µg/mL biotinylated fibrinogen. The plates were placed in a shaking CO_2_ incubator and rotated at 200 rpm for 20 min. After incubation, cells were washed and harvested for Western blot analysis. The inhibitory effects of clones were normalized to that of the negative control scFv (S0‐00) within the same membrane to ensure consistent quantification across different blots. To label exposed cysteines on integrin β3, the same procedure was followed, except that EZ‐Link Maleimide‐PEG_2_‐Biotin (Thermo Fisher Scientific), a membrane‐impermeable biotinylation reagent, was used. Following incubation, cells were quenched and washed with phosphate‐buffered saline (PBS) containing N‐acetyl cysteine (Sigma), then lysed and incubated with streptavidin agarose beads. The bead‐bound integrin β3 was subsequently analyzed by Western blotting.

### 4.5 Flow Cytometry

For the scFv binding test, CHO or CHO/αIIbβ3 cells were incubated with 0.5 µm of each purified MBP‐scFv‐His‐HA clone and detected using an anti‐HA antibody. Integrin αIIbβ3 expression was detected using D57. The binding of antibodies to cells or platelets was analyzed using an Alexa Fluor 647‐conjugated anti‐human Fc antibody. For the integrin αIIbβ3 activation assay in CHO/αIIbβ3 cells, cells were incubated with 4 µg/mL of PAC1 (BD Biosciences) or with 80 µg/mL of fluorescein isothiocyanate (FITC)‐labeled fibrinogen.

### 4.6 Synthesis of mero60

**Mero60** was synthesized as described in Figure S16, using a modification of our earlier published procedure [60]. 2,3,3‐trimethyl‐1‐(3‐((3‐sulfopropyl)amino)propyl)indolinium bromide (**S1**) and (E)‐2‐(3‐methoxyallylidene)‐1H‐indene‐1,3(2H)‐dione (**S2**) were prepared using published procedures [34]. All operations with dyes were performed under dim light. NMR spectra were taken on a Bruker‐500 spectrometer using deuterated solvents purchased from Cambridge Isotope Laboratories. Reference peaks of 7.26 ppm (CDCl_3_), 2.50 ppm, and (DMSO‐d6) were used for ^1^H NMR spectra. High‐resolution mass spectra were obtained on a Thermo Scientific Q Exactive HF‐X mass spectrometer with direct infusion.

#### 4.6.1 3‐((tert‐Butoxycarbonyl)(3‐((E)‐2‐((E)‐4‐(1,3‐dioxo‐1,3‐dihydro‐2H‐inden‐2‐ylidene)but‐2‐en‐1‐ylidene)‐3,3‐dimethylindolin‐1‐yl)propyl)amino)propane‐1‐sulfonic acid (S3)

2,3,3‐Trimethyl‐1‐(3‐((3‐sulfopropyl)amino)propyl)indolinium bromide **S1** (0.420 g, 1.00 mmol), and di‐tert‐butyl dicarbonate (1.09 g, 5.00 mmol) were mixed with water (0.10 mL). The resulting mixture was sonicated for a minute and then heated at 65°C under reduced pressure in a rotary evaporator for 30 min. The residue obtained was mixed with (E)‐2‐(3‐methoxyallylidene)‐1H‐indene‐1,3(2H)‐dione **S2** (0.107 g, 0.50 mmol), sodium acetate (0.082 g, 1.00 mmol), and methanol (5.0 mL) under argon. The reaction mixture was stirred at 85°C under argon for 30 min. The solvent was removed under reduced pressure. The residue was purified using flash chromatography with methanol/dichloromethane mixture (1:9) as eluent. The product was isolated as a dark blue solid (207 mg, 55% yield). E/Z isomerism in tertiary amide caused peak doubling in ^1^H NMR analysis. ^1^H NMR (DMSO‐d6, 500 MHz) δ ppm 8.12 (t, *J* = 13.0 Hz, 1H), 7.77 – 7.69 (m, 5H), 7.55 (d, *J* = 13.0 Hz, 1H), 7.50 (d, *J* = 7.5 Hz, 1H), 7.34 (t, *J* = 8.5 Hz, 1H), 7.20 (d, *J* = 8.5 Hz, 1H), 7.13 (t, *J* = 7.5 Hz, 1H), 6.19 (d, *J* = 13.5 Hz, 1H), 3.99 (br s, 2H), 3.17‐3.27 (br m, 4H), 2.37 (t, *J* = 8.0 Hz, 2H), 1.88 (br s, 2H), 1.78 (br s, 2H), 1.64 (s, 6H), 1.40 & 1.29 (two s, 9H)); HRMS (negative mode) obsd 619.2492, calcd 619.2483 [(M)^−^, M = C_34_H_39_N_2_O_7_S^−^].

#### 4.6.2 Mero60

A solution of compound **S3** (65 mg, 0.087 mmol) in dichloromethane (3.0 mL) was treated with trifluoroacetic acid (1.0 mL) and stirred under argon for 2 h. Completion of Boc deprotection was confirmed by TLC analysis. Then the reaction mixture was concentrated under reduced pressure, and the residue was passed through a short silica pad using a dichloromethane/methanol (93:7) mixture. The resulting residue was mixed with iodoacetic anhydride (55 mg, 0.15 mmol), THF (5.0 mL), and N,N‐diisopropylethyl amine (0.11 mmol, 0.020 mL) and stirred under argon for 8 h. Hexane/ethyl acetate/diethyl ether mixture (1:1:1, 100 mL) was then added. The resulting precipitate was purified by flash chromatography using methanol/dichloromethane mixture (3:17) as eluent. The product was isolated as a dark blue solid (54 mg, 76% yield). E/Z isomerism in tertiary amide caused peak doubling in ^1^H NMR analysis. ^1^H NMR (CDCl_3_/CD_3_OD, 500 MHz) δ 7.78 (t, *J* = 13.0 Hz, 1H), 7.72 – 7.67 (m, 2H), 7.60 – 7.49 (m, 4H), 7.36 – 7.35 (m, 2H), 7.24 (t, *J* = 8.0 Hz, 1H), 7.06 (t, *J* = 7.5 Hz, 1H), 7.00 – 6.90 (m, 1H), 6.01 – 5.86 (m, 1H), 3.84 (s, 2H), 3.54 – 3.34 (m, 4H), 3.16 – 2.78 (m, 2H), 2.13 – 2.08 (m, 2H), 2.01 – 1.91 (m, 2H), 1.61 & 1.59 (two s, 6H); HRMS (negative mode) obsd 687.1034, calcd 687.1031 [(M)^−^, M = C_31_H_32_IN_2_O_6_S^−^].

### 4.7 Nanodisc Experiment

Preparation of the integrin β3 nanodisc was performed as previously described [25, 26]. Briefly, β3 KSI–βTD–TMD–tail protein, labeled with bimane or mero60, was purified using Ni^2+^–nitrilotriacetic acid beads, followed by cleavage of KSI. The purified protein was dialyzed against TBS containing 0.1% Triton X‐100 and mixed with a 1:1 lipid mixture of 1,2‐dimyristoyl‐sn‐glycero‐3‐phosphocholine and 1,2‐dimyristoyl‐sn‐glycero‐3‐phospho‐(1ʹ‐rac‐glycerol) (Avanti Polar Lipids), along with purified membrane scaffold protein 1D1 (MSP1D1). SM‐2 Biobeads (Bio‐Rad) were added to initiate nanodisc assembly. The resulting nanodiscs were incubated with 10 µm THD or 100 µm EGCG in the presence of 1 µm control hFc or 3–17Ab for 20 min. Bimane and mero60 fluorescence were measured using a FluoroMax‐2 spectrofluorometer (HORIBA Scientific) with excitation at 370 nm and emission scanning from 420 to 500 nm for bimane, or excitation at 593 nm and emission scanning from 605 to 655 nm for mero60.

### 4.8 Ethics Approval Statement

All animal procedures were approved by Korea University Institutional Animal Care and Use Committee (KUIACUC, approval numbers 2023‐0052 and 2024‐0086). Eight‐week‐old BALB/c male mice were purchased from Raonbio Inc. (Yongin, Republic of Korea) and Orient Bio Inc. (Seongnam, Republic of Korea). To assess safety regarding treatment‐induced weight loss, we conducted a confirmatory in vivo study; we confirmed that animal body weight loss did not exceed 15% of the original body weight at any time during the study (Figure S17).

### 4.9 Platelet Isolation

For platelet isolation, the blood was drawn from cardiac puncture into 150 µL of ACD solution (85 mm sodium citrate, 65 mm citric acid, and 104 mm D‐glucose, pH 4.4) followed by addition of 875 µL Modified Tyrode's solution (140 mm NaCl, 2.7 mm KCl, 0.4 mm NaH_2_PO_4_, 10 mm NaHCO_3_, 5 mm dextrose, and 10 mm HEPES, pH 7.4) and 175 uL of ACD solution. After centrifugation at 200 g for 5 min at RT without brake, the platelet‐rich plasma (PRP) from the supernatant was collected and centrifuged at 700 g for 5 min at RT without brake. The resulting pellet was resuspended with Walsh buffer (137 mM NaCl, 2.7 mm KCl, 1 mm MgCl_2_, 3.3 mm NaH_2_PO_4_, and 20 mm HEPES, pH 7.4).

### 4.10 In Vitro Platelet Assays

Platelet adhesion or spreading assays were conducted essentially as previously described [25, 61]. Briefly, platelets resuspended in Walsh buffer were adhered to a collagen‐ or fibrinogen‐coated surface. After adhesion at 37°C for the indicated time, unbound platelets were washed, fixed with 3.7% paraformaldehyde, and stained with rhodamine‐phalloidin before imaging with fluorescence microscopy. For the mouse platelet integrin αIIbβ3 activation assay, agonist‐treated platelets were stained with a phycoerythrin (PE)‐conjugated JON/A antibody (Emfret Analytics) following the manufacturer's protocol, and analyzed by flow cytometry.

### 4.11 Stenotic Microfluidics Platelet Aggregation Assay

A polydimethylsiloxane microfluidic channel with a stenosis [35] was coated with 50 µg/mL collagen type I and blocked with 2% bovine serum albumin. Citrated blood samples were prepared by adding a 1:9 volume of buffered trisodium citrate solution (BD Biosciences) to whole blood obtained by cardiac puncture. For, heparinized blood sample, a final concentration of 20 USP units/mL of heparin (Sigma) was added to the whole blood. Citrated or heparinized mouse whole blood, pre‐incubated with 1 µm DiOC_6_ (Invitrogen) and 1 µm hFc or 3–17Ab for 20 min, was perfused into the channel using a syringe pump (Revodix Inc.). In case of citrated blood, 12 mm of CaCl_2_ was delivered via a Y‐shaped adaptor during perfusion for simultaneous recalcification. Images were acquired using a charge‐coupled device camera (DS‐Qi2, Nikon) and analyzed using NIS‐Elements AR (Nikon) and ImageJ (NIH) software.

### 4.12 FeCl_3_‐Induced Mouse Thrombosis Model

The FeCl_3_‐induced thrombosis model was performed following previously described protocols [62]. Briefly, mice intravenously injected with control hFc or 3–17Ab (10 mg/kg) were anesthetized, and the left common carotid artery was surgically exposed. A 1 mm × 1 mm filter paper soaked in 5% FeCl_3_ (Sigma) was placed on the artery for 2 min. A flow probe (MA‐0.5PSB) connected to a TS420 perivascular flowmeter module (Transonic Systems Inc.) was positioned around the artery to monitor blood flow. For histological analysis, mice injected with control hFc or 3–17Ab along with DiOC_6_ (0.05 µmol/kg) were sacrificed, and the dissected carotid arteries were fixed in 3.7% paraformaldehyde for 1 h, immersed in 30% sucrose overnight, embedded in optimal cutting temperature compound, and frozen on dry ice. Cryosections (10 µm) were mounted using Vectashield with 4′,6‐diamidino‐2‐phenylindole (DAPI) (Vector Laboratories) and imaged using a confocal microscope (LSM900, Zeiss) with Zeiss Zen software.

### 4.13 Mechanical Injury and Stenosis‐Induced Thrombosis Model

The mechanical injury and stenosis‐induced thrombosis model was performed with the following modifications to the Folts model [63]. 30 min after intravenous injection of control hFc or 3–17Ab (10 mg/kg), the exposed left carotid artery was pinched with fine forceps for 30 s, repeated five times at 30 s intervals. A 5‐0 silk suture was tied around the artery to generate 75% stenosis, which was monitored using a flowmeter. The flow rate was monitored for an additional 2 min after the initial occlusion was observed, or for a maximum of 8 min if no occlusion occurred.

### 4.14 Systemic Platelet Activation of Mouse and Whole Mount Ear Tissue Staining

Systemic activation of mouse platelets was induced by an intravenous injection of a sublethal dose of collagen (0.275 mg/kg; Sigma) and 1.2 µg of epinephrine [41] (Sigma), together with 10 mg/kg of control hFc or 3–17Ab. After 1 h, the mice were sacrificed, and the inner layer from the dissected ear was collected. After fixation in 3.7% paraformaldehyde in PBS for 1 h, the layer was washed with PBS three times and blocked with PBS containing 10% goat serum and 0.2% Triton X‐100. Immunostaining was performed by incubating with anti‐CD31 (Invitrogen) and anti‐CD41 (Biolegend) antibodies overnight at 4°C, followed by incubation with Alexa Fluor 488‐conjugated anti‐rat (Invitrogen) and Cy3‐conjugated anti‐Armenian hamster IgG antibodies (Jackson ImmunoResearch) for 1 h at RT. After washing, the layer was mounted on a glass slide and imaged using a confocal microscope (LSM900; Zeiss, Oberkochen, Germany) equipped with Zeiss Zen software.

### 4.15 In Vivo Hemostatic Evaluation

Liver and tail bleeding assays were performed 1 h after the intravenous injection of control hFc, 3–17Ab (10 mg/kg), or aspirin (13.5 mg/kg; Sigma). For the liver laceration assay, the mouse was anesthetized, and a 3‐mm horizontal cut was made on the left liver lobe using a surgical blade (#20). Cotton gauze was applied to the wound to absorb blood and weighed to quantify blood loss at each time point. The total accumulated blood weight was normalized to the body weight of the mouse. For the mouse tail bleeding assay, the tail of an anesthetized mouse was cut 3 mm from the tip. The bleeding time was measured by blotting onto filter paper every 30 s. The measurement was terminated if bleeding did not stop within 20 min to avoid excessive blood loss.

To measure fecal occult blood, hFc, 3–17Ab (10 mg/kg) or aspirin (13.5, 50, and 250 mg/kg) was intravenously administered. 24 h later, 20–30 mg of fecal samples were collected, lysed with a 0.53 m potassium hydroxide solution, and centrifuged. The resulting supernatant was mixed with 90 µL of luminol solution (0.53 m KOH, 1.5% H_2_O_2_, 0.04% luminol), and the chemiluminescence was measured using a spectrophotometer.

### 4.16 Statistical Analysis

Statistical analyses were performed using GraphPad Prism version 10 (GraphPad Software, La Jolla, CA, USA). Data are expressed as mean ± standard error of the mean (SEM), unless otherwise specified. Comparisons between experimental groups were conducted using one‐way analysis of variance (ANOVA), followed by Tukey's multiple comparisons test, unless otherwise stated. Statistical significance was set at *p* < 0.05.

## Author Contributions

J.L. and C.K. contributed to conceptualization. J.L., C.G.L., P.V., J.M.K., J.K., and K.M.H. contributed to methodology. J.L., J.M.K., J.K., J.Y.P., H.W.C., T.S.U., and C.K. contributed to formal analysis. J.L., C.G.L., P.V., J.M.K., and J.K. contributed to the investigation. H.S. contributed to the resources and supervision of the library screening. J.L. contributed to the writing of the original draft. J.Y.P., K.M.H., H.W.C., T.S.U., S.J.H., and C.K. contributed to writing, reviewing, and editing.

## Funding

Basic Science Research Program through the National Research Foundation of Korea (grant numbers RS‐2025‐00520706 and RS‐2023‐00221182). US National Institutes of Health (R35‐GM122596 and RM1GM145399 to KMH). A grant from Korea University.

## Conflicts of Interest

Some of the authors intend to file a patent application based on the findings reported in this manuscript.

## Supporting information




**Supporting File 1**: advs75275‐sup‐0001‐SuppMat.pdf.

**Supporting File 2**: advs75275‐sup‐0002‐Data.zip.

**Supporting File 3**: advs75275‐sup‐0003‐VideoS1.mp4.

## Data Availability

The data that support the findings of this study are available from the corresponding author upon reasonable request.
