# Supplementary material for: Selective Inhibition of Integrin β3 Topology Provides a Safer Antithrombotic Strategy
Source: Adv Sci (Weinh). 2026 Apr 13;13(39):e22086. doi: 10.1002/advs.202522086 (PMC13334950; doi:10.1002/advs.202522086)
Supplement: Supplementary file 1 — Supporting File 1: advs75275‐sup‐0001‐SuppMat.pdf. [file ADVS-13-e22086-s002.pdf]

Supporting Information

Selective Inhibition of Integrin β3 Topology Provides a Safer Antithrombotic Strategy

*Joonha Lee[1], Chul-Gyun Lim[1], Pothiappan Vairaprakash[2], Jong-Min Kim[3], Jiyoon Kim[4], Ji-Young Park[3], Klaus M. Hahn[2], Hyunbo Shim[5], Hae Woong Choi[1], Tobias S. Ulmer[6], Soon Jun Hong[7], Chungho Kim[*1]*

[*]Corresponding Author: Chungho Kim, Ph.D. Department of Life Sciences, Korea University, Seoul 136-701, Republic of Korea. E-mail: chungho@korea.ac.kr. Phone: +82-2-3290-3402.

Supplemental Figure 1

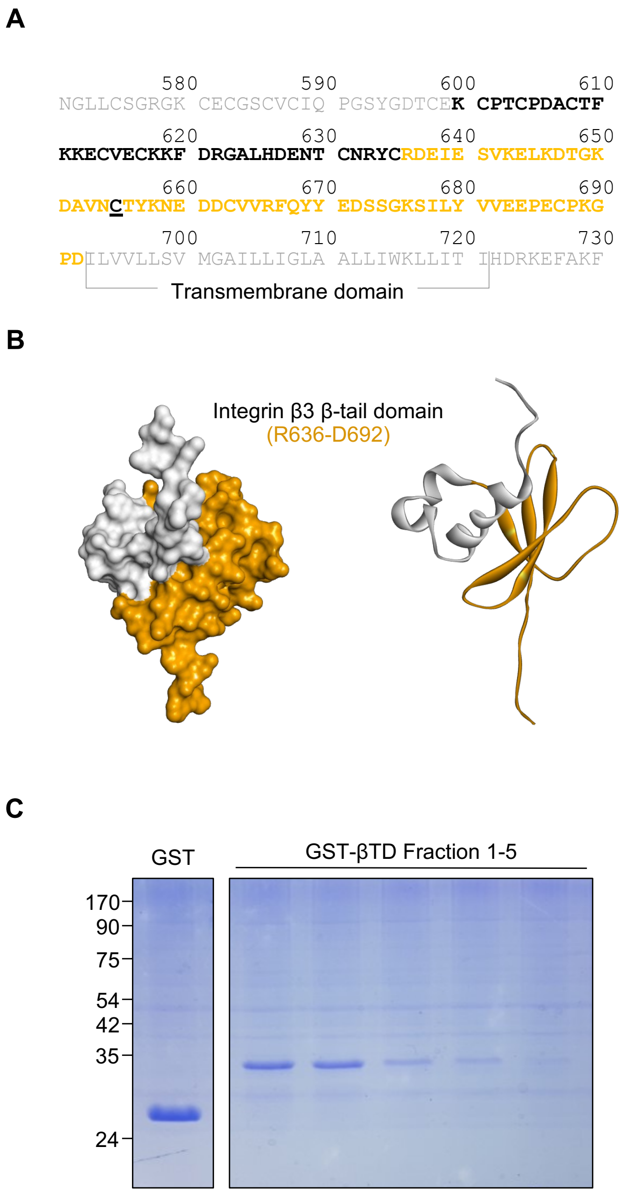

Supplemental Figure 1. β-tail domain (βTD) as an antigen. (A) Amino acid sequence of integrin β3 showing the βTD (bold) and transmembrane domain (boxed). The βTD segment used as a phage display antigen is highlighted in orange. Cysteine at position 655 was substituted with serine. (B) Structure of βTD (PDB ID: 3IJE) shown as surface and ribbon models. The antigenic region (Arg636–Asp692) is highlighted in orange. (C) SDS-PAGE analysis of purified GST and GST-βTD proteins stained with Coomassie blue.

Supplemental Figure 2

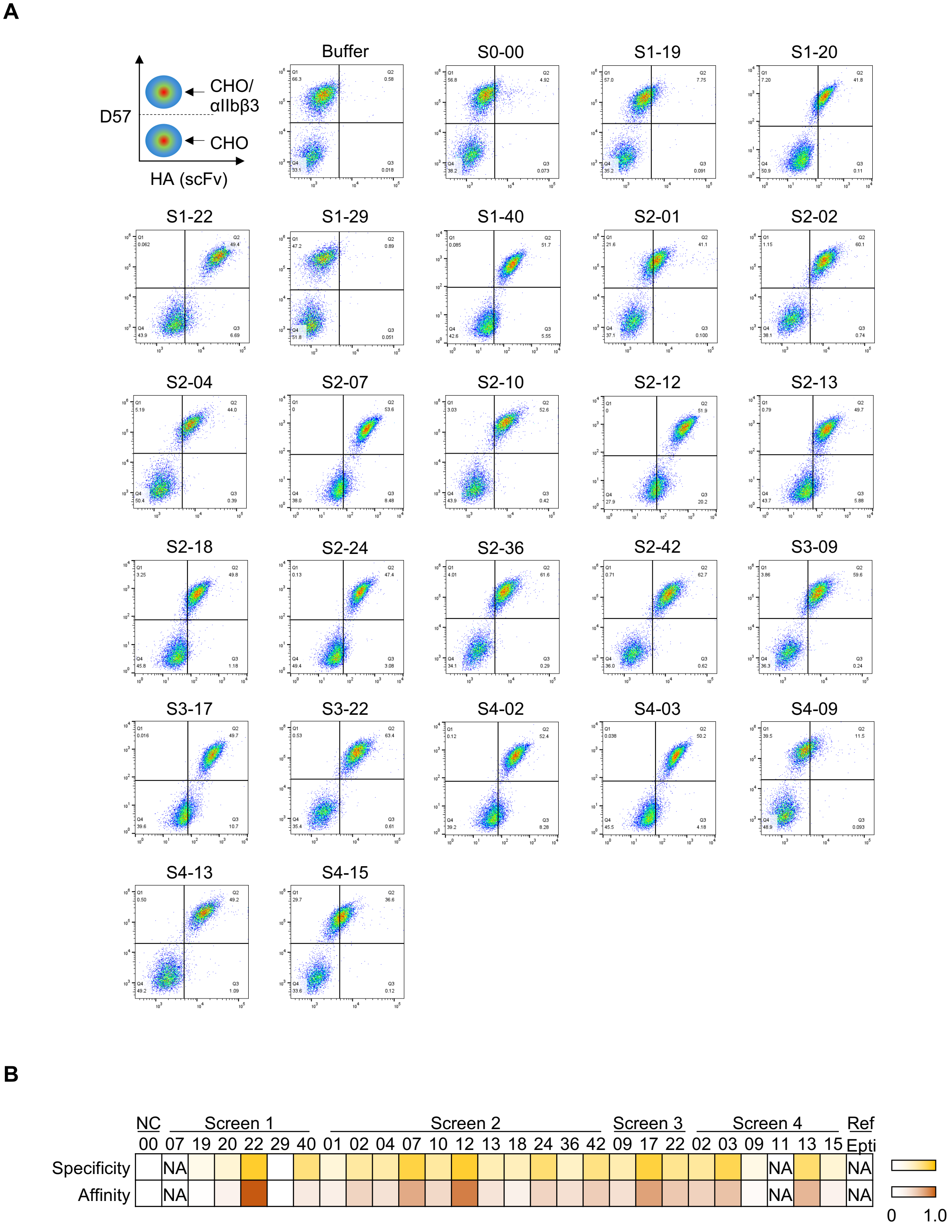

Supplemental Figure 2. Affinity and specificity of scFv clones. (A) Flow cytometry plots showing the binding of all screened scFv clones to CHO and CHO/αIIbβ3 cells, as described in Figure 1D. (B) Summary table of specificity and affinity measurements, performed as in Figure 1D.

Supplemental Figure 3

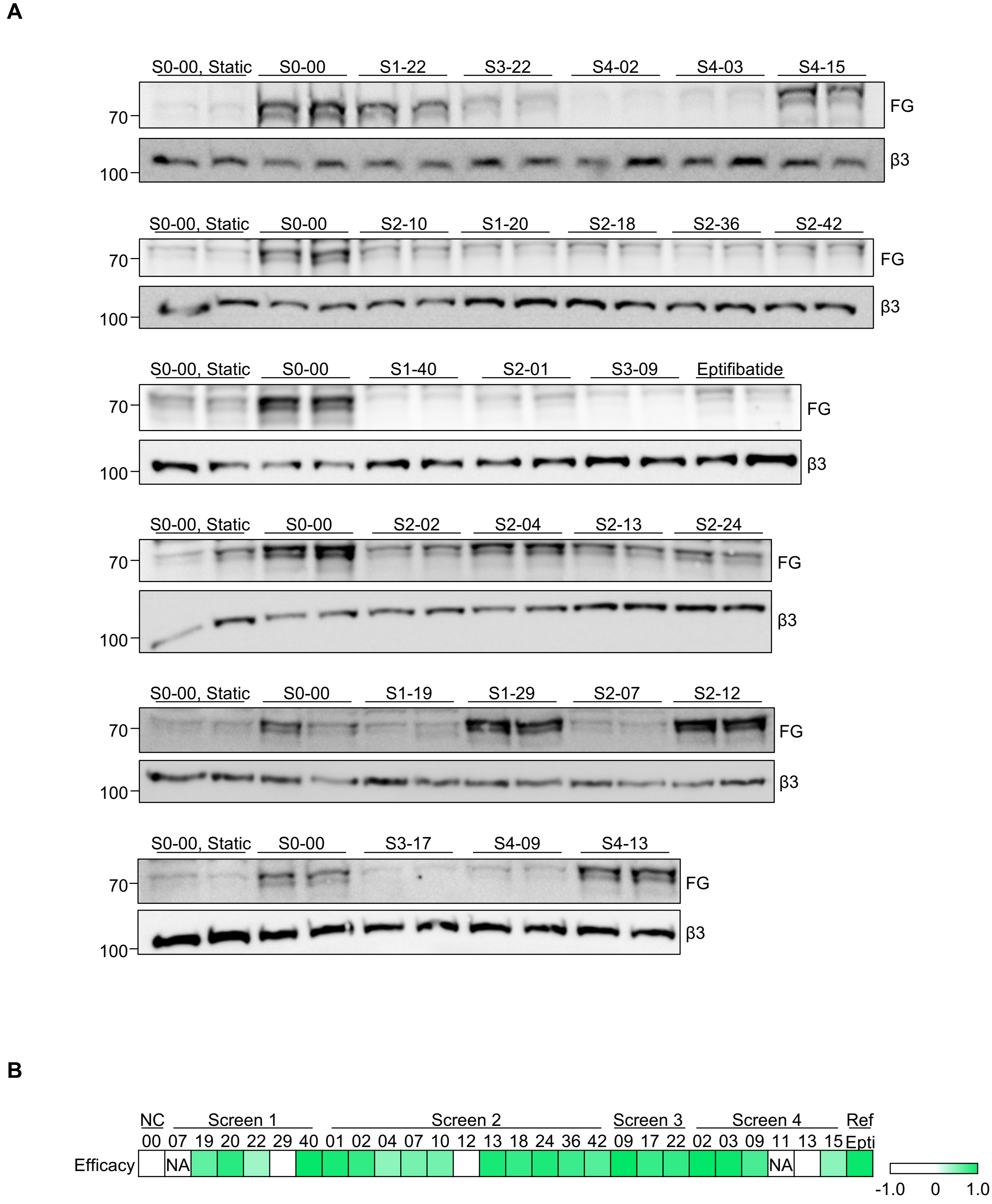

Supplemental Figure 3. Efficacy of scFv clones. (A) Effects of all screened clones on orbital shaking-induced fibrinogen binding were analyzed as described in Figure 1E and 1F. In each experiment, clones with similar *E. coli* expression levels (productivity) were compared. (B) Efficacy, defined as the inhibition of fibrinogen binding under orbital shaking, was measured as described in Figure 1F and is presented using a green gradient. Note that negative efficacy (i.e., enhanced fibrinogen binding) is displayed in white, the same color used to indicate clones with no detectable effect.

Supplemental Figure 4

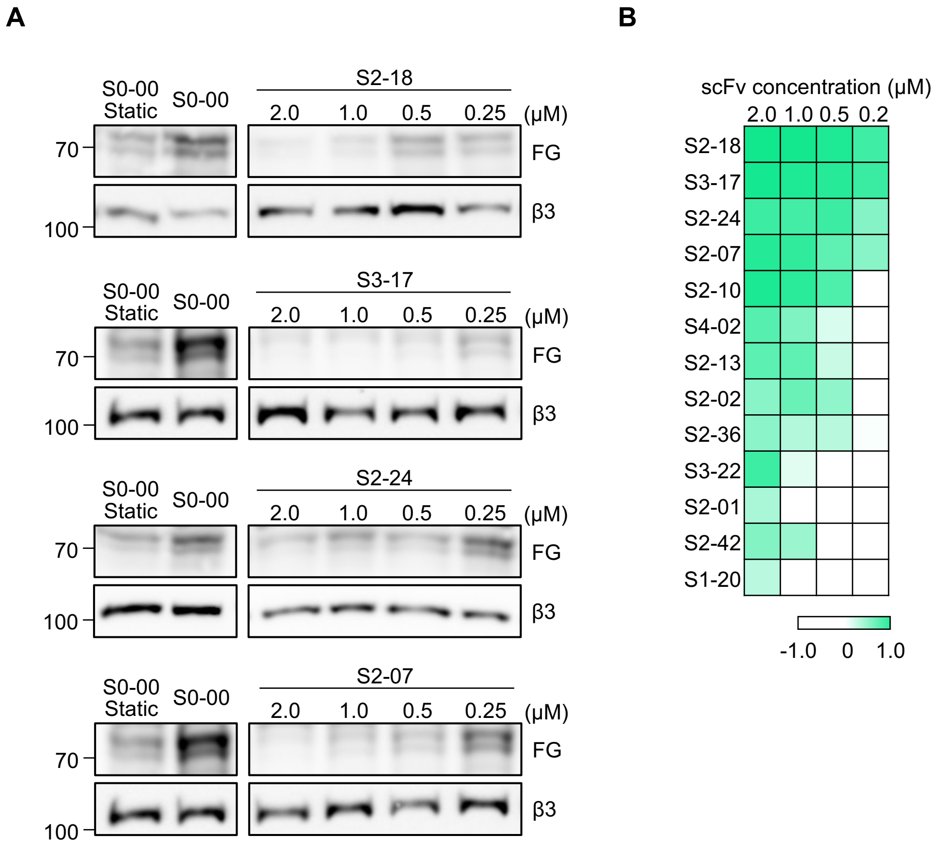

Supplemental Figure 4. Concentration-dependent efficacy of scFv clones. (A) High-efficacy clones were tested at 2, 1, 0.5, and 0.25 μM, as described in Figure 1F. Representative blots are shown. Note that S3-17 and S2-07 share the same control blots as they were analyzed simultaneously on the same gel. (B) Summary of concentration-dependent efficacy is presented as a green gradient as described in Figure 1F.

Supplemental Figure 5

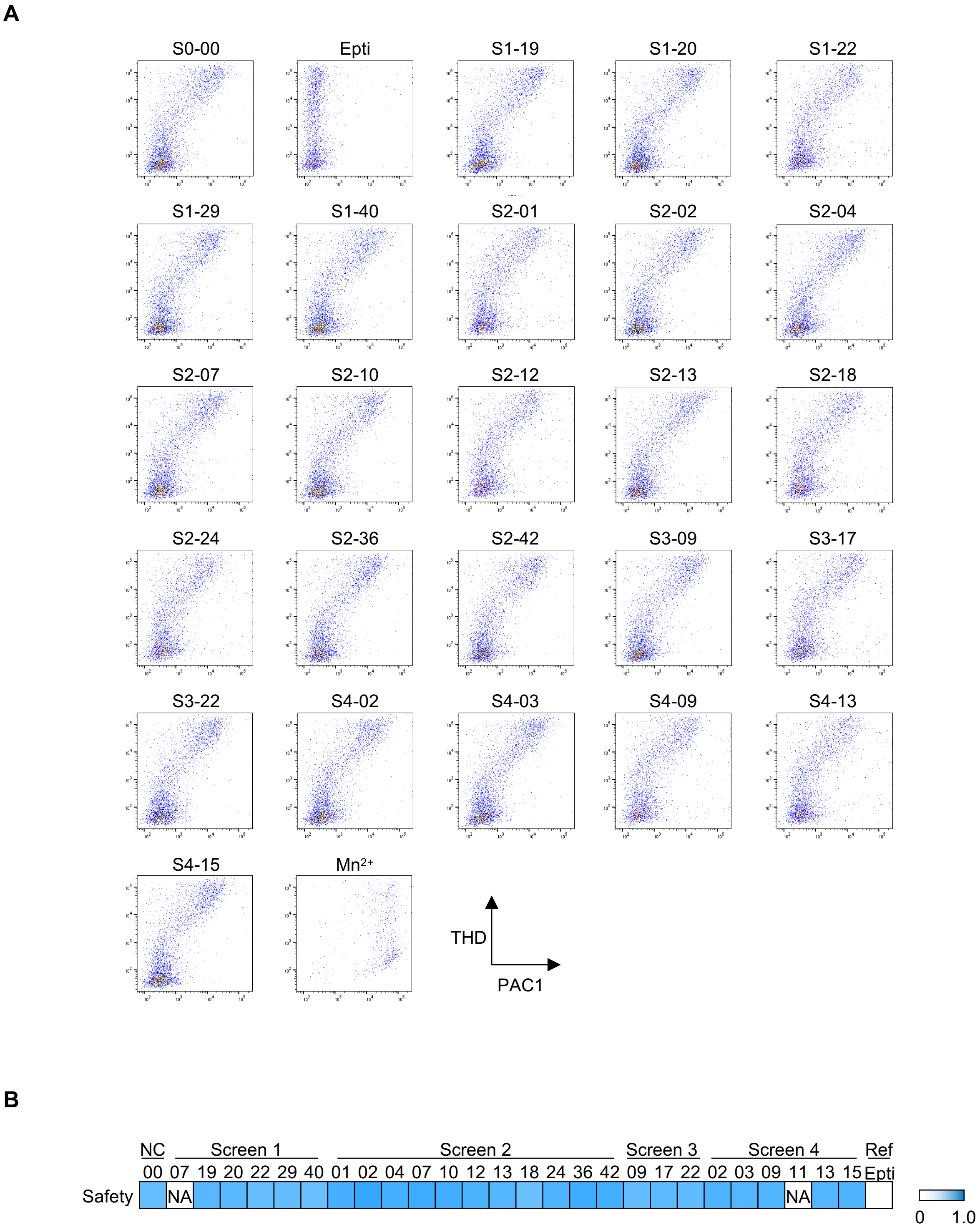

Supplemental Figure 5. Safety of scFv clones. (A) Effects of all screened clones on talin head domain-induced integrin activation were analyzed as described in Figure 1G. (B) Safety was measured as described in Figure 1G and summarized in a table.

Supplemental Figure 6

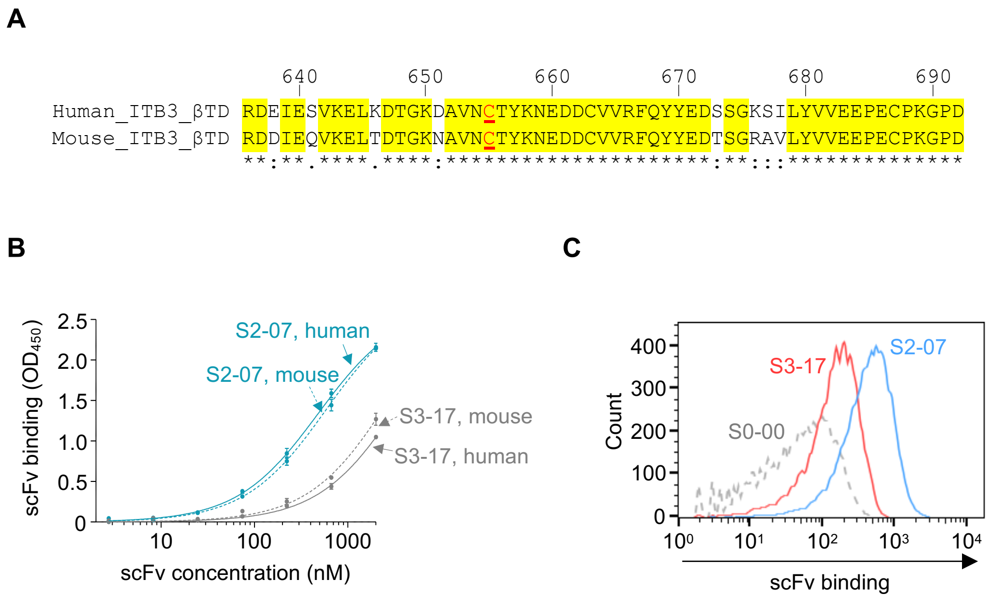

Supplemental Figure 6. Species specificity of S2-07 and S3-17. (A) Amino acid sequence alignment of the βTD from human and mouse integrin β3. (B) ELISA-based binding analysis of S2-07 (cyan) and S3-17 (gray) to purified human or mouse βTD. Binding curves were fitted using a one-site binding model. Dashed lines indicate binding to mouse βTD. Error bars indicate the mean ± SEM (n = 3). (C) Flow cytometry analysis of scFv (2 μM) binding to mouse platelets. S2-07 and S3-17 binding are shown as blue and red solid lines, respectively. S0-00 binding (gray) is shown as a negative control. Representative histogram is shown.

Supplemental Figure 7

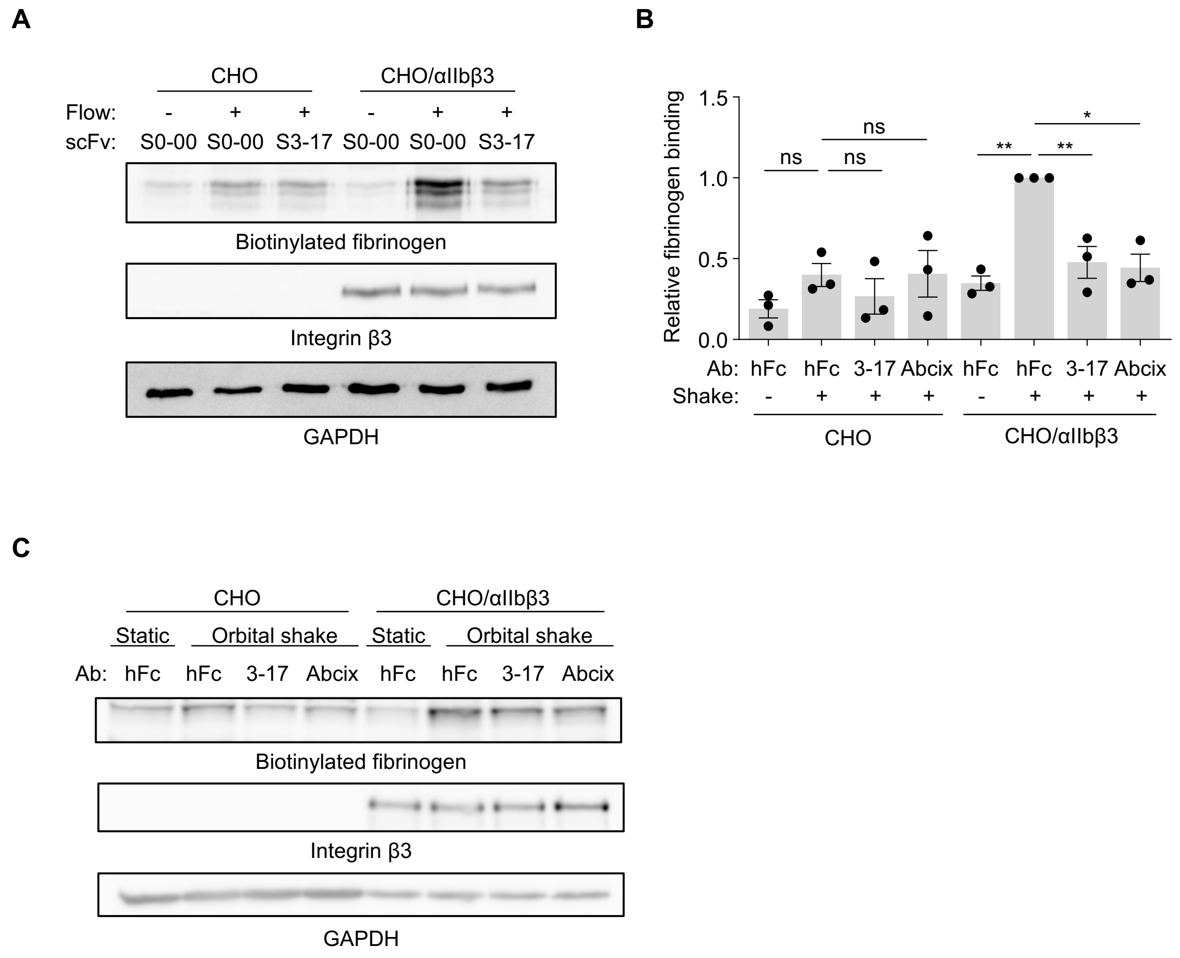

Supplemental Figure 7. Specificity of shear stress-dependent fibrinogen binding and its inhibition by 3-17. (A) CHO and CHO/αIIbβ3 cells were seeded in flow chamber and perfused with HBSS containing fibrinogen and 0.4 µM scFv, as in Figure2C. (B, C) CHO and CHO/αIIbβ3 cells were subjected to orbital shaking in the presence of 0.05 µM of hFc, 3-17Ab or abciximab (Abcix). Fibrinogen binding was analyzed by Western blotting and represented as a bar graph (n = 3). Error bars indicate the mean ± SEM. ∗, $p < 0.05$; ∗∗, $p < 0.01$; ns, not significant (one-way ANOVA using Tukey's multiple comparison test).

Supplemental Figure 8

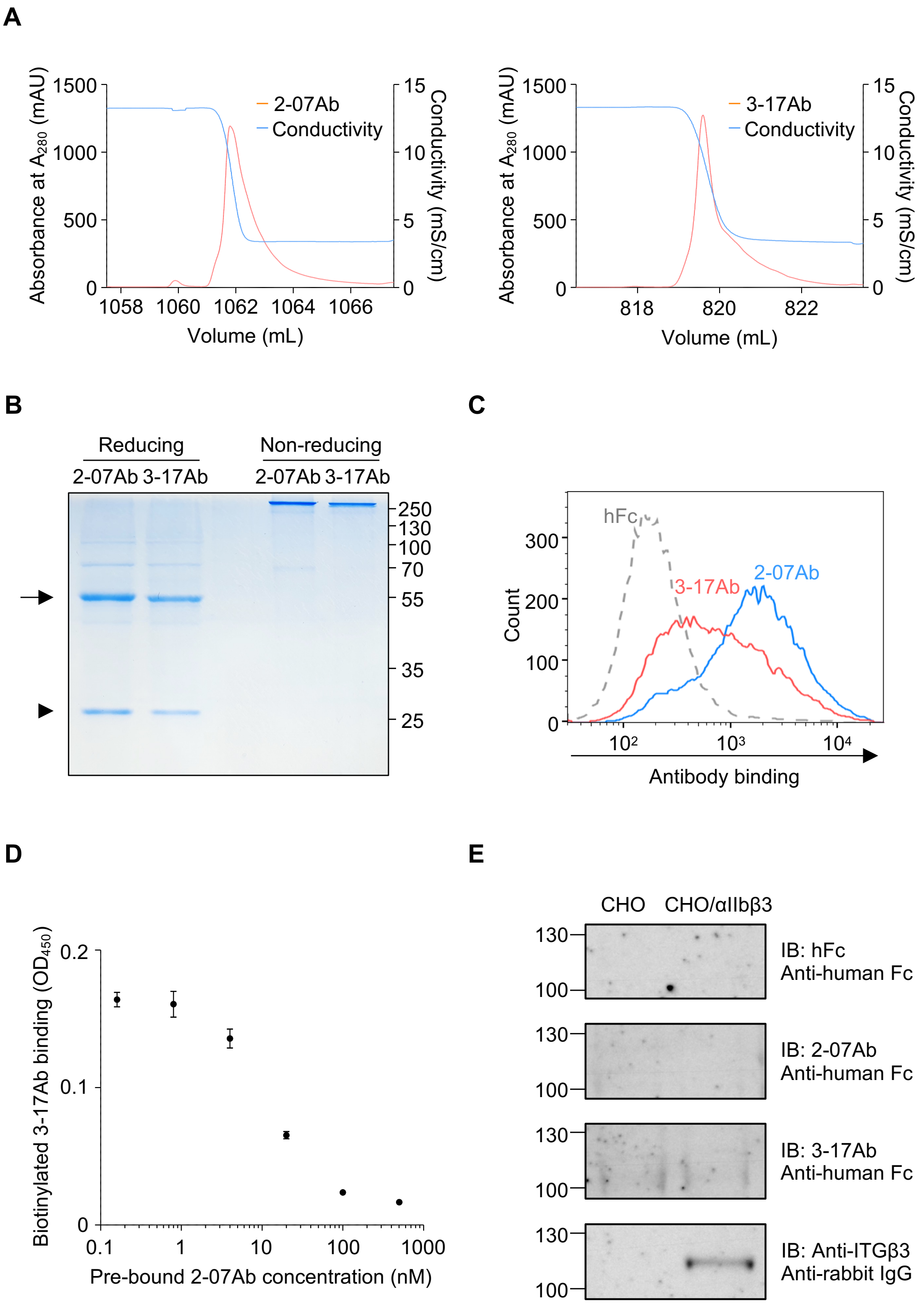

Supplemental Figure 8. Purification and characterization of 2-07Ab and 3-17Ab. (A) Elution profiles of 2-07Ab (left) and 3-17Ab (right) obtained from affinity chromatography. (B) SDS-PAGE and Coomassie blue staining of purified 2-07Ab and 3-17Ab under non-reducing and reducing conditions. Bands corresponding to the heavy chain and light chain of the antibody under reducing conditions are indicated by arrows and arrowheads, respectively. (C) Flow cytometry analysis of antibody (1 μM) binding to mouse platelets. 2-07Ab and 3-17Ab binding are shown as blue and red solid lines, respectively. Human Fc binding is shown as a negative control. Representative histogram is shown. (D) Competition of 2-07Ab and 3-17Ab was assessed by ELISA. Following pre-incubation of indicated concentration of 2-07Ab with immobilized β-tail domain, binding of biotinylated 3-17Ab (5 nM) was measured using HRP-conjugated streptavidin. Error bars indicate the mean ± SEM (n = 3). (E) CHO and CHO/αIIbβ3 cell lysates were separated by SDS-PAGE to generate linearized proteins. Binding of hFc, 2-07Ab, 3-17Ab (each at 1 μg/mL) and anti-integrin β3 antibody (Clone D7X3P, 66 ng/mL) to integrin β3 was analyzed by Western blotting.

Supplemental Figure 9

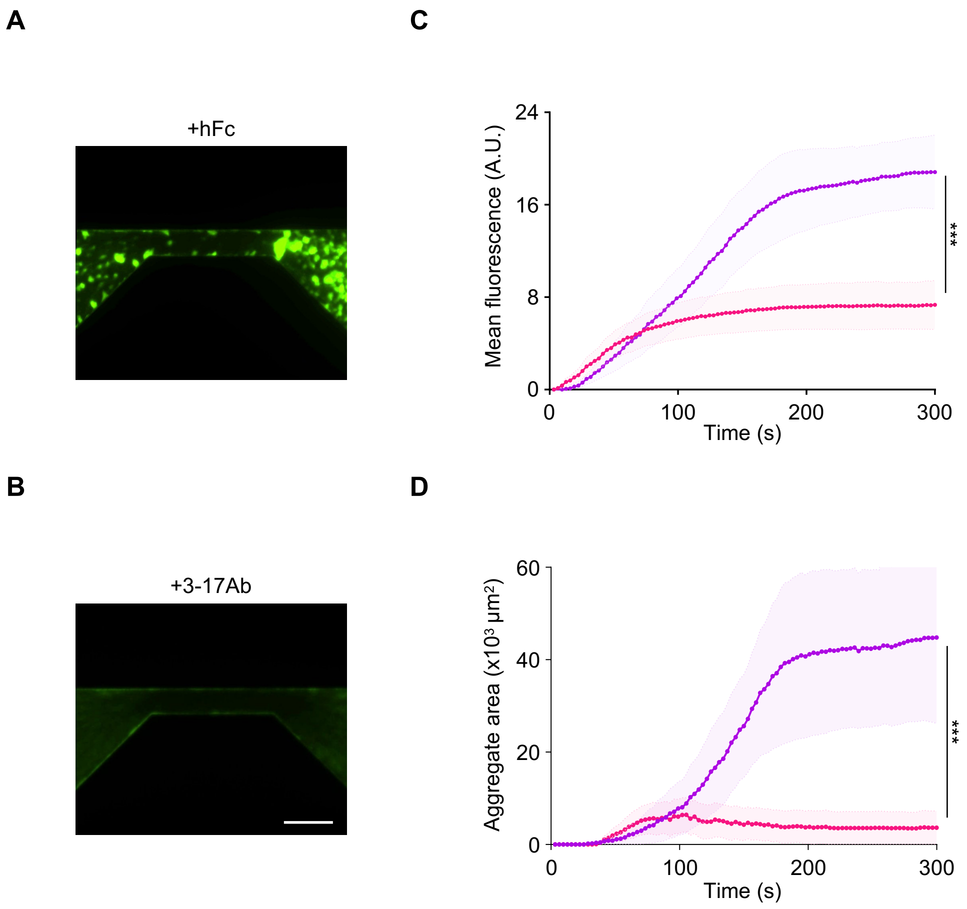

Supplemental Figure 9. Inhibitory effect of 3-17Ab on thrombus formation in stenotic flow chamber perfused with heparinized mouse whole blood. (A, B) Representative fluorescence images of the stenotic region (300 s) following perfusion of heparinized mouse whole blood in the presence of either hFc (A) or 3-17Ab (B). Platelets were stained by $DiOC_6$. Scale bar, 200 µm. (C, D) Quantification of the mean fluorescence intensity (C) and total area of platelet aggregates (D) in the stenotic region over time during perfusion (n = 4). Shaded areas represent mean ± SEM. ***, $p < 0.001$ (repeated measures two-way ANOVA).

Supplemental Figure 10

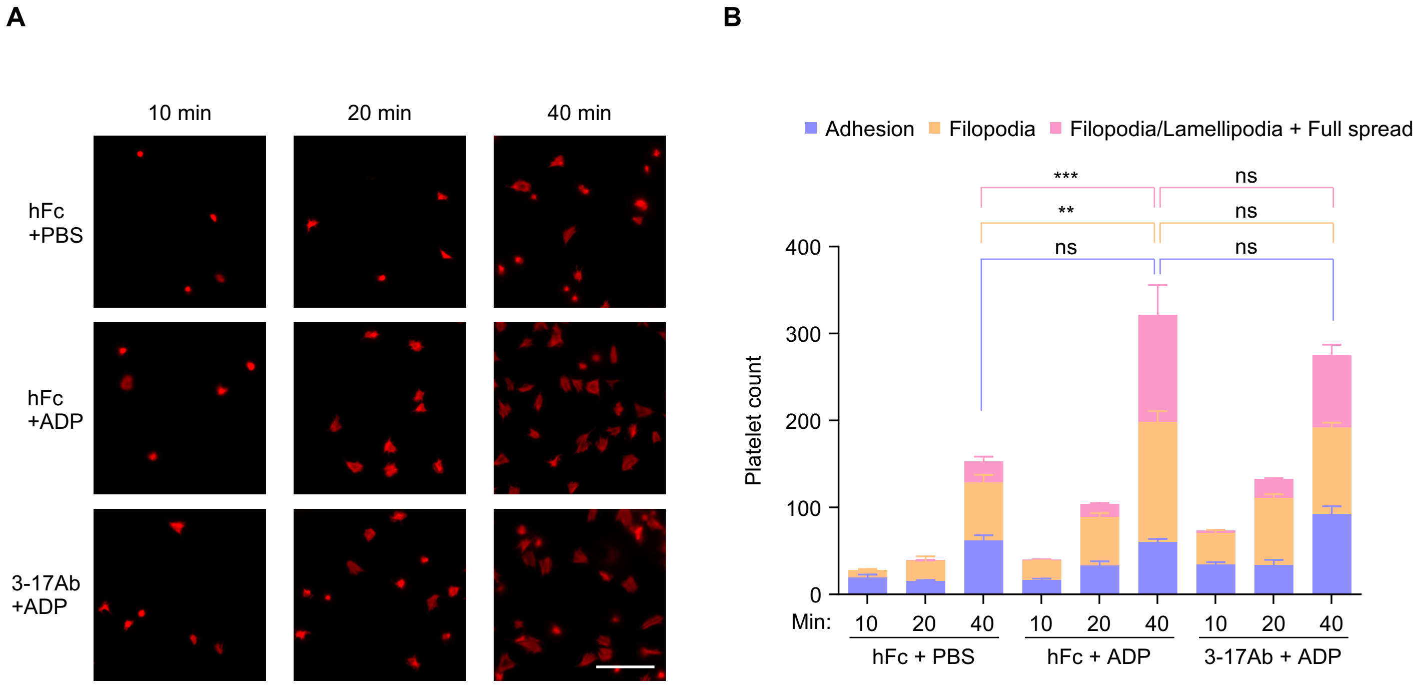

Supplemental Figure 10. Spreading kinetics of platelet treated with 3-17Ab on a fibrinogen-coated surface. (A) Mouse platelets were treated with 10 µM ADP (or PBS) in the presence of 1 µM hFc or 3-17Ab, and then plated on a fibrinogen-coated surface. At 10, 20 and 40 min post-seeding, the platelets were washed, fixed and stained with rhodamine-phalloidin. Representative fluorescence microscopy images are shown. Scale bar, 20 µm. (B) Distribution of platelet spreading stages at each time point from (A). The population of platelets at each stage—adhesion (light purple), filopodia (light orange), filopodia with lamellipodia and fully spread (pink)—was quantified from three experiments, with four random microscopic fields analyzed and pooled per sample. Error bars indicate the mean ± SEM. **, $p < 0.01$; ***, $p < 0.001$; ns, not significant (one-way ANOVA using Tukey's multiple comparison test).

Supplemental Figure 11

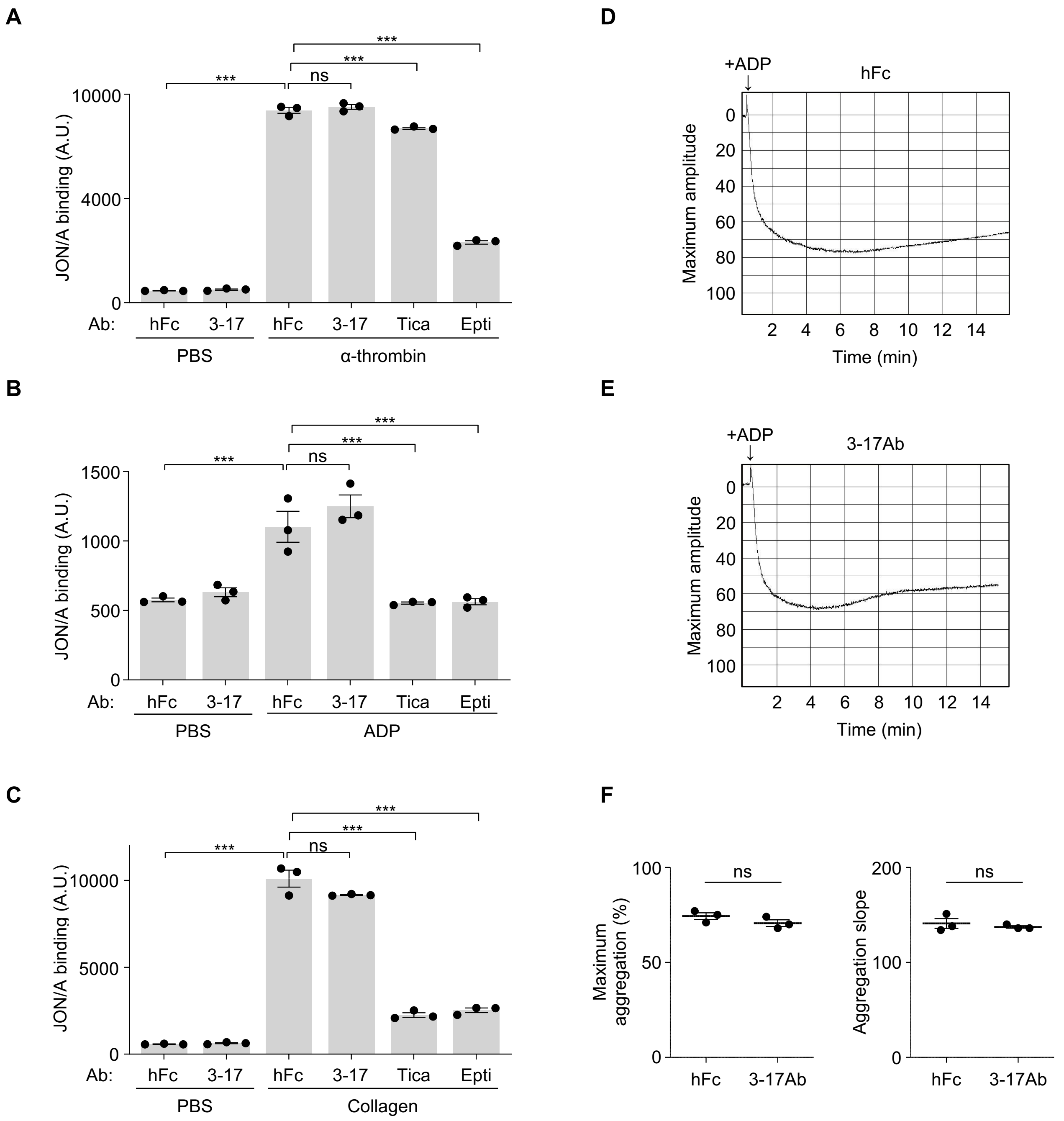

Supplemental Figure 11. Effects of 3-17Ab on agonist-induced platelet αIIbβ3 activation and aggregation. (A, B, C) Mouse platelets were pretreated with 0.1 U/mL α-thrombin (A), 10 μM ADP (B) or 20 μg/mL collagen (C) together with 1 μM hFc, 1 μM 3-17Ab, 1 μM ticagrelor, or 10 μM eptifibatide. Platelets were then stained with phycoerythrin (PE)-conjugated JON/A antibody and the binding was measured by flow cytometry (n = 3). (D, E) Light transmission aggregometry of citrated mouse platelet-rich plasma treated with 1 μM of hFc (D) or 3-17Ab (E). 10 μM of ADP was added to plasma at the beginning of the measurement. Representative results are shown. (F) Quantification of maximum aggregation (left) and aggregation slope (right) from (D) and (E). Results represent n = 3 measurements using two independently prepared pooled mouse PRP samples (4–8 mice per pool). Error bars indicate the mean ± SEM. ***, $p < 0.001$; ns, not significant (one-way ANOVA using Tukey's multiple comparison test).

Supplemental Figure 12

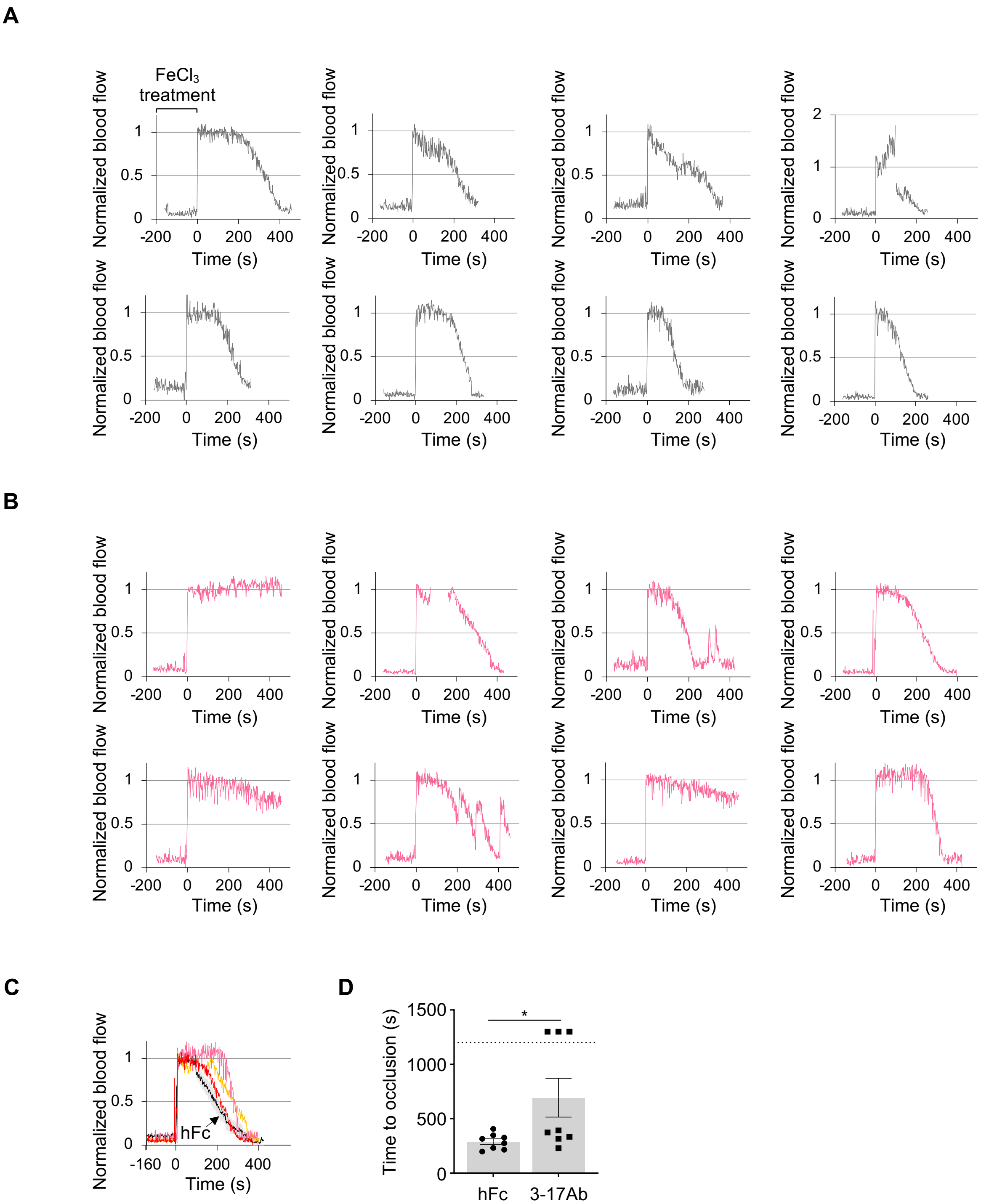

Supplemental Figure 12. Effects of 3-17Ab in the $FeCl_3$-induced carotid artery thrombosis model. (A) Complete dataset showing the effects of control hFc, as described in Figure 6B. (B) Complete dataset showing the effects of 3-17Ab, as described in Figure 6C. (C) Blood flow traces of the mean value from 8 hFc-injected mice from (A) shown in a black line, along with traces from three representative 3-17Ab–injected mice with delayed occlusion (red, orange, and pink). (D) Occlusion times from Figure 6D are shown as a bar graph, where each dot represents an individual mouse (n = 8). Error bars indicate the mean ± SEM. ∗, $p < 0.05$ (unpaired t-test with Welch's correction).

Supplemental Figure 13

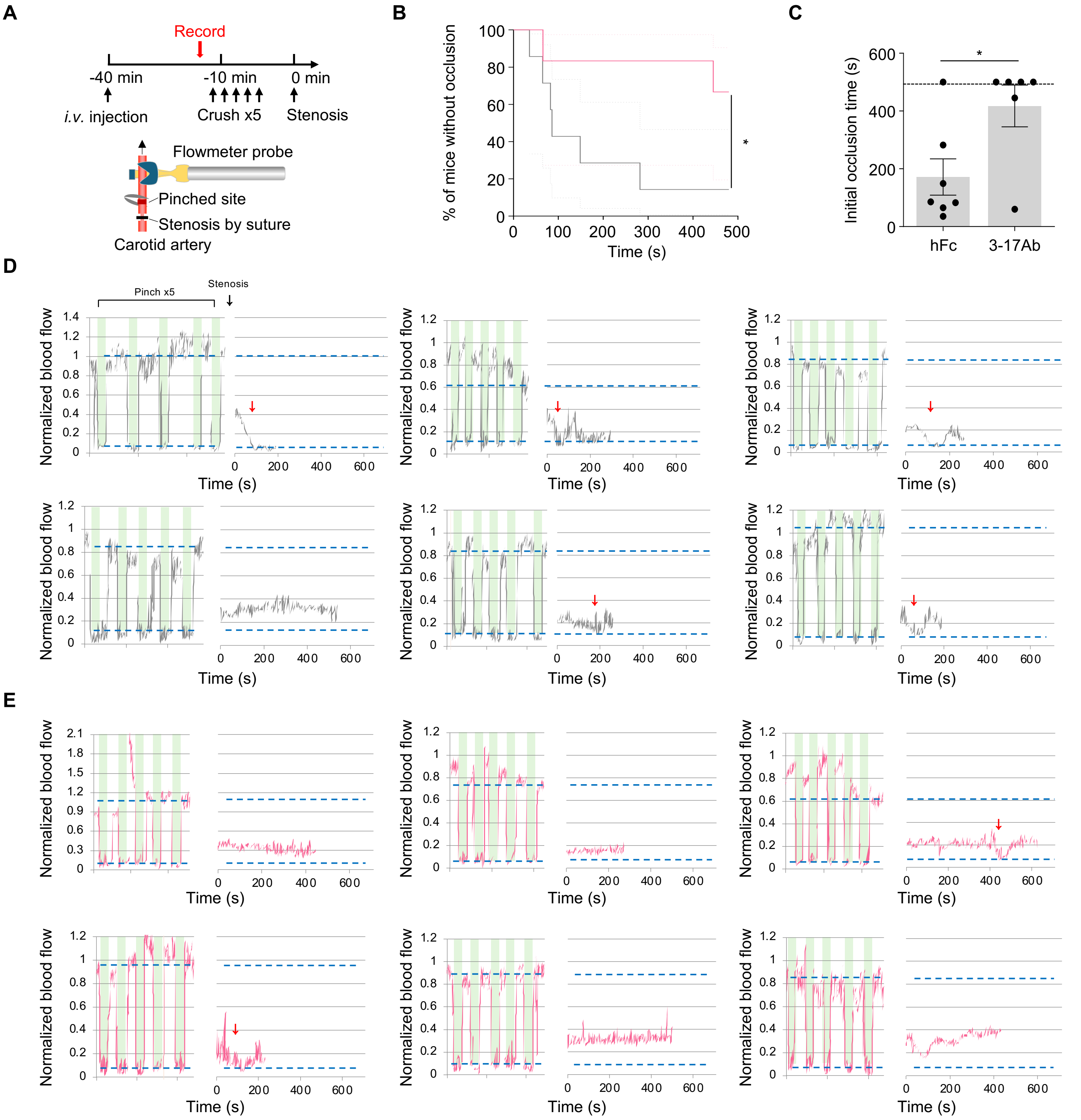

Supplemental Figure 13. Effects of 3-17Ab on thrombus formation in a mechanical injury and stenosis-induced carotid artery model. (A) Schematic illustration of the mechanical injury-induced thrombosis model. (B) Kaplan–Meier plot showing the proportion of mice maintaining normal blood flow over time. Occlusion occurrence time was defined as the blood flow rate reaches the baseline, where the baseline was calculated as the average blood flow across the five pinched regions. (n = 7 for hFc and n = 6 for 3-17Ab groups). Dotted lines indicate 95% confidence intervals. $*p < 0.05$ using the log-rank (Mantel–Cox) test. (C) Initial occlusion times from (B) are shown as a bar graph. Error bars indicate the mean ± SEM. $*$, $p < 0.05$ (unpaired t-test with Welch's correction). (D, E) Blood flow traces showing the flow rate in the carotid artery of mouse injected with hFc (D) or 3-17Ab (E). The green shaded region indicates the duration of the forceps-mediated arterial pinching (30 sec). The blue dashed lines represent baselines for normal flow (average flow rate pre-stenosis) or occluded flow from (average flow rate from five pinched regions). The flow rate was monitored for an additional 2 min after initial occlusion (red arrow) was observed, or for a maximum of 8 min if no occlusion occurred.

Supplemental Figure 14

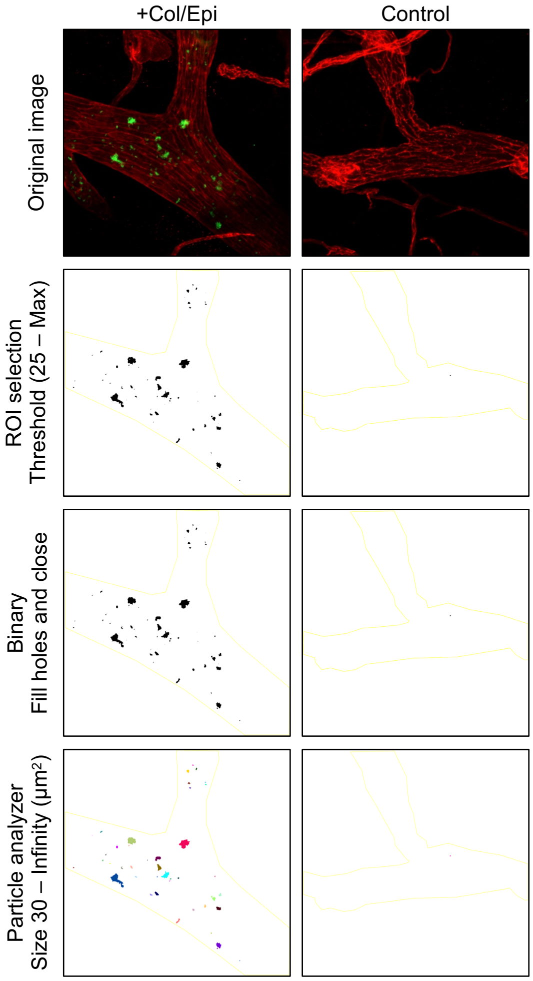

Supplemental Figure 14. Platelet aggregate detection using particle analysis. After selecting the region of interest (ROI) for the ear vasculature, images were binarized using a consistent fluorescence intensity threshold across all groups. Hole filling and morphological closing were applied in ImageJ, followed by particle analysis. Platelet aggregates larger than 30 μm² were selected, and their area was quantified.

Supplemental Figure 15

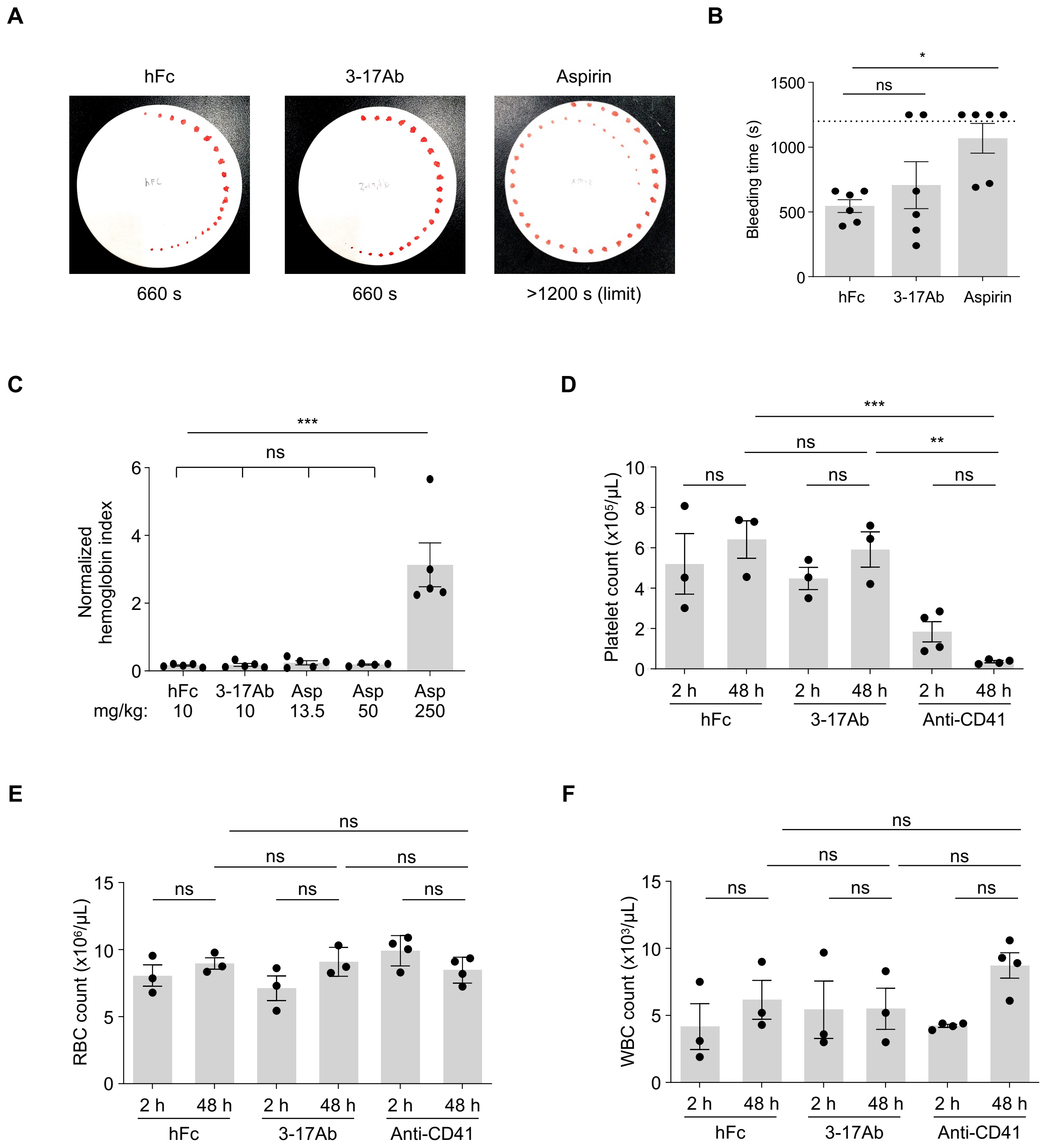

Supplemental Figure 15. In vivo hemostatic and hematological profiles of 3-17Ab. (A) Representative time-course of tail bleeding of mouse injected with 10 mg/kg of hFc, 3-17Ab or 13.5 mg/kg of aspirin. 1 h after injection, tail was amputated 3 mm from the tip and blotted onto filter paper every 30 s, up to 20 min to avoid lethality. (B) Quantification of tail bleeding time from (A) (n = 6). (C) Mouse fecal sample was collected 24 h after treatment with indicated dose of hFc, 3-17Ab or aspirin. Hemoglobin concentration in the fecal sample was measured via luminol chemiluminescence and normalized to fecal weight (n = 5). (D, E, F) Mice were injected with 10 mg/kg of hFc or 3-17Ab, or 0.75 mg/kg of anti-CD41 antibody. 2 h and 48 h post-injection, blood sample was collected and platelet counts (D), red blood cell counts (E) and white blood cell counts (F) were measured using an automated hematology analyzer (n = 3 for hFc and 3-17Ab groups, and n = 4 for anti-CD41 antibody group). Error bars indicate the mean ± SEM. *, $p < 0.05$, **, $p < 0.01$, ***, $p < 0.001$; ns, not significant (one-way ANOVA using Tukey's multiple comparison test).

Supplemental Figure 16

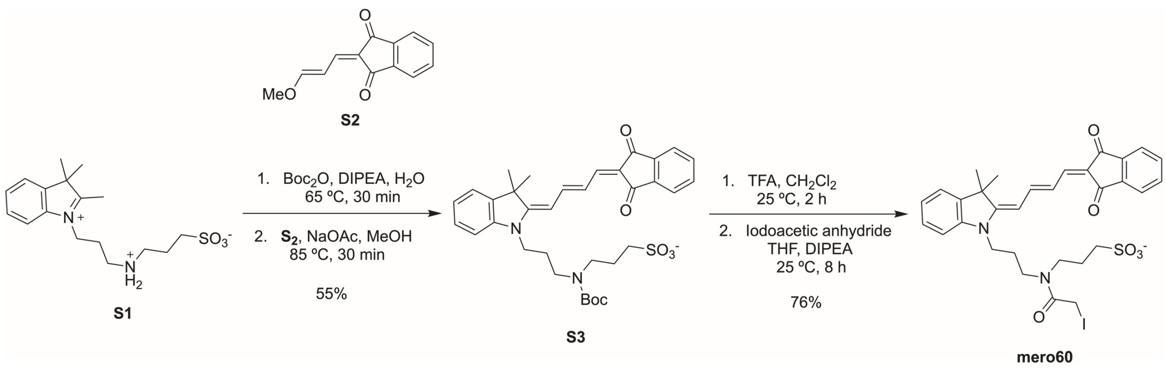

Supplemental Figure 16. Synthesis of Mero60. Synthetic route from S1 and S2 via intermediate S3 to afford mero60. Reaction conditions and full characterization data are provided in the Methods section.

Supplemental Figure 17

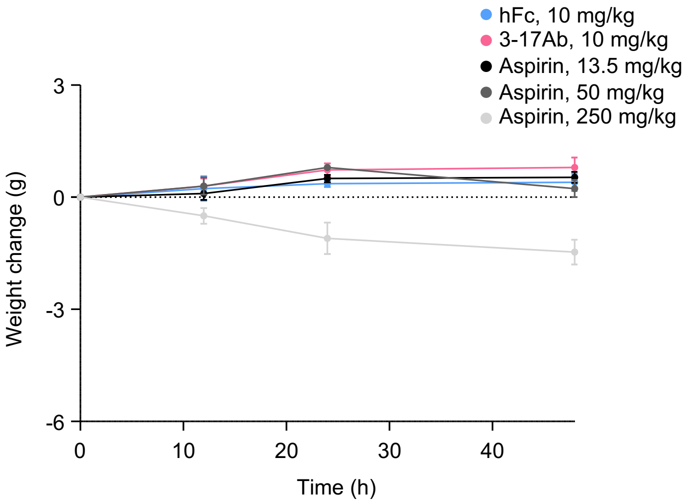

Supplemental Figure 17. Monitoring of body weight changes in mice. Body weights were measured at 12, 24, and 48 h following intravenous injection of the indicated doses of hFc, 3-17Ab, or aspirin. Data are presented as change from baseline (initial weight: 25–28 g). Error bars indicate the mean ± SEM (n = 3).
