# Supplementary material for: Selective Inhibition of Integrin β3 Topology Provides a Safer Antithrombotic Strategy
Source: Adv Sci (Weinh). 2026 Apr 13;13(39):e22086. doi: 10.1002/advs.202522086 (PMC13334950; doi:10.1002/advs.202522086)
Supplement: Supplementary file 2 — Supporting File 2: advs75275‐sup‐0002‐Data.zip. [file ADVS-13-e22086-s003.zip › advs75275-sup-0002-Data/260403_Adv. Sci. Uncropped gels and membranes.pdf]

Figure 1C

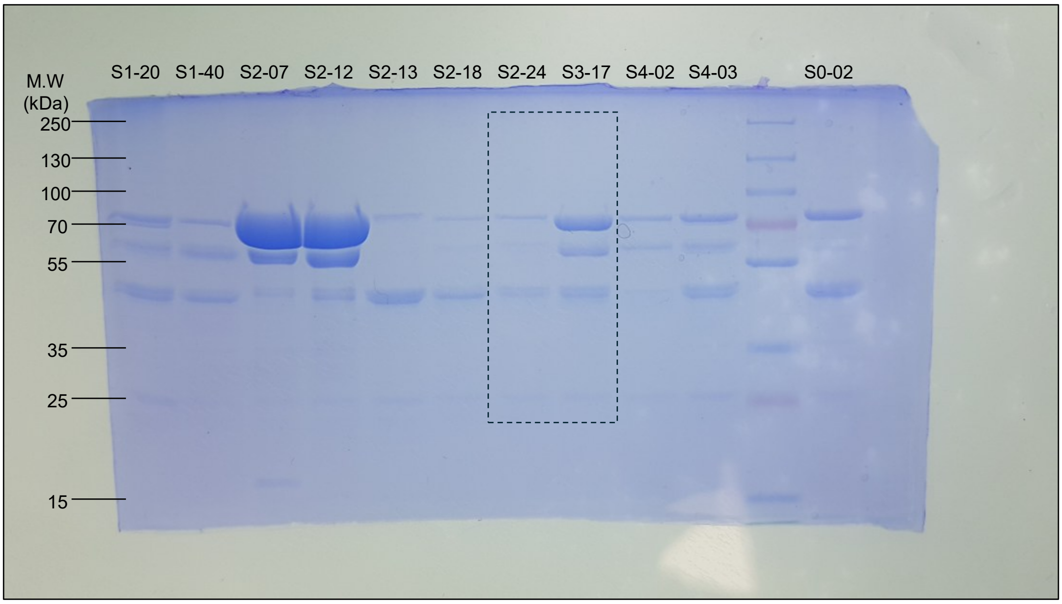

M.W
(kDa)
250
130
100
70
55
35
25
15
S1-20
S1-40
S2-07
S2-12
S2-13
S2-18
S2-24
S3-17
S4-02
S4-03
S0-02

Figure 1F

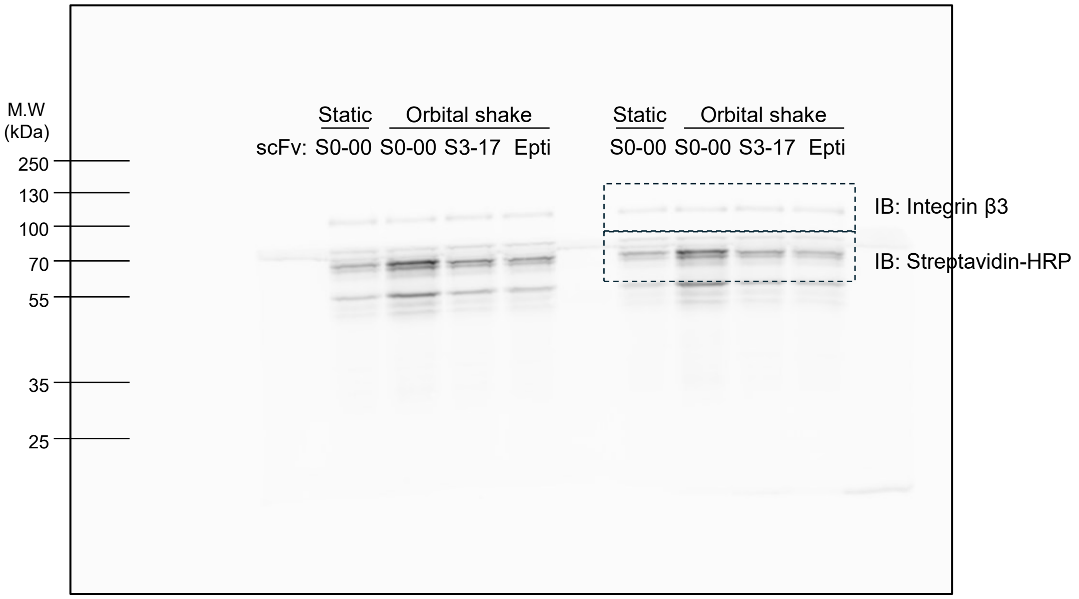
M.W
(kDa)
250
130
100
70
55
35
25
Static
Orbital shake
Static
Orbital shake
scFv: S0-00 S0-00 S3-17 Epti
S0-00 S0-00 S3-17 Epti
IB: Integrin β3
IB: Streptavidin-HRP

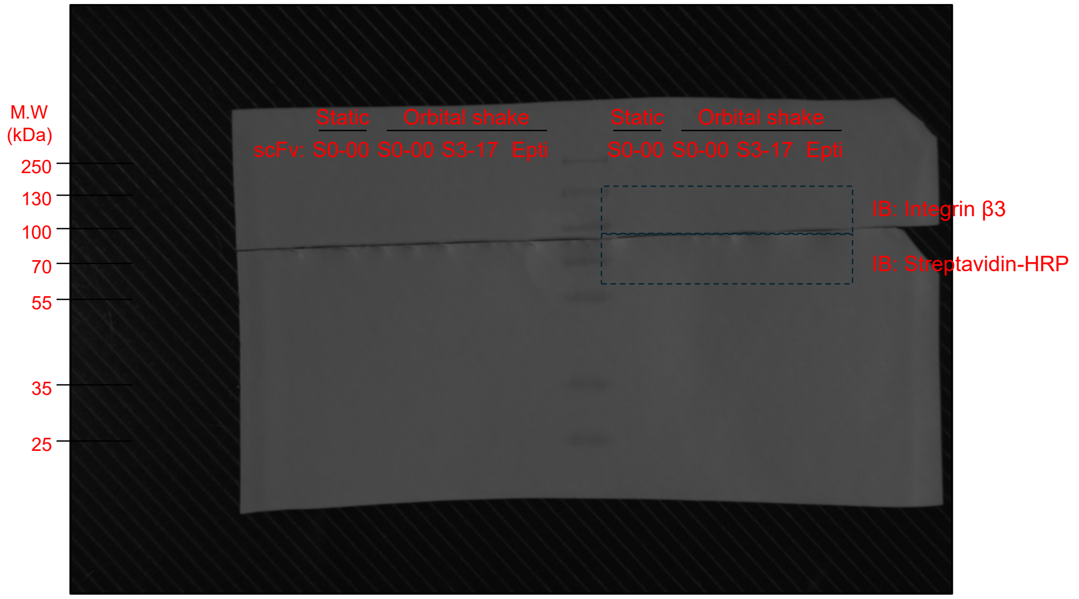
M.W
(kDa)
250
130
100
70
55
35
25
Static
Orbital shake
Static
Orbital shake
scFv: S0-00 S0-00 S3-17 Epti
S0-00 S0-00 S3-17 Epti
IB: Integrin β3
IB: Streptavidin-HRP

Figure 2C

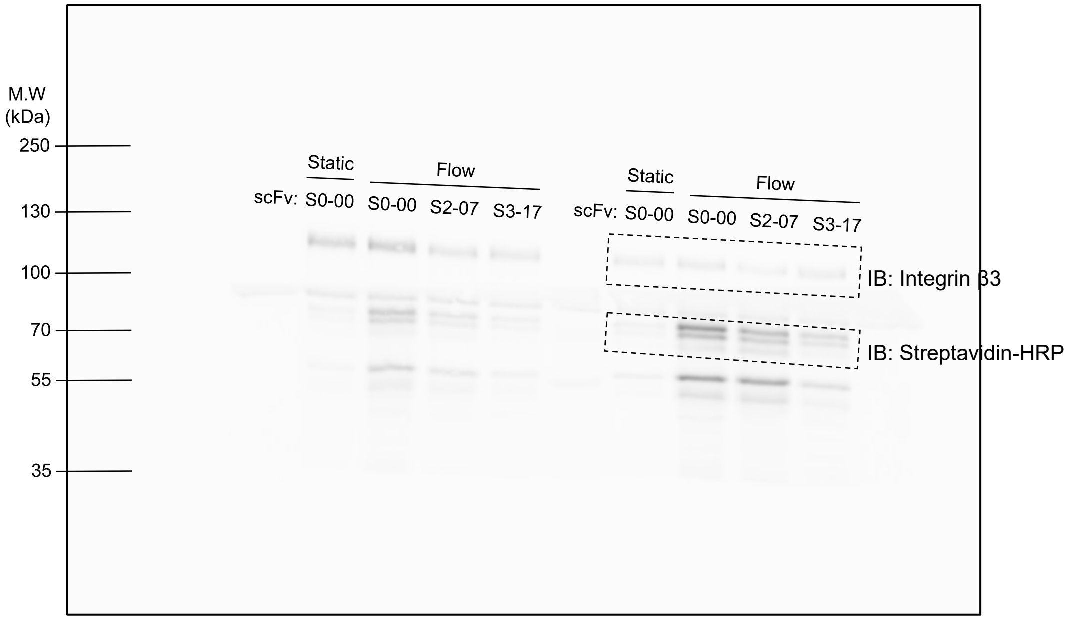
M.W
(kDa)
250
130
100
70
55
35
Static
Flow
scFv: S0-00
S0-00
S2-07
S3-17
Static
Flow
scFv: S0-00
S0-00
S2-07
S3-17
IB: Integrin β3
IB: Streptavidin-HRP

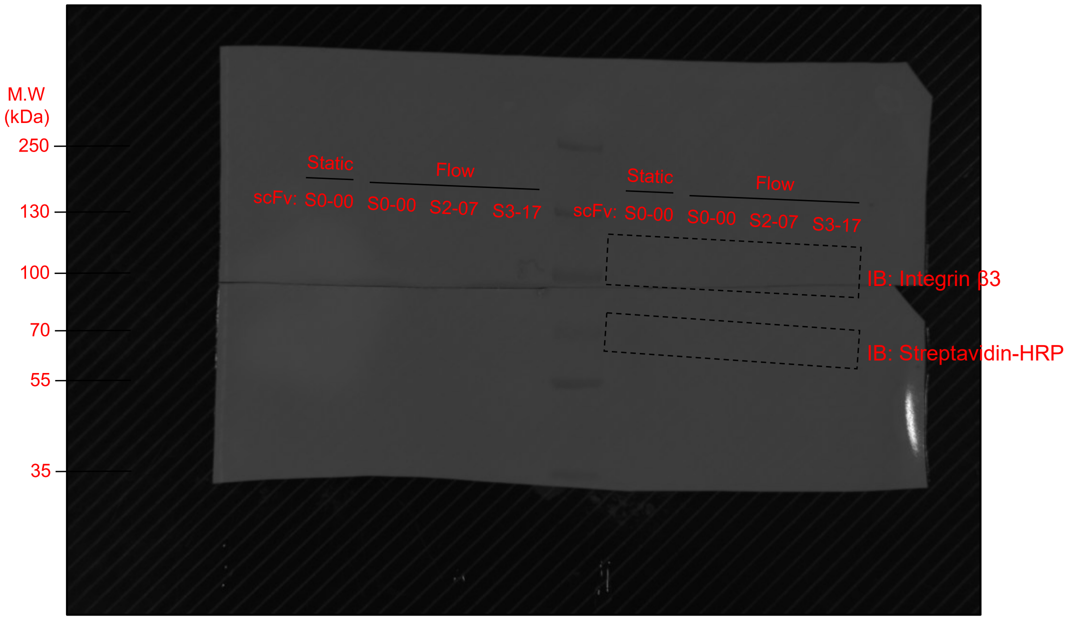
M.W
(kDa)
250
130
100
70
55
35
Static
Flow
scFv: S0-00
S0-00
S2-07
S3-17
Static
Flow
scFv: S0-00
S0-00
S2-07
S3-17
IB: Integrin β3
IB: Streptavidin-HRP

Figure 2F

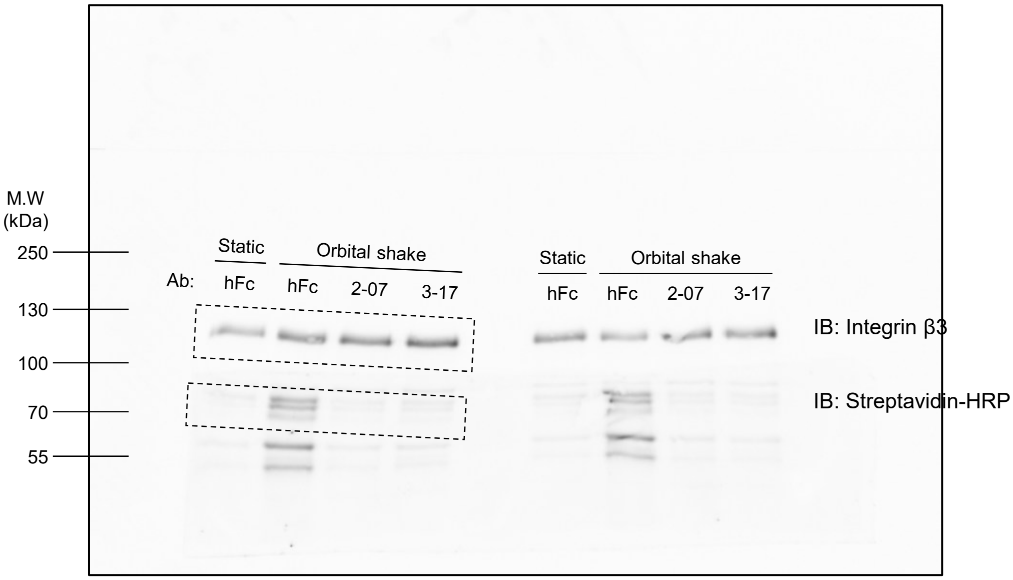
M.W
(kDa)
250
130
100
70
55
Ab:
Static
Orbital shake
hFc
hFc
2-07
3-17
Static
Orbital shake
hFc
hFc
2-07
3-17
IB: Integrin β3
IB: Streptavidin-HRP

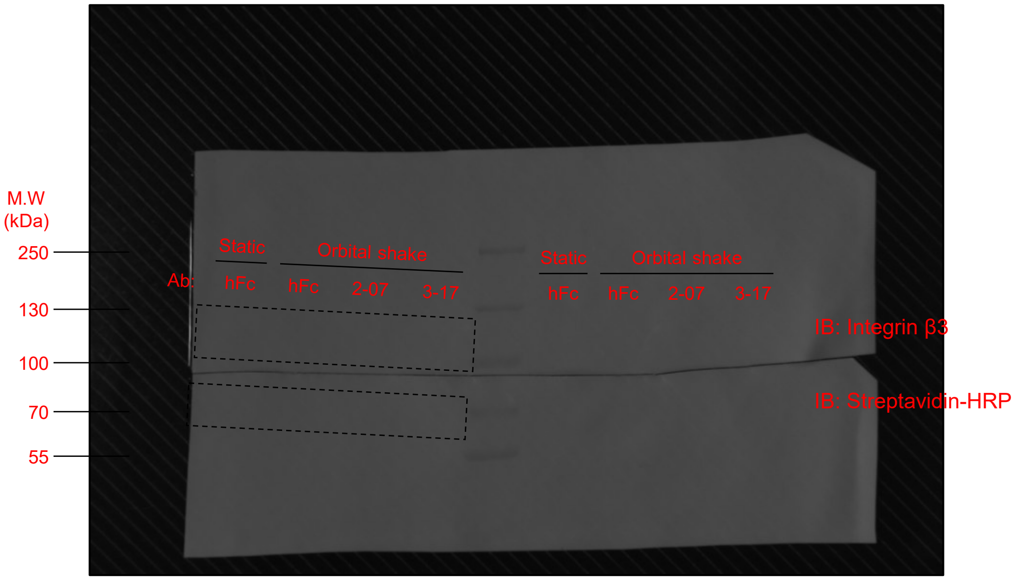
M.W
(kDa)
250
130
100
70
55
Ab:
Static
Orbital shake
hFc
hFc
2-07
3-17
Static
Orbital shake
hFc
hFc
2-07
3-17
IB: Integrin β3
IB: Streptavidin-HRP

Figure 3B

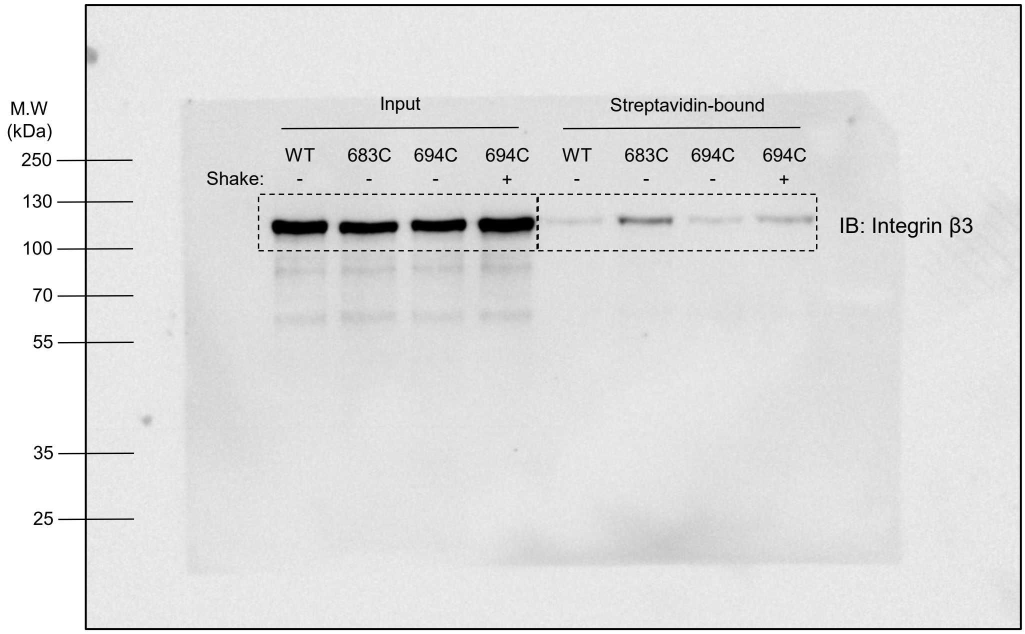
M.W
(kDa)
250
130
100
70
55
35
25
Input
Streptavidin-bound
WT 683C 694C 694C WT 683C 694C 694C
Shake: - - - + - - - +
IB: Integrin β3

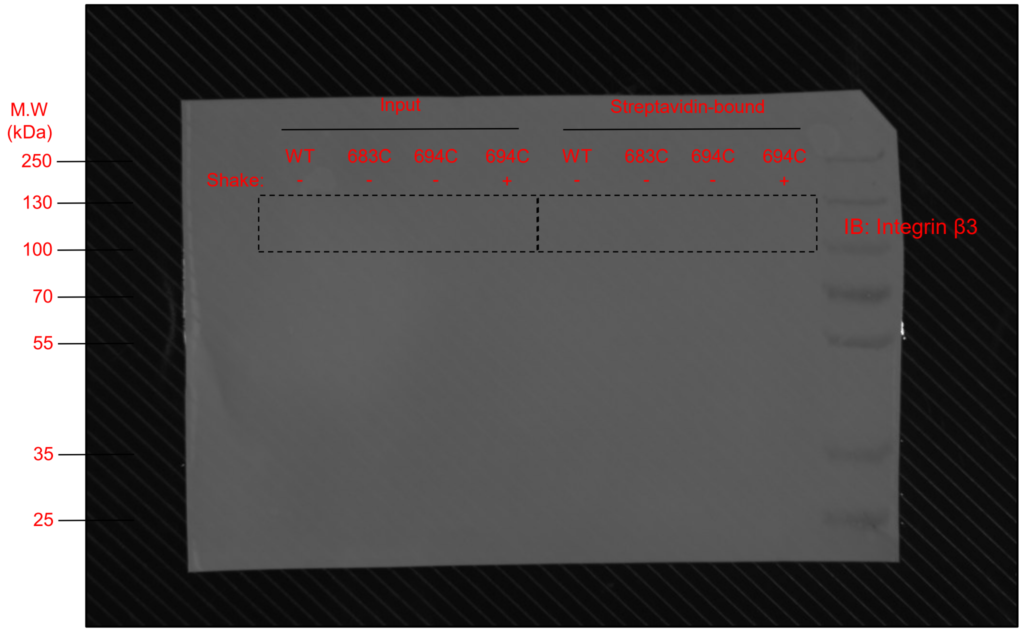
M.W
(kDa)
250
130
100
70
55
35
25
Input
Streptavidin-bound
WT 683C 694C 694C WT 683C 694C 694C
Shake: - - - + - - - +
IB: Integrin β3

Figure 3D

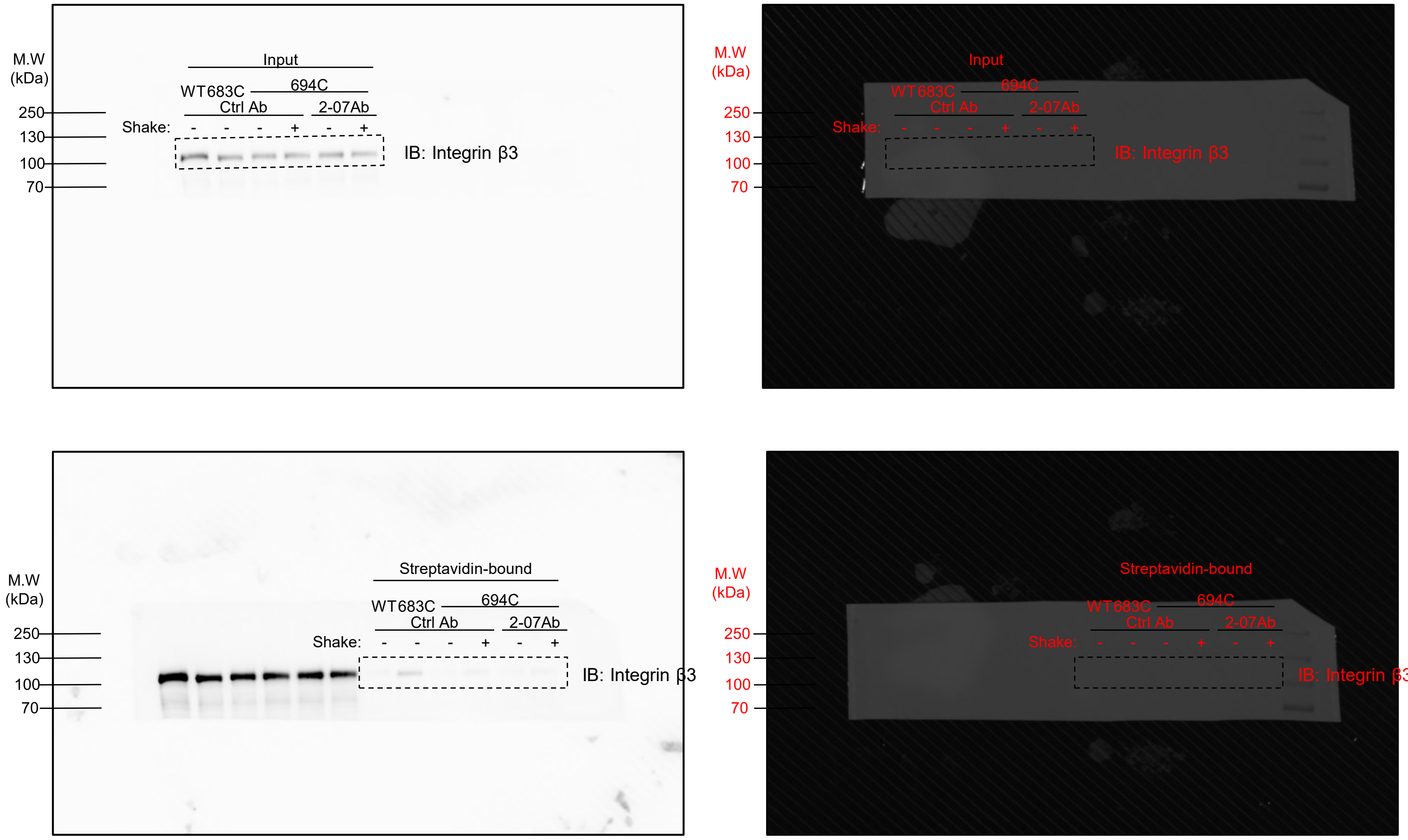

M.W
(kDa)
250
130
100
70
Input
WT683C
694C
Ctrl Ab
2-07Ab
Shake: - - - + - +
IB: Integrin β3
M.W
(kDa)
250
130
100
70
Input
WT683C
694C
Ctrl Ab
2-07Ab
Shake: - - - + - +
IB: Integrin β3
M.W
(kDa)
250
130
100
70
Streptavidin-bound
WT683C
694C
Ctrl Ab
2-07Ab
Shake: - - - + - +
IB: Integrin β3
M.W
(kDa)
250
130
100
70
Streptavidin-bound
WT683C
694C
Ctrl Ab
2-07Ab
Shake: - - - + - +

Figure 3F

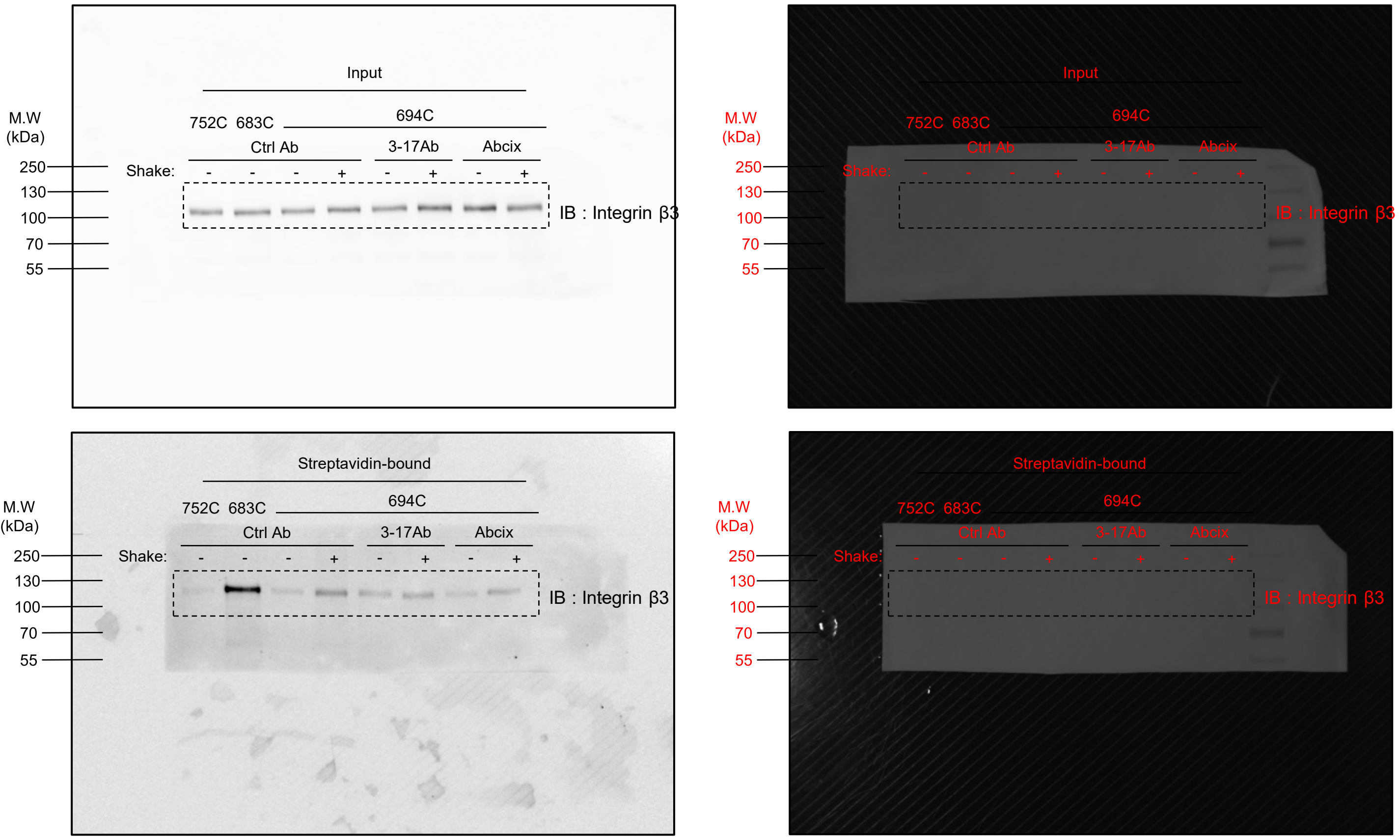
Input
M.W
(kDa)
752C 683C
694C
Ctrl Ab
3-17Ab
Abcix
Shake: - - - + - + - +
250
130
100
70
55
IB : Integrin β3
Streptavidin-bound
M.W
(kDa)
752C 683C
694C
Ctrl Ab
3-17Ab
Abcix
Shake: - - - + - + - +
250
130
100
70
55
IB : Integrin β3

Figure 4F

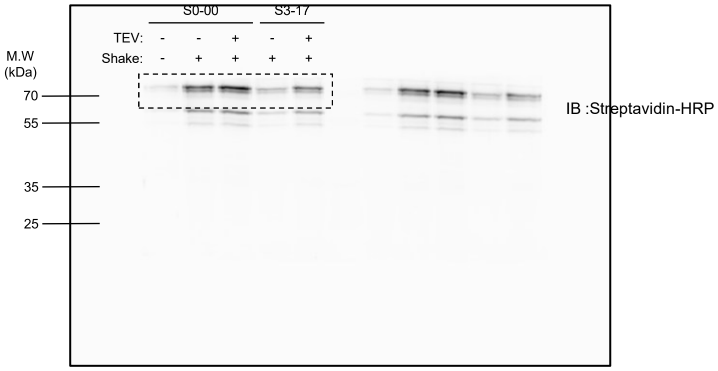
S0-00
S3-17
TEV: - - + - +
Shake: - + + + +
M.W
(kDa)
70
55
35
25
IB :Streptavidin-HRP

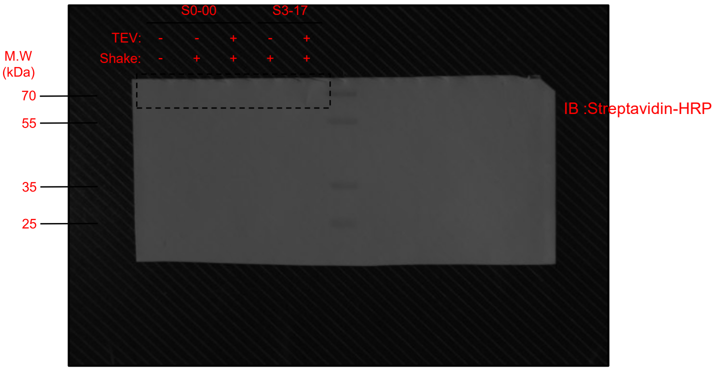
S0-00
S3-17
TEV: - - + - +
Shake: - + + + +
M.W
(kDa)
70
55
35
25
IB :Streptavidin-HRP

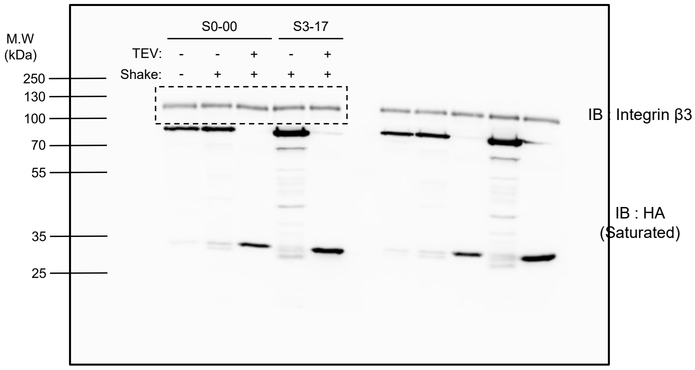
S0-00
S3-17
TEV: - - + - +
Shake: - + + + +
M.W
(kDa)
250
130
100
70
55
35
25
IB : Integrin β3
IB : HA
(Saturated)

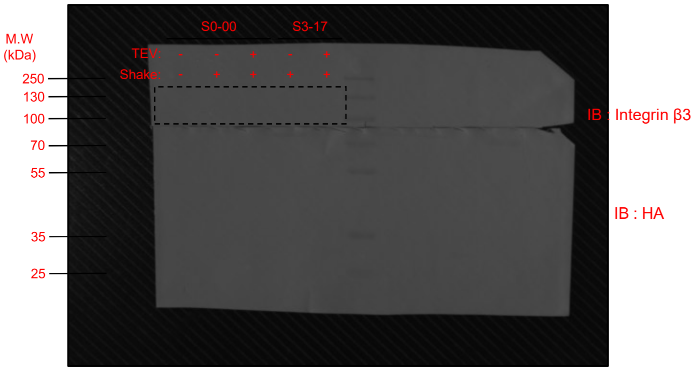
S0-00
S3-17
TEV: - - + - +
Shake: - + + + +
M.W
(kDa)
250
130
100
70
55
35
25
IB : Integrin β3
IB : HA

Figure S1C

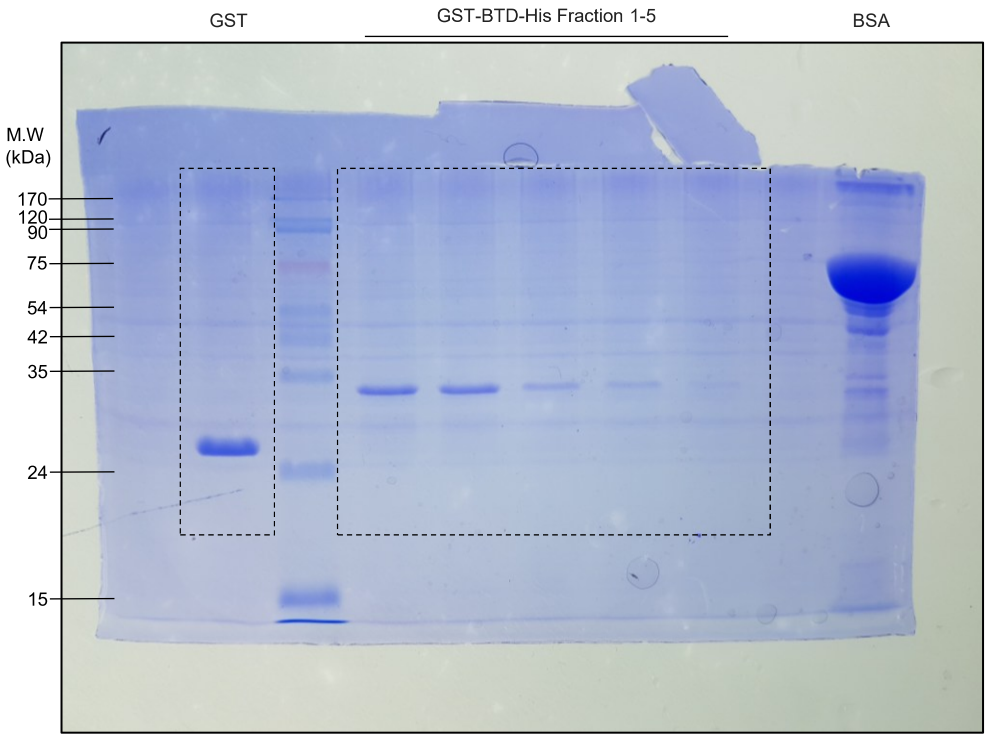
GST
GST-BTD-His Fraction 1-5
BSA
M.W
(kDa)
170
120
90
75
54
42
35
24
15

Figure S3A

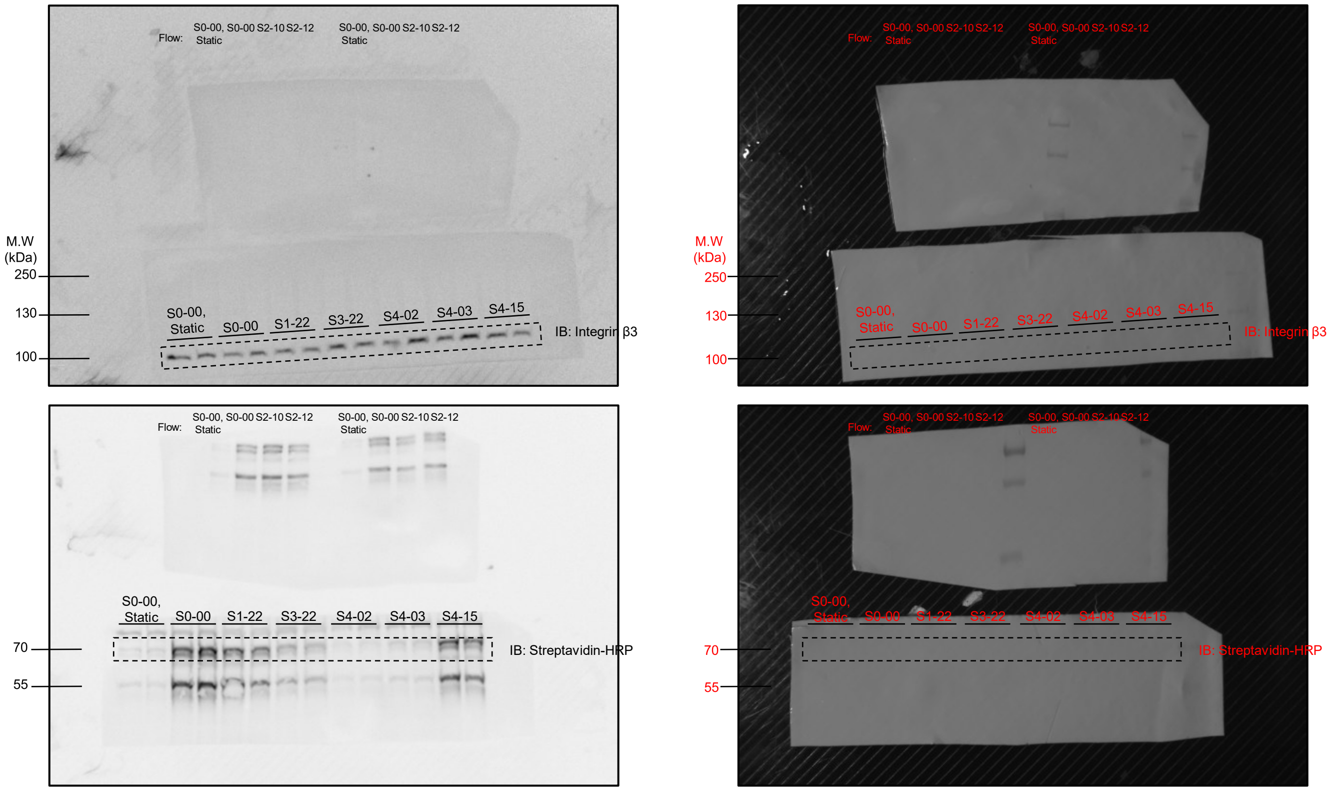

Flow:
S0-00, Static
S0-00
S2-10
S2-12
M.W (kDa)
250
130
100
S0-00, Static
S0-00
S1-22
S3-22
S4-02
S4-03
S4-15
IB: Integrin β3
70
55
IB: Streptavidin-HRP

Figure S3A

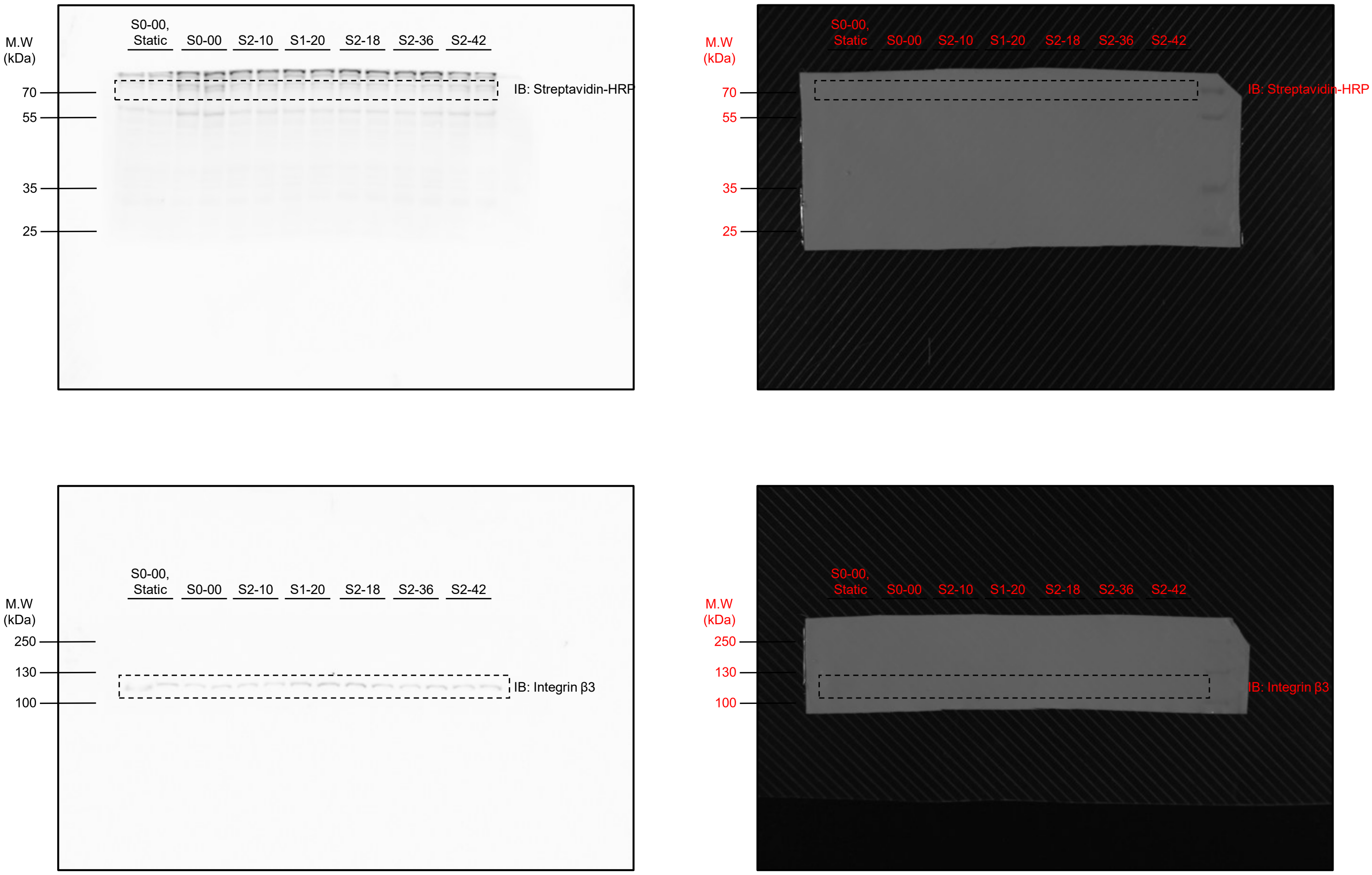
S0-00, Static
S0-00
S2-10
S1-20
S2-18
S2-36
S2-42
M.W (kDa)
70
55
35
25
IB: Streptavidin-HRP
S0-00, Static
S0-00
S2-10
S1-20
S2-18
S2-36
S2-42
M.W (kDa)
70
55
35
25
IB: Streptavidin-HRP
S0-00, Static
S0-00
S2-10
S1-20
S2-18
S2-36
S2-42
M.W (kDa)
250
130
100
IB: Integrin β3
S0-00, Static
S0-00
S2-10
S1-20
S2-18
S2-36
S2-42
M.W (kDa)
250
130
100
IB: Integrin β3

Figure S3A and S4A

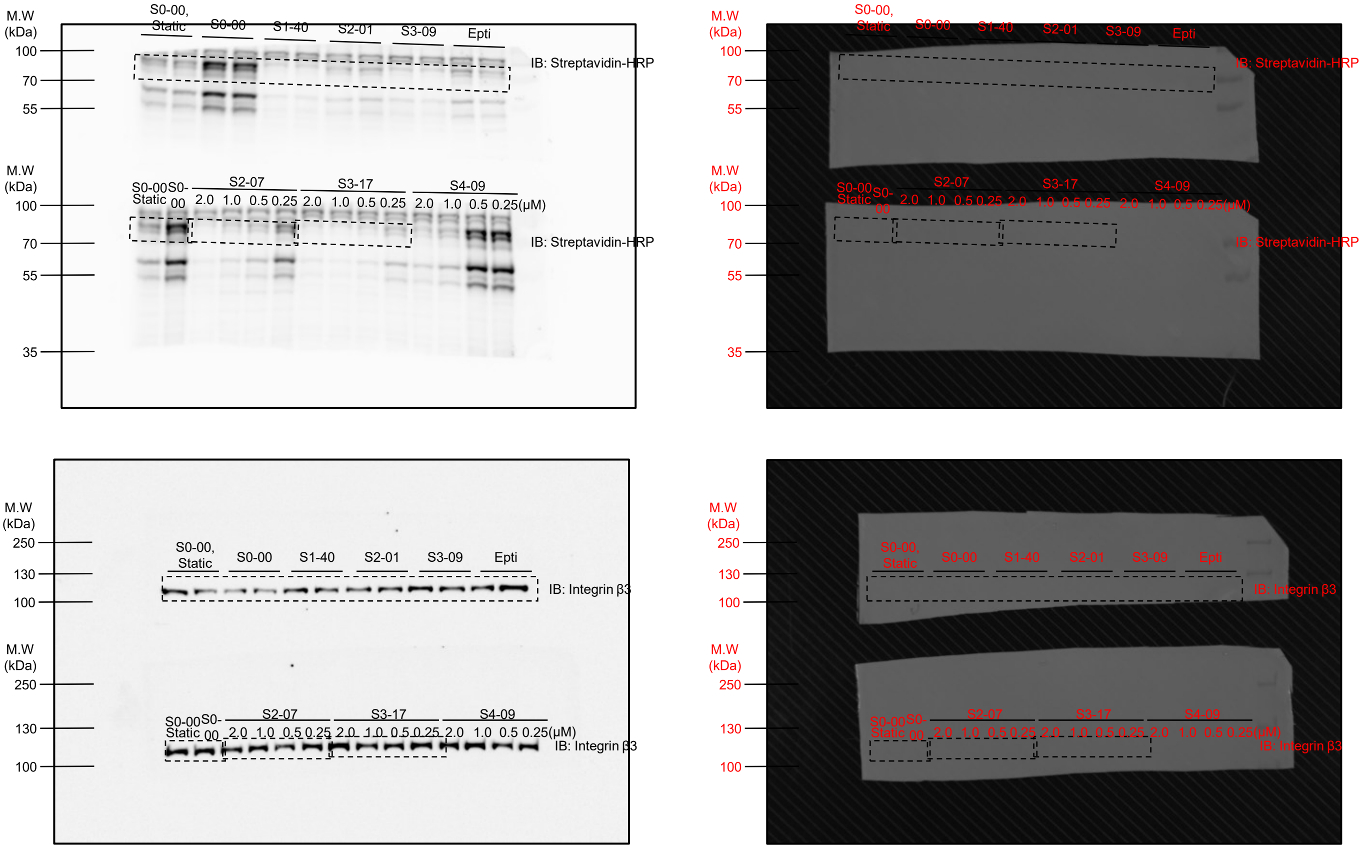

Figure S3A

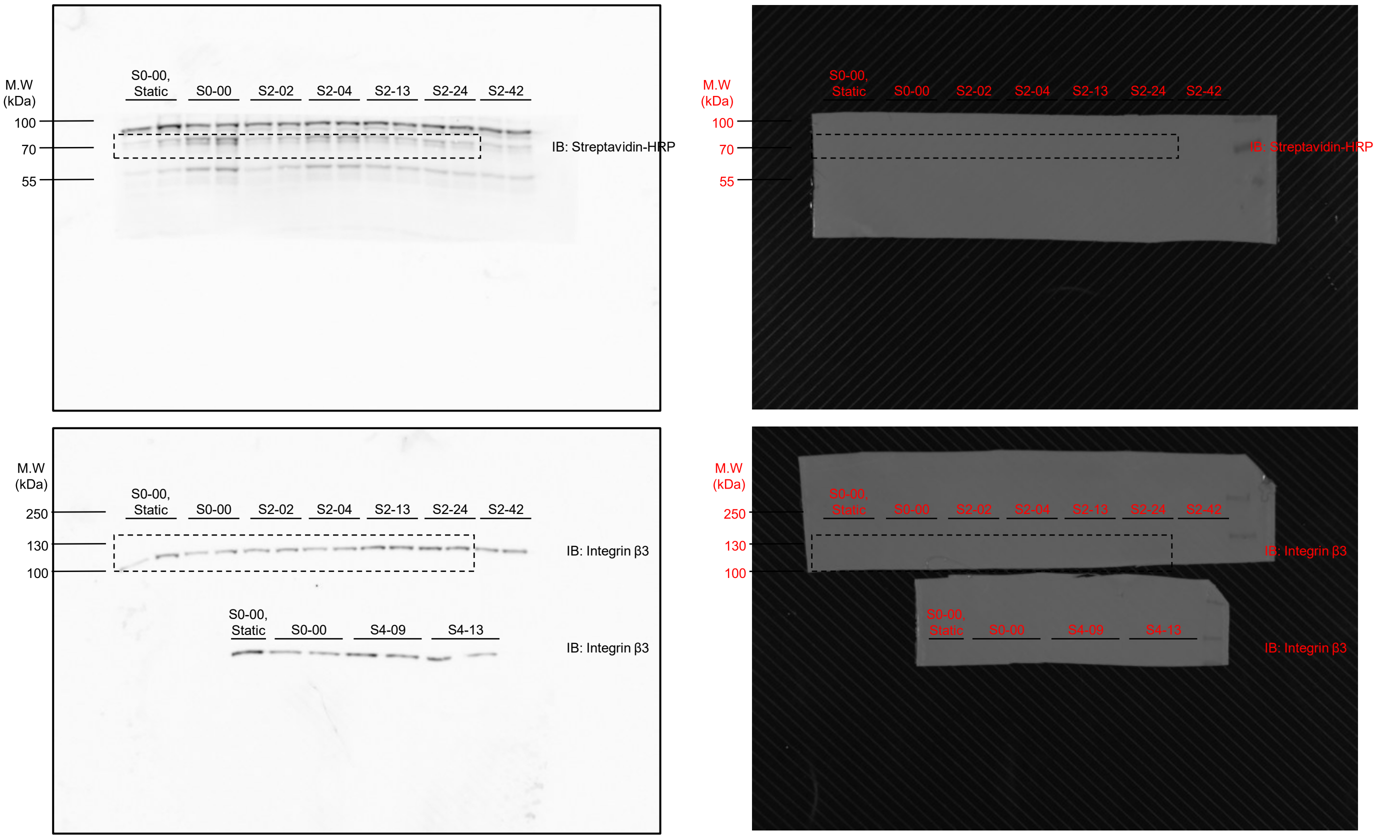
M.W (kDa)
100
70
55
S0-00, Static
S0-00
S2-02
S2-04
S2-13
S2-24
S2-42
IB: Streptavidin-HRP
M.W (kDa)
250
130
100
S0-00, Static
S0-00
S2-02
S2-04
S2-13
S2-24
S2-42
IB: Integrin β3
S0-00, Static
S0-00
S4-09
S4-13
IB: Integrin β3

Figure S3A

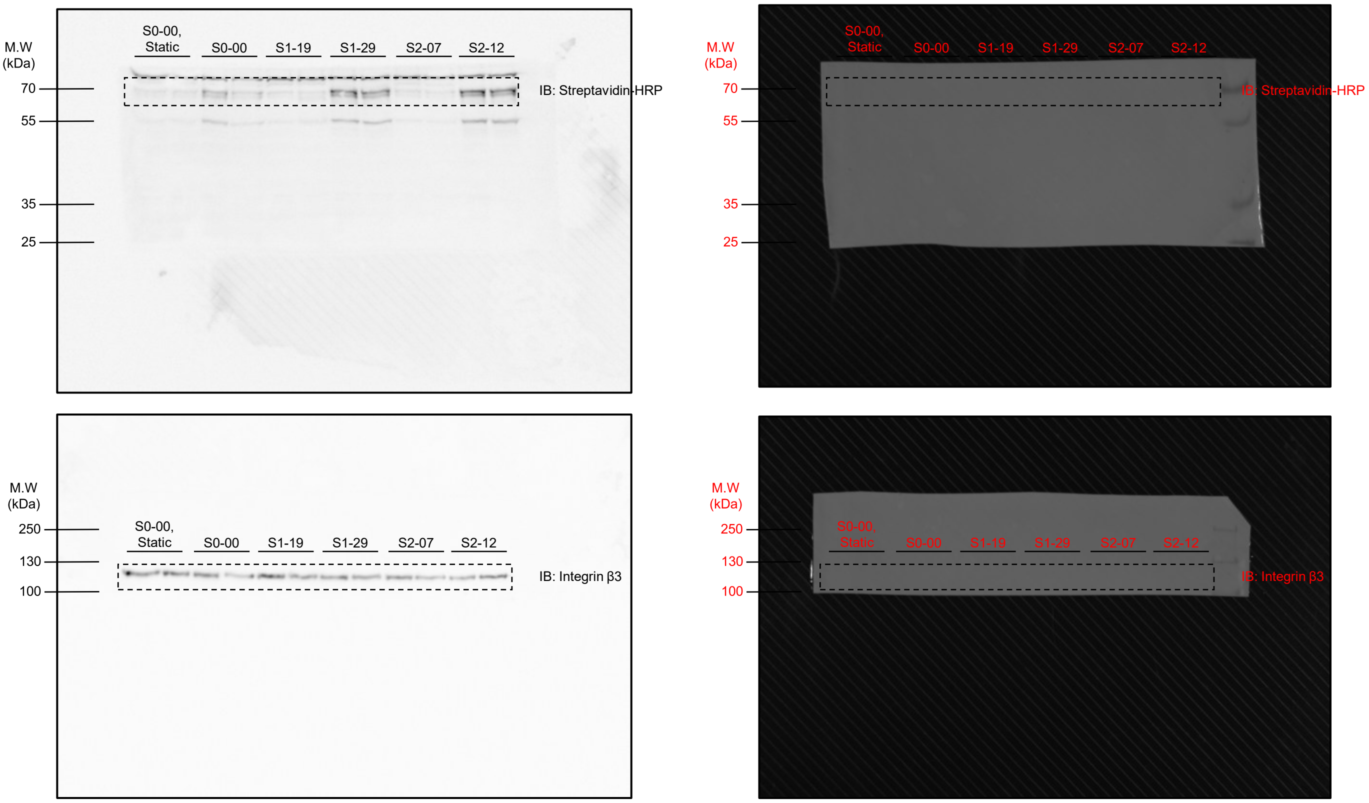

M.W (kDa)
70
55
35
25
S0-00, Static
S0-00
S1-19
S1-29
S2-07
S2-12
IB: Streptavidin-HRP
M.W (kDa)
250
130
100
IB: Integrin β3

Figure S3A

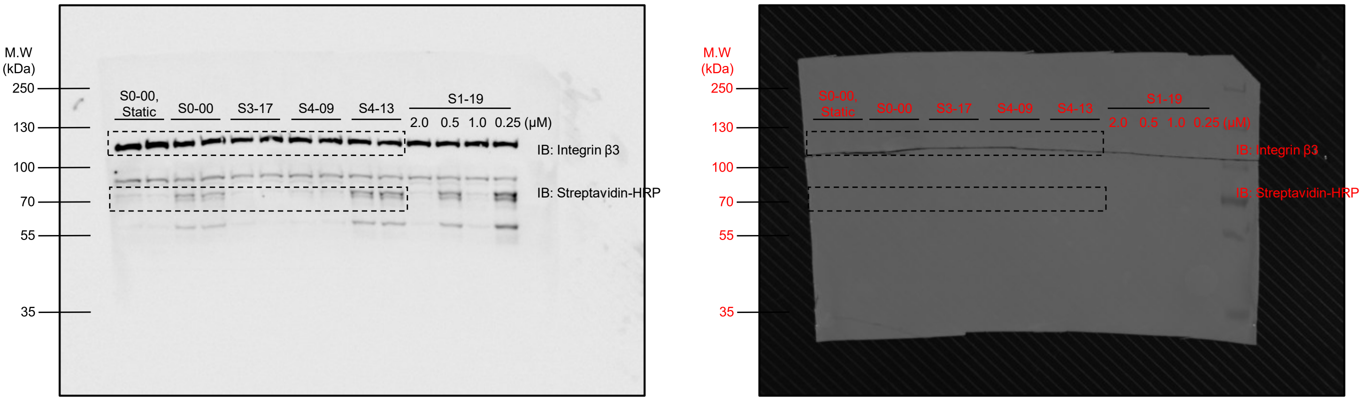

M.W (kDa)
250
130
100
70
55
35
S0-00, Static
S0-00
S3-17
S4-09
S4-13
S1-19
2.0 0.5 1.0 0.25 (μM)
IB: Integrin β3
IB: Streptavidin-HRP
M.W (kDa)
250
130
100
70
55
35
S0-00, Static
S0-00
S3-17
S4-09
S4-13
S1-19
2.0 0.5 1.0 0.25 (μM)
IB: Integrin β3
IB: Streptavidin-HRP

Figure S4A

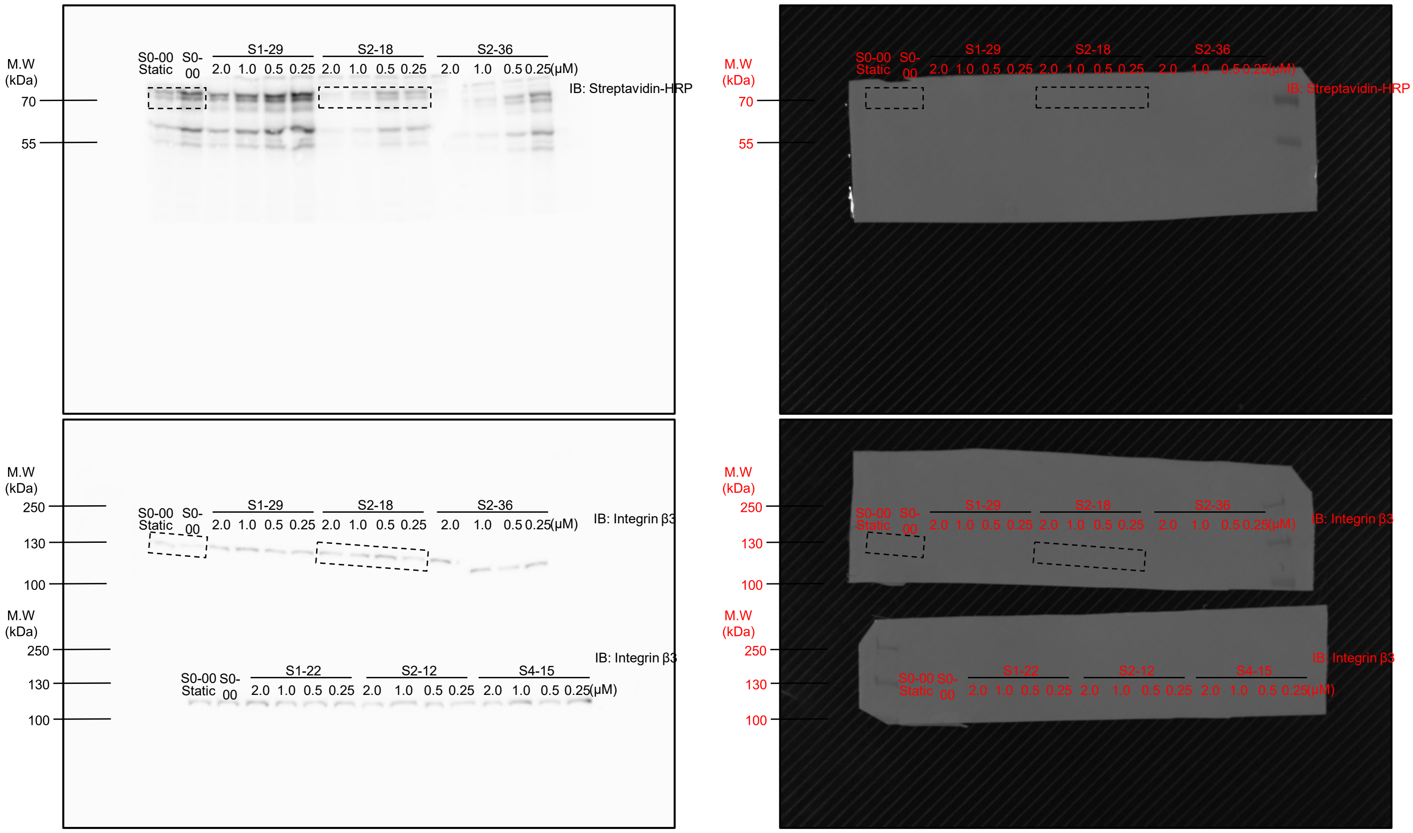
M.W
(kDa)
70
55
S0-00 Static
S0-00
S1-29
S2-18
S2-36
2.0 1.0 0.5 0.25 2.0 1.0 0.5 0.25 2.0 1.0 0.5 0.25(μM)
IB: Streptavidin-HRP
M.W
(kDa)
250
130
100
IB: Integrin β3
S1-22
S2-12
S4-15

Figure S4A

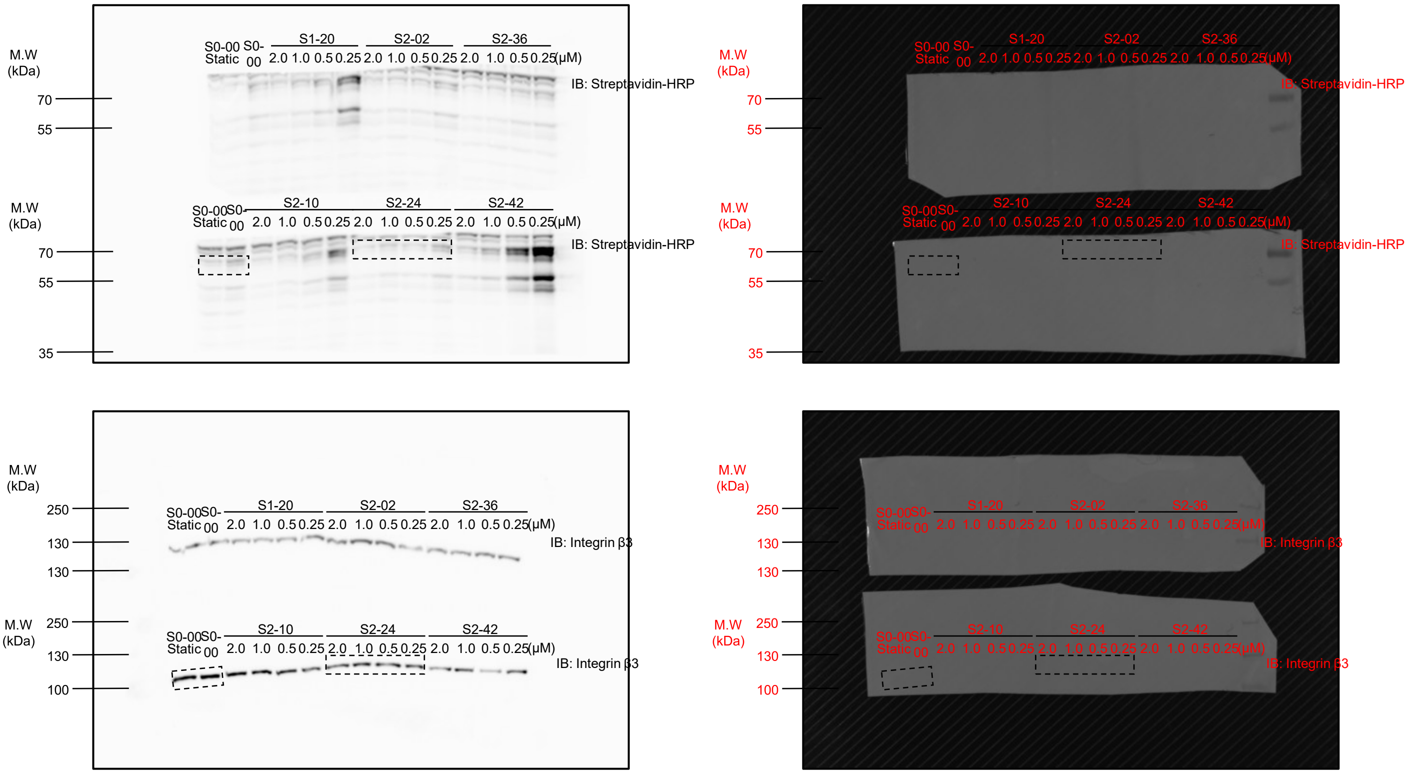
M.W (kDa)
S0-00 Static
S0-00
S1-20
S2-02
S2-36
2.0 1.0 0.5 0.25 (μM)
IB: Streptavidin-HRP
70
55
S2-10
S2-24
S2-42
35
250
130
100
IB: Integrin β3

Figure S7A

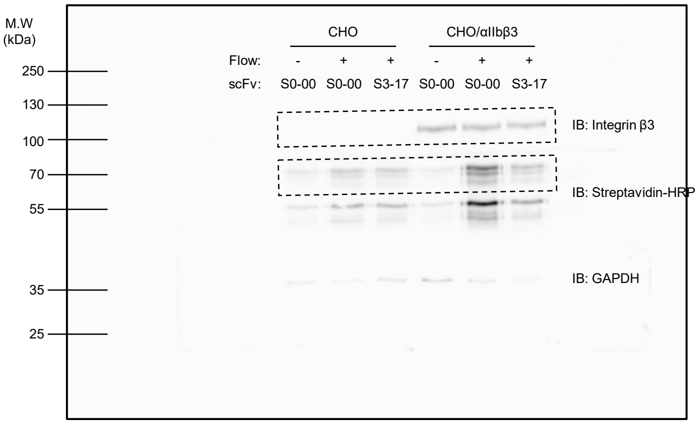
M.W (kDa)
250
130
100
70
55
35
25
CHO
CHO/αIIbβ3
Flow: - + + - + +
scFv: S0-00 S0-00 S3-17 S0-00 S0-00 S3-17
IB: Integrin β3
IB: Streptavidin-HRP
IB: GAPDH

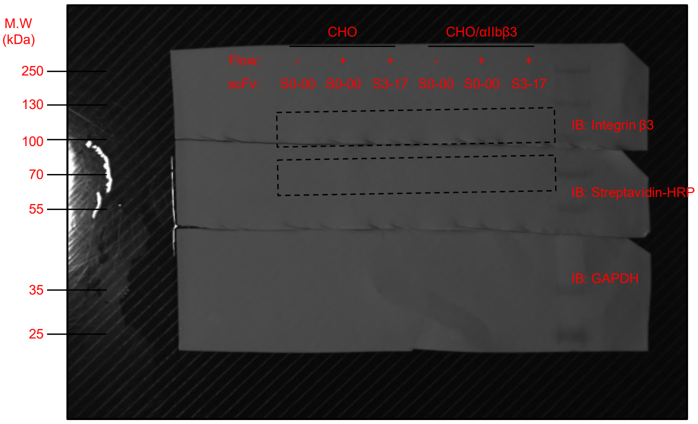
M.W (kDa)
250
130
100
70
55
35
25
CHO
CHO/αIIbβ3
Flow: - + + - + +
scFv: S0-00 S0-00 S3-17 S0-00 S0-00 S3-17
IB: Integrin β3
IB: Streptavidin-HRP
IB: GAPDH

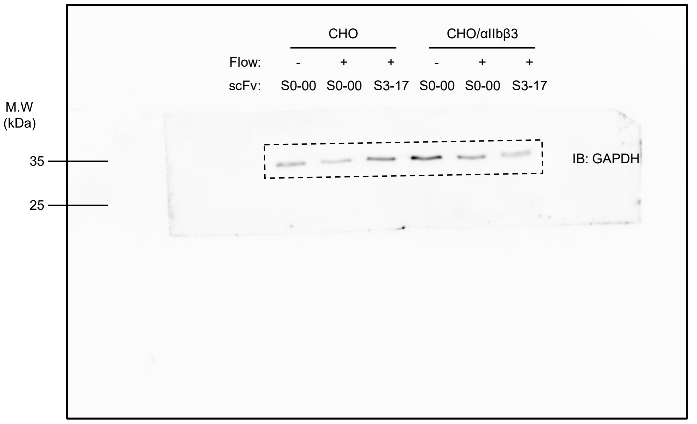
CHO
CHO/αIIbβ3
Flow: - + + - + +
scFv: S0-00 S0-00 S3-17 S0-00 S0-00 S3-17
M.W (kDa)
35
25
IB: GAPDH

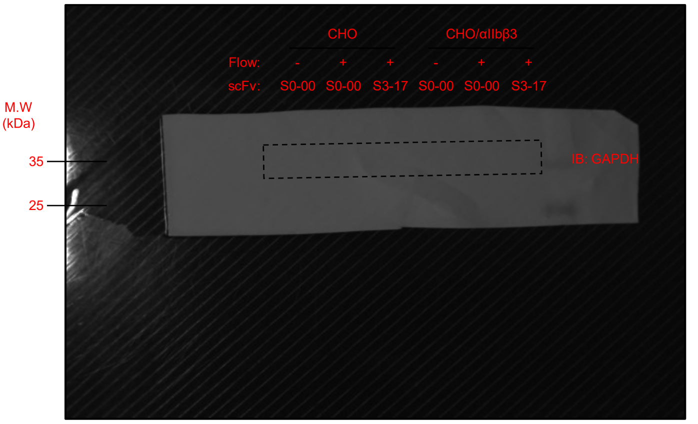
CHO
CHO/αIIbβ3
Flow: - + + - + +
scFv: S0-00 S0-00 S3-17 S0-00 S0-00 S3-17
M.W (kDa)
35
25
IB: GAPDH

Figure S7C

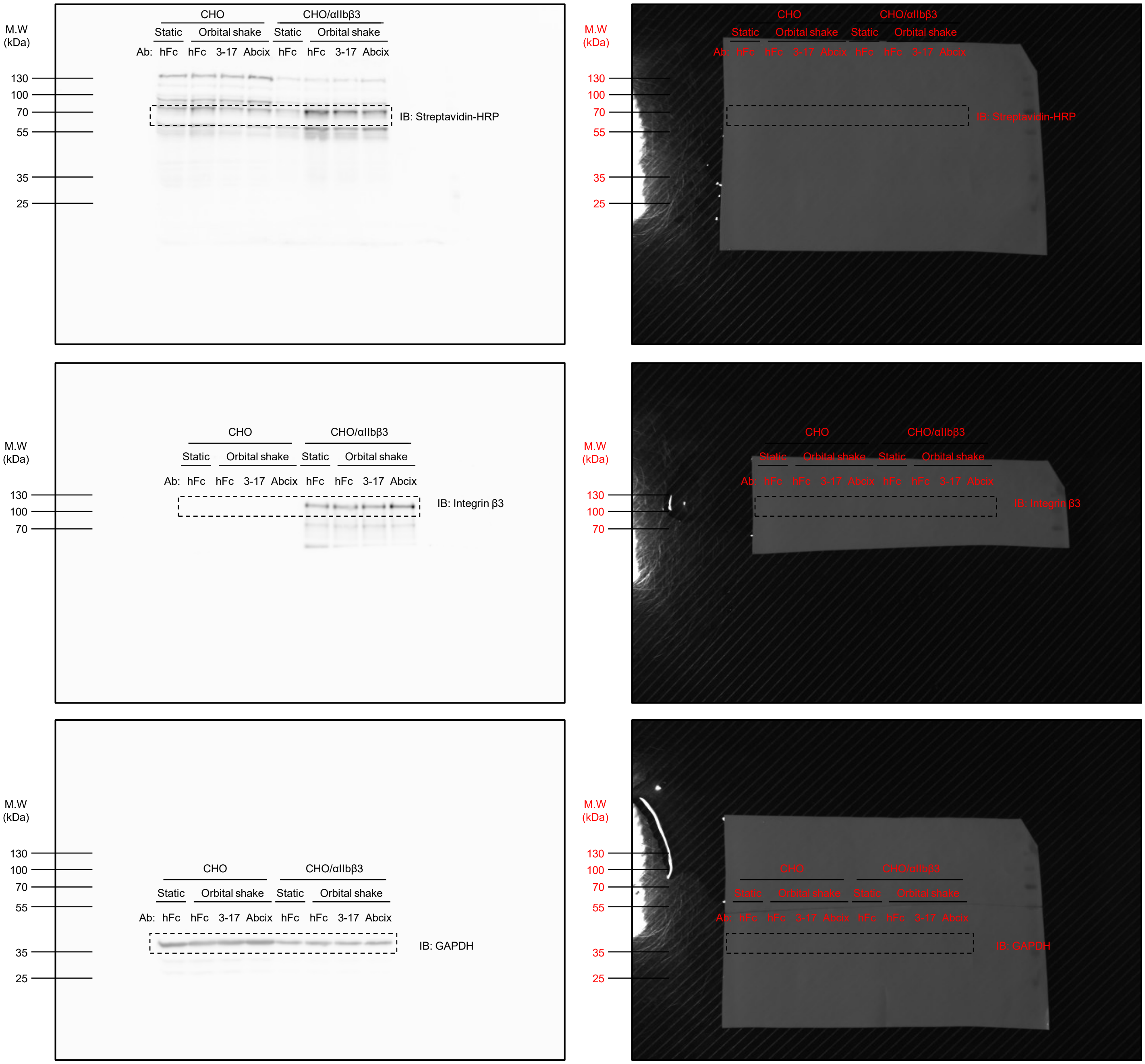

M.W (kDa)
130
100
70
55
35
25
CHO
CHO/αIIbβ3
Static
Orbital shake
Ab: hFc hFc 3-17 Abcix hFc hFc 3-17 Abcix
IB: Streptavidin-HRP
IB: Integrin β3
IB: GAPDH

Figure S8B

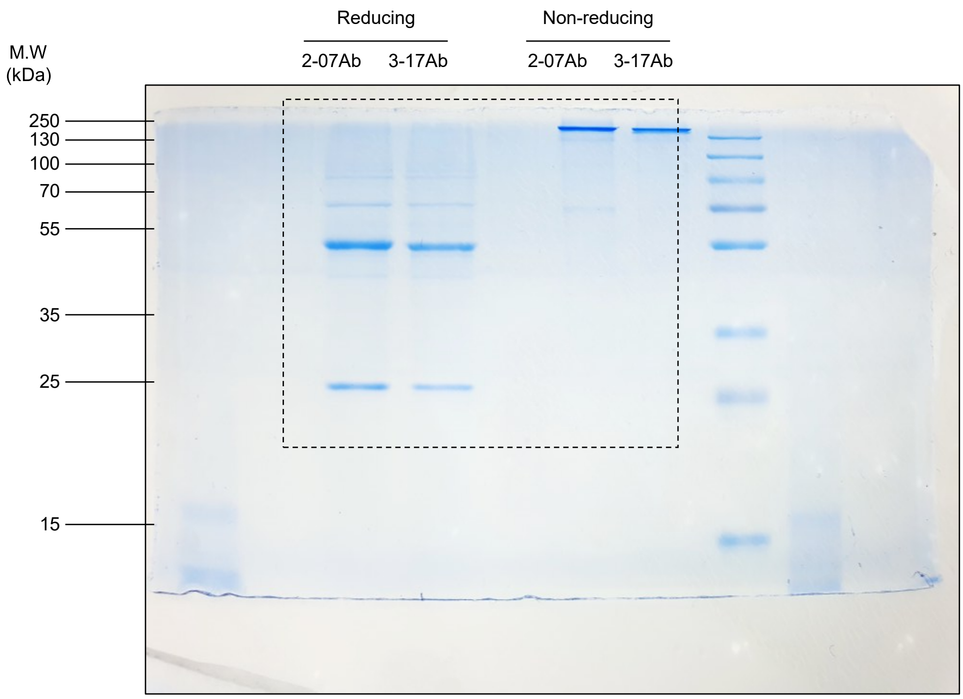

Reducing
Non-reducing
2-07Ab
3-17Ab
2-07Ab
3-17Ab
M.W
(kDa)
250
130
100
70
55
35
25
15

Figure S8E

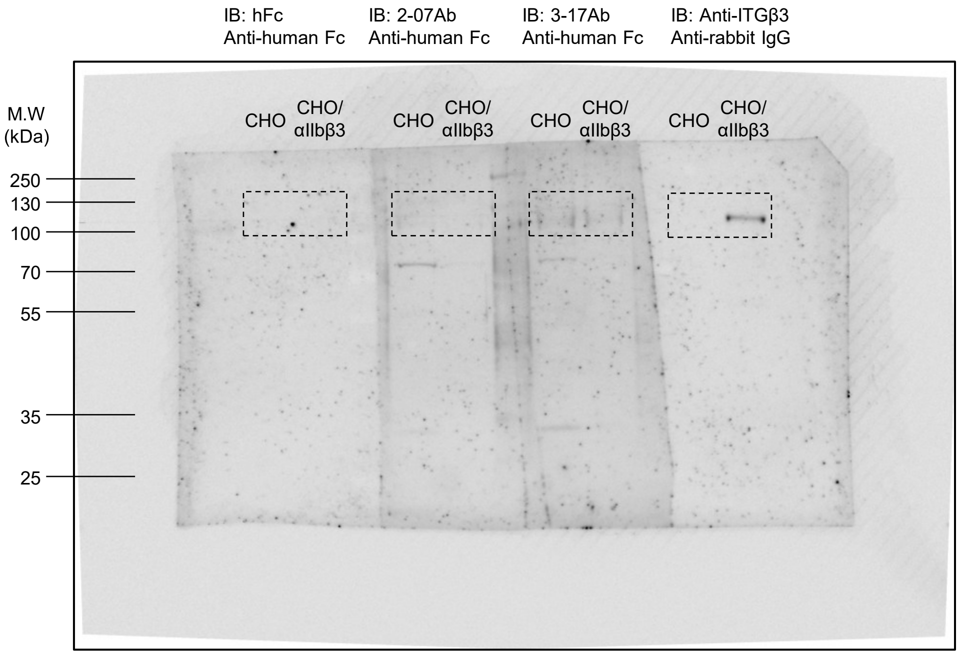
IB: hFc
Anti-human Fc
IB: 2-07Ab
Anti-human Fc
IB: 3-17Ab
Anti-human Fc
IB: Anti-ITGβ3
Anti-rabbit IgG
M.W
(kDa)
CHO
CHO/
αIIbβ3
CHO
CHO/
αIIbβ3
CHO
CHO/
αIIbβ3
CHO
CHO/
αIIbβ3
250
130
100
70
55
35
25

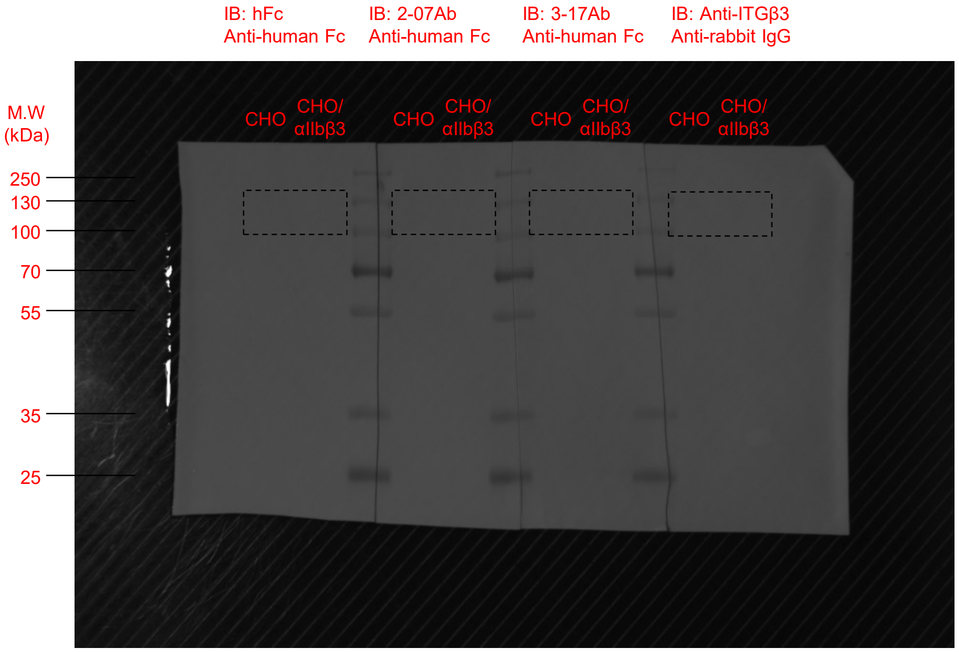
IB: hFc
Anti-human Fc
IB: 2-07Ab
Anti-human Fc
IB: 3-17Ab
Anti-human Fc
IB: Anti-ITGβ3
Anti-rabbit IgG
M.W
(kDa)
CHO
CHO/
αIIbβ3
CHO
CHO/
αIIbβ3
CHO
CHO/
αIIbβ3
CHO
CHO/
αIIbβ3
250
130
100
70
55
35
25
